# Self-Recoverable, Energy-Dissipating, and Healable Chain-Extended Supramolecular Polyurethanes and Poly(urethane–urea)s for Impact-Resistant Systems

**DOI:** 10.1021/acsami.6c06182

**Published:** 2026-07-01

**Authors:** Alarqam Z. Tareq, Matthew Hyder, Peihao Song, Georgios Kalimeris, Thomas Zinn, James E. Hallett, Ann M. Chippindale, Clive R. Siviour, Wayne Hayes

**Affiliations:** a Department of Chemistry, 6816 University of Reading, Whiteknights, Reading RG6 6DX, U.K.; b Department of Chemistry, Faculty of Science, University of Zakho, Duhok 42001, Iraq; c Department of Engineering Science, 6396 University of Oxford, Parks Road, Oxford OX1 3PJ, U.K.; d Diamond Light Source, Diamond Light Source Ltd., Harwell Science & Innovation Campus, Didcot OX11 0DE, U.K.

**Keywords:** supramolecular polyurethane, dynamic covalent network, disulfide, energy dissipation, shape memory, self-healing

## Abstract

Materials utilized for impact protection must be capable of effective absorption and dissipation of external impact forces as well as being lightweight, flexible, and comfortable. One strategy to meet these stringent criteria is through the exploitation of adaptive dynamic networks. Herein, we report a series of chain-extended supramolecular polyurethane and poly­(urethane–urea) elastomers, which utilize both hydrogen bonding interactions and disulfide units to impart structural stability, reprocessability, and reversible adaptability. The key design element of these phase-separated materials is the use of two distinct dynamic bonding mechanisms within the hard domains, which serve to efficiently reinforce the elastomeric assemblies. The optimum elastomer exhibited excellent self-recoverability (91% and 99%) after relaxing for 30 and 60 s, respectively, at 20% compression. During loading–unloading compression cycles, the elastic recovery ratios of this elastomer reached 77 ± 0.3% with dissipated energies of 235,000 J m^–3^ at a deformation of 80%. This study highlights how the combination of noncovalent interactions and dynamic covalent bonding can be utilized to generate elastomers capable of rapid autonomous healing and property recovery while simultaneously providing exceptional energy dissipation, paving the way for the impact-resistant systems.

## Graphical abstract

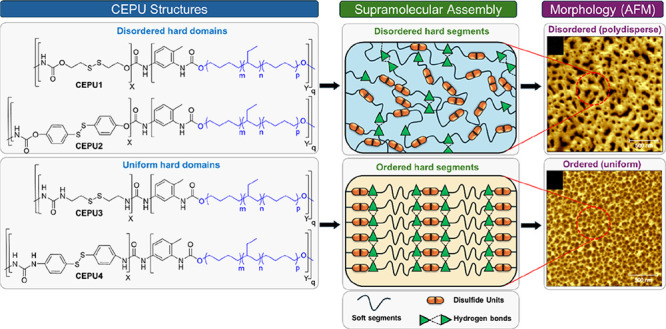

## Introduction

With the ever-increasing demand for impact-resistant and elastomeric polymers, supramolecular chemistry has been used in combination with adaptive dynamic networks,^1–3^ such as (retro-)­Diels–Alder (DA) reactions,^4^ urethane^5^ and urea^6^ reversions, addition–fragmentation chain transfer (AFT),^7^ and disulfide^8^ and thiol–disulfide^9^ exchange to create innovative materials. Through the incorporation of dynamic covalent bonds^10,11^ in polymer backbones, materials can be developed for applications such as energy storage,^12,13^ energy dissipation,^14,15^ 3D printing,^16,17^ and regenerative medicine and responsive protein materials.^18,19^ Furthermore, noncovalent interactions such as hydrogen bonding can instil material properties, such as self-recovery^20^ and self-healing,^21^ enabling generation of materials with extended service life, durability, and reliability.^6,22^

The thermodynamic nature of the chain-extender units based on reversible chemistries such as reversible addition and degenerative exchange is necessary to design and develop the next generation of dynamic materials.^1^ For example, a library of dynamic and amorphous polyamide materials^23^ based on covalent adaptable networks have been developed by Du Prez and co-workers, with a rapid temperature response in the range of 150–200 °C allowing for good reprocessability. Recently, Bowman and co-workers combined dynamic covalent chemistry and dual-cure methodology to develop photosensitive elastomer materials that are topographically controlled via mechanophotopatterning.^24^

Within the field of dynamic covalent bonding, disulfide bonds have attracted significant attention as a result of their ability to undergo reversible homolytic cleavage and reformation through tunable addition and/or metathesis mechanisms via degenerative bonds.^25,26^ This dynamic exchange enables the reconfiguration of the polymer network during deformation, imparting these materials with advantageous self-healing and self-recovery capabilities.^27,28^ A notable example by Ding and co-workers reported the development of dynamic supramolecular ionic conductive elastomers,^29^ achieved using a phase-locking strategy in which these autonomously self-healing polymers combine dynamic disulfide units capable of metathesis with hydrogen bonding from 2-ureido-4-pyrimidone (UPy) units^30^ incorporated into the polymer backbone. Rossegger and co-workers have also exploited the wavelength-dependent dynamic behavior of disulfide bonds during UV curing of thiol–ene networks, defining the dynamic properties of the resulting photopolymer.^8^

Polymeric materials, which possess high-strength with self-reinforcing and toughening properties, are of industrial relevance in a broad range of advanced applications; noncovalent interactions offer a promising avenue to impart such characteristics.^31,32^ High impact-stiffening and rapid soft-to-rigid transformation supramolecular polymers^15^ have been reported by Wu and co-workers, via guanidinium-carboxylate salt-bridge hydrogen bonds between poly­(α-thioctic acid) and arginine clusters. Significant microphase separation and varying molecular architectures of supramolecular poly­(urethane–urea) elastomers^33^ have been reported by Wang and co-workers, utilizing a high density of hydrogen bonds to exhibit excellent impact resistance and energy dissipation under dynamic loading conditions. Cyclic loading and load–relax–reload are commonly used as mechanical characterization methods to quantify hysteresis and energy absorption during deformation. These tests help distinguish reversible viscoelasticity recovery and nonrecoverable softening (Mullins-like effects) on the experimental time scale.^34–37^ In addition, TPUs exhibit pronounced rate-dependent behavior, and their energy absorption has been found to vary with the loading rate.^38,39^

In previous studies, we have developed thermally and mechanically robust supramolecular polyurethanes^40–42^ with synergetic multifunctional hydrogen bonding end-caps. Recently, a series of novel chain-extended and cross-linked polyurethanes featuring a commercially available and degradable chain-extender unit have been synthesized and used as polymerizable coatings and hot-melt adhesives.^43,44^ Inspired by the above studies and cognizant of the potential of commercially available aliphatic and aromatic disulfides as dynamic reversible chain-extenders, herein, we report the design and synthesis of novel chain-extended polyurethane and poly­(urethane–urea) (CEPU) elastomers. These polymeric materials integrate dynamic disulfide exchange and supramolecular hydrogen bonding interactions to construct adaptable networks, which exhibit shape memory behavior, efficient energy dissipation, and self-healing capabilities ideal for use in impact-resistant systems.

## Experimental Section

### Materials

Krasol HLBH-P 2000 (molecular weight as supplied = 2100 g mol^–1^) was kindly provided by Total Cray Valley, all other reagents used were purchased from Sigma-Aldrich, Tokyo Chemical Industry, Fisher Scientific, and Fluorochem. THF was dried directly prior to use with an MBRAUN SP7 system fitted with activated alumina columns.

The full synthetic protocols used to generate the CEPU elastomers and low-molecular-weight disulfide analogues together with the associated characterization and AFM images are provided in the Supporting Information (SI) file, Figures S1–S26.

### Characterization

#### Nuclear Magnetic Resonance Spectroscopy (NMR)

^1^H NMR and ^13^C NMR spectra were measured using either a Bruker Nanobay 400 or a Bruker DPX 400 spectrometer operating for ^1^H NMR (400 MHz) or ^13^C NMR (100 MHz) spectroscopic analysis. Chemical shifts (δ) are reported in ppm relative to the residual solvent resonances for DMSO-*d*_6_ (δ 2.50 ppm) and THF-*d*_8_ (δ 3.58 ppm) in ^1^H NMR spectra.

#### Fourier-Transform Infrared Spectroscopy (FT-IR)

FT-IR analysis was carried out at room temperature using a PerkinElmer 100 FT-IR instrument equipped with a diamond-ATR sampling accessory.

#### Raman Spectroscopy

Raman spectra were measured by a LabRAM Soleil Raman microscope at room temperature, with laser excitation at 785 nm (100 mW), 30 s delay, 15 s acquisition, 5 time acceleration, 600 grooves, and a hole size of 200 μm; Spectra was recorded between 400 and 700 cm^–1^; spectrometer calibration, background subtraction, and spectra editing were set using LabSpec 6 spectroscopy software version 6.7.2.17. A micromechanical tensile tester (Deben) (maximum load 200 N) was used to stretch the samples with a constant velocity of 1 mm/s, and the data were analyzed using Microtest v5.3.36 software.

#### Mass Spectrometry (MS)

MS was conducted using a Thermo Fisher Scientific Orbitrap XL LCMS. The sample was introduced by liquid chromatography (LC), and sample ionization was achieved by electrospray ionization (ESI).

#### Gel Permeation Chromatography (GPC)

The average molecular weights of the polymers generated were determined using an Agilent Technologies 1260 Infinity I system in HPLC-grade THF at a flow rate of 1.0 mL min^–1^. Calibration was achieved using a series of near-monodisperse polystyrene standards, and samples were prepared at a concentration of 1.0 mg mL^–1^.

#### Atomic Force Microscopy (AFM)

AFM images were obtained using a Cypher S AFM (Oxford Instruments-Asylum Research, Santa Barbara, USA) at the University of Reading. The AFM stage movement within the *x*, *y*, and *z* directions was controlled using piezoelectric stacks. The scans were recorded through the user interface, Igor Pro (version 16.33.234), using the standard alternating contact (AC) topography mode (tapping mode) operating in air using a silicon tip with a resonant frequency set at approximately 70 kHz and a spring constant of approximately 2.0 N m^–1^ (AC240TS-R3, Oxford Instruments). Each sample was drop-cast onto a 10 mm diameter AFM mica disc, first cleaved with Sellotape. Each disk was mounted onto 15 mm diameter magnetic stainless-steel AFM specimen disks using 9 mm diameter carbon adhesive tabs and secured onto the microscope scanner stage magnetically. Then, through the user interface, the objective focus was adjusted and set to focus on the tip and each sample in turn. The cantilever was autotuned at its resonance, which automatically determined the drive amplitude and drive frequency. The resolution, scan rate, integral gain, and scan size were entered into the user interface before starting the scan. The software Gwyddion (ver. 2.63) was used for data analysis and editing.

#### Single-Crystal X-ray Diffraction

Crystals of analogues **1**–**4** were mounted under Paratone-N oil and flash-cooled to 100 K under nitrogen in an Oxford Cryosystems Cryostream. Single-crystal X-ray intensity data were collected using a Rigaku XtaLab Synergy diffractometer (Cu Kα (λ = 1.54184 Å)). Data were reduced within the CrysAlisPro software.^45^ Structures were solved using the program Superflip,^46^ and all nonhydrogen atoms were located. Least-squares refinement against *F* ^2^ was carried out using the CRYSTALS suite of programs.^47^ Nonhydrogen atoms were refined anisotropically. All hydrogen atoms were located in difference Fourier maps. The positions of the hydrogen atoms attached to nitrogen were refined with a *U*_iso_ of ∼1.2–1.5 times the value of *U*_eq_ of the parent N atom with an N–H distance restrained to be 0.85(1) Å. The hydrogen atoms attached to carbon were placed geometrically with a C–H distance of 0.95 Å and a *U*_iso_ of ∼1.2–1.5 times the value of the *U*_eq_ of the parent C atom, and the positions refined with riding constraints.

#### Differential Scanning Calorimetry (DSC)

DSC measurements were performed on a TA Instruments X3 DSC adapted with a TA refrigerated cooling system (RCS90), using aluminum TA Tzero pans and hermetic lids, measuring from −80 to 200 °C with heating and cooling rates of 10 °C min^–1^ under nitrogen gas with a flow rate of 50 mL min^–1^. The sample was analyzed (using the TRIOS software (version 5.1.1)) over three heating–cooling cycles. Glass transition temperatures (*T*_g_) are reported as the midpoint of the transition.

#### Thermogravimetric Analysis (TGA)

TGA was carried out on TA Instruments TGA Q50 instrument with aluminum Tzero pans. The sample was heated from 20 to 600 °C at 10 °C min^–1^ under nitrogen gas with a flow rate of 60 mL min^–1^. TGA of samples was investigated using the TRIOS software (version 5.1.1).

#### Rheology

Rheological measurements were performed on a Malvern Panalytical Kinexus Lab+ instrument fitted with a Peltier plate cartridge and 8 mm parallel-plate geometry and analyzed using rSpace Kinexus v1.76.2398 software.

#### Tensiometry

Tensile tests were carried out using a Thümler Z3-X1200 tensometer at a rate of 10 mm min^–1^ with a 1 kN load cell and analyzed using THSSD-2026 software.

#### Optical Microscopy

Videos were captured using a Leica DM1000 microscope equipped with a Mettler Toledo FP82 hot stage. The sample was placed onto a glass slide and then placed into the hot stage chamber, the temperature of which was controlled by an FP90 central processor (heating rate 10 °C min^–1^). All videos were recorded using Studio86Designs software.

#### Variable-Temperature Small-Angle X-ray Scattering (SAXS) and Wide-Angle X-ray Scattering (WAXS)

VT-SAXS and WAXS experiments were performed on a Bruker Nanostar instrument at the University of Reading, equipped with Incoatec Microfocus Source IμS with an energy of 8.04 keV corresponding to a wavelength of 1.54Å. Data were collected on a photon counting Vantec 500 with a sample-to-detector length of 660 mm. The beam size was 0.5 mm diameter on the sample. Silver behenate (layer spacing, *d* = 58.38 Å) was used to calibrate the SAXS data. Samples (polymer film) were mounted in modified DSC pans equipped with Kapton windows and mounted in an MRI electrical heating unit for temperature control.

#### 1D- and 2D-SAXS

Patterns were recorded at the Diamond Light Source (Harwell, U.K.) using a Xeuss 3.0 SAXS instrument (Xenocs, France) equipped with a liquid gallium and indium alloy Metal Jet X-ray source (Excillum, Sweden, λ = 0.134 nm) operating at 70 kV, two sets of motorized scatterless slits for beam collimation (high flux 1.10 × 10^6^ ph/s), and an Eiger2R1Mpixel detector (sample-to-detector distance = 515 mm). The sample-to-detector distance was calibrated using a silver behenate standard in transmission geometry. SAXS patterns were recorded from *q* = 0.12 to 3.70 nm^–1^, for which *q*= (4π sin θ)/λ is the length of the scattering vector and θ is one-half of the scattering angle. A micromechanical tensile tester (maximum load 40N) was used to stretch the samples with a constant velocity of 0.1 mm/s.

Data were reduced (normalized, integrated, and averaged) using a standard using Dawn software developed at the Diamond Light Source,^48,49^ and fitting was achieved using SasView version 5.0.6 (www.sasview.org/) using a shape-independent broad peak function to obtain *q*_max_.

The scattering intensity (*I*) in a shape-independent broad peak model was calculated using eq 1:

$$I=\frac{A}{q^{n}}+{\left(\frac{C}{1+(|q-q_{0}|\xi {)}^{m}}\right)}^{p}+B$$ 1

where *A* is the Porod law scale factor, *q* is the scattering vector, *n* is the Porod exponent, *C* is the Lorentzian scale factor, *q*_0_ is the peak position, *m* is the exponent of *q*, ξ is the screening length, *B* is the flat background, and *p* generalizes the model to allow interpolation between a Lorentzian and Debye–Anderson–Brumberger (DAB) peak. *d*-spacing was calculated using eq 2:

$$d=\frac{2\pi }{q_{0}}$$ 2

#### Tension Tests

Uniaxial tension experiments were performed for **CEPU3** and **CEPU4** using an Instron model 5582 screw-driven load frame with a 1 kN load cell at 24 °C. Displacement control was used at a rate of 10 mm/min.

#### Compression Tests

Due to the very low stiffness of **CEPU1** and **CEPU2**, our mechanical testing focused on **CEPU3** and **CEPU4**. All specimens were cut from sheets with a thickness of approximately 2.0 ± 0.1 mm. Compression specimens had a diameter of 5.0 ± 0.1 mm. To ensure repeatability, each test was repeated at least three times.

Three mechanical test machines were employed in this study, Quasi-static tests were performed using a screw-driven machine Instron 5980, with 1 kN load cell and compression anvils, under true strain rate control at a true strain rate of 0.01 s^–1^. Medium-rate tests were conducted on a hydraulic testing machine at an approximate strain rate of 10 s^–1^. High-rate tests (∼2500 s^–1^) were performed using a split-Hopkinson pressure bar. A schematic of the setups is shown in Figure 5D. Readers are referred to the literature for a more detailed review of the SHPB system.^50–52^ A gas gun launches the Ti-6AL-4V striker bar to generate the input wave in the input bar, and this input stress wave is propagated along the input bar to the specimen, which is sandwiched between the Ti-6AL-4V input and output bars. The input and reflected waves are measured using the strain gauges attached to the input bar, and the transmitted wave can be obtained from the strain gauges on the output bar.

## Results and Discussion

### Structure Design and Characterization of CEPU Elastomers

The synthetic approaches used to afford the CEPUs, shown in Figure 1, employ simple one-pot procedures to generate materials with dynamic bonding interactions. The selection of the polyol, diisocyanate, and chain-extender units is very important. Krasol HLBH-P 2000 (*M*_n_ = 2100 g mol^–1^) was used as the soft segment; the low glass transition temperature (*T*_g_) (−46 °C) of this polyol provides flexible chains in the resultant polymer to permit deformability. The hard phase consisted of tolylene-2,4-diisocyanate (2,4-TDI) and dynamic chain-extenders including aliphatic disulfides (bis­(2-hydroxyethyl) disulfide and cystamine) (**CEPU1** and **CEPU3**) or aromatic disulfides (bis­(4-hydroxyphenyl) disulfide and 4-aminophenyl disulfide) (**CEPU2** and **CEPU4**), which improved the dynamic behavior and the capability of the final polymer to dissipate energy under impact processes as well as good shape fixity. The reaction conditions are mild, controllable, and easily scaled to generate products in quantities >hundreds of grams.

**Figure 1:**
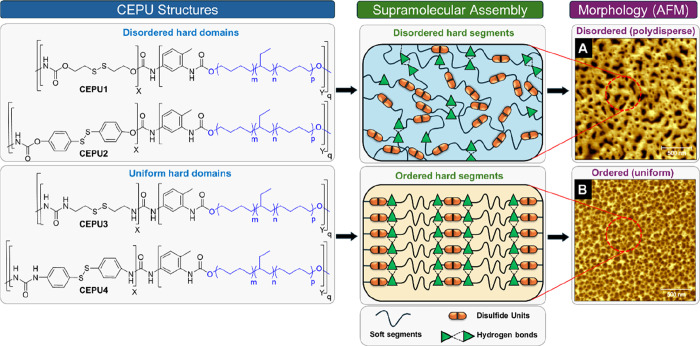
Structures of **CEPU1**–**CEPU4** elastomers, their supramolecular assembly via hydrogen bonding, and AFM images (phase mode) of (A) **CEPU1** and (B) **CEPU4**.

The association and dissociation of hydrogen bonds, together with the reversibility of the disulfide units in the hard phase, contribute significantly to the mechanical, rheological, toughness, and impact resistance of polymer materials.^53^ In this study, **CEPU1** and **CEPU2** were synthesized in a one-step polycondensation reaction, which resulted in weak phase separation stemming from the widely polydisperse hard domains and lack of nodal aggregation (i.e., association of the hard phase via hydrogen bonds). In contrast, **CEPU3** and **CEPU4** were synthesized via a two-step process, where an isocyanate-terminated prepolymer was first generated and then chain-extended by disulfide-functionalized diamines, which enhanced the phase separation.

FT-IR spectroscopy was used to monitor each reaction, and when the isocyanate band at 2272–2252 cm^–1^ disappeared, the reaction was considered complete. The FT-IR spectrum of CEPU elastomers exhibited characteristic absorption bands at ca. 3315–3232 cm^–1^, see Figure S11, attributed to N–H stretching vibrations, with strong bands at ca. 1701–1711 and 1636–1644 cm^–1^ assigned to C=O stretching of the urethane and urea functionalities. Absorptions at ca. 2950–2851 cm^–1^ confirmed aliphatic C–H bonds arising from the alkyl chain of the polyol. Atomic force microscopy (AFM) was performed to demonstrate the phase separation within the resultant CEPU elastomers. Each polymer sample revealed phase-separated morphologies consisting of dark areas (hard domains) and bright areas (soft domains). The hard regions in the CEPU elastomers are different in shape and size as a result of the polycondensation reaction process (two- vs. one-step) and the type of chain-extender unit employed. The molecular weights of the CEPU elastomers were investigated via GPC analysis; **CEPU3** and **CEPU4** displayed significant increases in the *M*_n_, *M*_w_, and dispersity (*Đ*) when compared to **CEPU1** and **CEPU2**, stemming from the increased reactivity of the primary amine groups of the disulfide chain-extenders.

To gain an insight into the association, self-assembly, and disulfide exchange mechanism of the CEPU elastomers, low-molecular-weight analogues (**1**–**4**) were designed and synthesized to mimic the extended polymer samples. Crystals of the model urethanes and ureas, which possess the disulfide bond, have been investigated by single-crystal X-ray diffraction (see the SI file for the solid-state structures, hydrogen bonding interactions, and the associated crystallographic data, Figures S27–S34 and Tables S2–S10). The solid-state structures of the urethane and urea models revealed hydrogen bonds between the urethane–urethane and urea–urea groups of the individual molecules. If these interactions and the orientation are translated to the assembly of the CEPU elastomers, then the phase separation and noncovalent associations between polymer chains could be enhanced and lead to reinforcement of the mechanical, rheological properties, and energy dissipation capability, as expected from our design principle. A ^1^H NMR spectroscopic study was used to monitor and confirm the disulfide chain exchange process between the model exchange analogues **3** and **4** that correspond to cystamine and 4-aminophenyl disulfide extender units, respectively. The product formation was analyzed, and key resonances were observed in ^1^H NMR spectra at 8.82, 8.69, 6.32, and 2.87 ppm assigned to the new formed compound (1-phenyl-3-(4-((2-(3-phenylureido)­ethyl)­disulfaneyl)­phenyl)­urea). During the reaction, the degree of disulfide exchange was calculated via the relative integrals as a function of time, and the formation of products in DMSO-*d*_6_ at 80 °C was 30%, 41%, and 44.5% after 30, 60, and 120 min, respectively, see Figure S35, indicating that the S–S bonds incorporated temperature responsivity via disulfide metathesis.^54^

A film (0.2 mm thickness) of **CEPU4** was analyzed using Raman spectroscopy, and the material was stretched to extensions ranging from 0 to 100%. Spectra were recorded at 10% intervals to assess the polymer network rearrangement by processes such as disulfide exchange during the uniaxial deformation. These experiments provide direct molecular-level evidence of stress-responsive disulfide chain exchange within the bulk polymer, see Figure S36A. During deformation the integrated peak area of a key characteristic Raman band at 485 cm^–1^ that corresponds to aromatic S–S stretching vibrations increased as the material was stretched, in addition to a slight shift of the absorbance to 489 cm^–1^ indicating activation of the disulfide chain exchange process. However, the stretching limitation of the micro tensiometer used (only up to 100%) limited full activation of the disulfide exchange mechanism during deformation, see Figure S36B, whereas the intensity of the C–H stretching vibrations (*ca*. 2850–3000 cm^–1^) remained constant throughout deformation. In addition, the intensity of the signal at 525 cm^–1^ attributed to the disordered S–S bonds increased, indicating redistribution and reorientation of S–S moieties associated with the dynamic reorganization of the hard segments under deformation.

The thermal, mechanical, and dynamic viscoelastic behaviors were investigated by thermogravimetric analysis (TGA), differential scanning calorimetry (DSC), tensile tests, and rheological measurements, see Figure 2. All the elastomers are thermally stable displaying high onsets of degradation between 275 and 300 °C, suggesting excellent heat resistance, see Figures S37–S40. The 5% thermal decomposition temperature (*T*_d_ 5%) is presented in Table S11, and all elastomers fully degraded once the environment reached 475 °C. The glass transition temperature (*T*_g_) and thermal transitions were characterized by DSC, see Figure S41–S44. All of the CEPU elastomers exhibited *T*_g_ at ca. −46 °C,^40–42^ which derive from the amorphous hydrogenated poly­(butadiene) blocks in the polymer backbone and proved independent of the hard domains. The DSC plots of all the elastomers revealed broad endothermic peaks at 25.6, 9.4, 59, and 72.6 °C, respectively, for **CEPU1** to **CEPU4**, corresponding to melting of the hard regions, in the regime between −80 and 200 °C.

**Figure 2:**
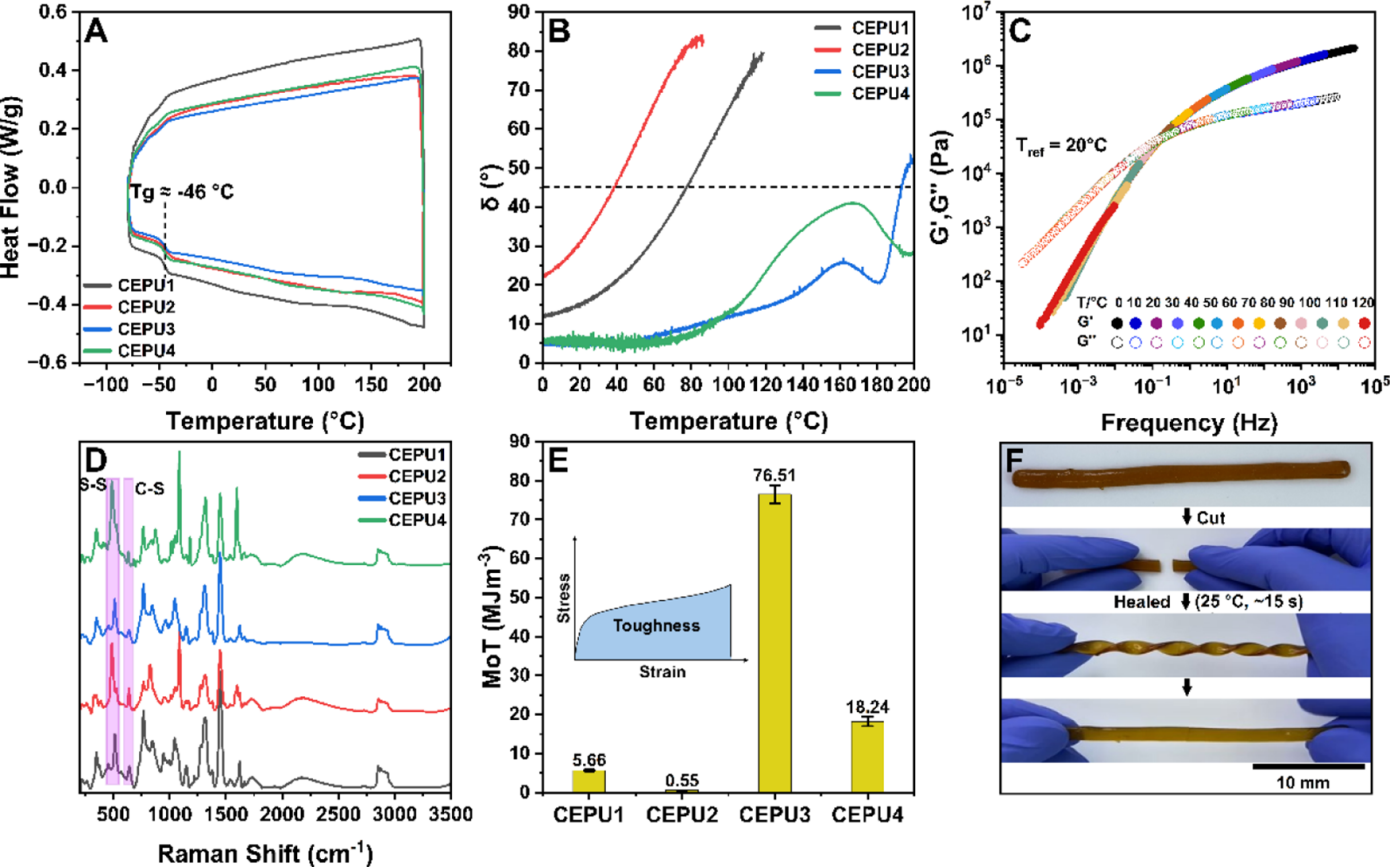
(A) DSC thermograms of the first heating–cooling cycle of **CEPU1**–**CEPU4** from −80 to 200 °C at a heating rate of 10 °C min^–1^; (B) rheological behavior (temperature sweep analysis) of **CEPU1**–**CEPU4** over a temperature range of 0–200 °C, using a normal force of 1 N and a frequency of 1 Hz, phase angle (δ) against temperature, where the dashed line represents phase transition from elastic solid to viscous; (C) master curve for **CEPU1** with a reference temperature (*T*_ref_) of 20 °C, where the rheological master curve was obtained by shifting the frequency sweep curves of different temperatures horizontally (α_T_) without shifting in the vertical direction; (D) Raman spectra of **CEPU1**–**CEPU4**; (E) toughness of CEPUs, calculated by integrating the area under the stress–strain curves. The error shown is the standard deviation between the three repeats for each sample. (F) Images of a twisted **CEPU1** strip film (thickness: 1 mm) after rapid healing at 25 °C for 15 s.

The dynamic viscoelasticity (rheological) measurements, including temperature dependencies of the storage modulus (*G*′), loss modulus (*G*″), and phase angle (δ) in the range of 0–200 °C, as well as small-amplitude oscillatory shear (SAOS) and time–temperature superposition (TTS) experiments, were investigated to acquire a comprehensive insight into the dynamic viscoelastic nature of the CEPU materials in their bulk states (static mode), see Figure 2B,C and Figures S45–S49. Low-temperature phase transitions were observed for **CEPU1** and **CEPU2**, attributed to the weakly packed hard segments and the increase in the polymer chain segmental motions. Extended rubbery behavior up to 100 °C was evident for **CEPU3** and **CEPU4**, and the storage modulus values remained essentially constant corresponding to the strong association between polymer chains because of the abundance of hydrogen bonding interactions via urea and urethane units. The phase angle curves demonstrate broad relaxations with increasing temperature as a result of the dissociation of the hydrogen bonds between the urea and urethane units. A crossover between *G*′ and *G*″ was observed at 152 °C in **CEPU3** and was attributed to the transition from an elastic solid to a viscous material.

In order to better understand the polymer chain relaxations and the linear viscoelastic behaviors, TTS experiments were conducted and master curves were plotted over a wide range of frequencies at a reference temperature (*T*_ref_) of 20 °C. It was observed that the curves can be divided into two main regimes with increasing frequency related to the crossover point between *G*′ and *G*″. The modulus for the rubber plateau, where (*G*′ > *G*″), is similar in **CEPU1** and **CEPU2** being observed at high frequencies. At the low frequency regime, all of the noncovalent and dynamic interactions were fully disrupted within the polymeric chains and viscous flow behavior was evident. **CEPU3** and **CEPU4** demonstrated a broad storage modulus and an elastic plateau over high frequencies with slight failure of TTS in **CEPU4** because of incomplete chain relaxation in the polymer networks. More importantly, the phase angle curves are always lower than 20° up to frequencies of 10^–7^ and 10^–9^ Hz of **CEPU3** and **CEPU4**, respectively. This observation confirms that the polymers dissipated minimal energy throughout the relaxing process and a wide temperature damping stability. To highlight these properties, a direct comparison between CEPU elastomers was established and showed that **CEPU3** and **CEPU4** exhibit longer chain relaxation times than **CEPU1** and **CEPU2** resulting from the increasing amount of hydrogen bonding that is expected from the urea units within the polymeric networks and the density of the hard segments.^55^ These findings suggest that all CEPU elastomers demonstrate elastic behavior at room temperature corresponding to the self-assembly between the polymer chains via the hydrogen bonding units (i.e., urethane and urea).^29^

The Raman spectrum of the CEPU elastomers confirmed the presence of (S–S) and (C–S) vibrations in their backbone structures as shown in Figure 2D, with a single band at ca. 513 cm^–1^ (**CEPU1** and **CEPU3**) and at ca. 486 cm^–1^ (**CEPU2** and **CEPU4**) assigned to the disulfide vibrations. Absorption bands corresponding to the C–S were observed at ca. 642 cm^–1^ (**CEPU1** and **CEPU3**) and at ca. 635 cm^–1^ (**CEPU2** and **CEPU4**).

To assess the stress–strain relationships of the resulting CEPU elastomers, the basic mechanical behaviors were investigated by uniaxial tensile strength tests (ISO 527-3) with a speed of 10 mm min^–1^ at room temperature. The representative tensile stress–strain curves, the determination of Young’s modulus (YM), the ultimate tensile strength (UTS), the elongation at break (EB), and the modulus of toughness (MoT) are presented for **CEPU1**–**CEPU4** in Figures S50 and S51. The disulfide chain-extender units and the polymerization process play a significant role in the resulting mechanical properties of the CEPU elastomers. The tensile curves of **CEPU1** and **CEPU2** exhibit extremely low UTS and possess a yield point with stress declination after yielding while being highly stretchable with maximum elongations >1600% and >1400%, respectively. The increase in the strain softening and ductility was attributed to the nonhomogeneous distribution of the hard segment aggregation, the breakage of dynamic disulfide bonds, and the weak hydrogen bonding via urethane units, all leading to polydisperse phase separation enhancing the stretchability and network relaxation. In contrast, the YM and UTS of **CEPU3** and **CEPU4** were higher and the stress–strain curves revealed stronger elastomers without yielding at low strains (i.e., tensile stress increases linearly with strain) while the degree of extension gradually decreases. The mechanical properties could stem from the uniform phase separation because of the synergistic effect of the strong hydrogen bonds via urea groups and the dynamic exchange of disulfide units during the deformation in the polymeric networks.

The relationship between the dynamic networks and the resultant mechanical properties of the CEPU elastomers was further investigated through tensile analysis of healed samples to evaluate the healing efficiency with further visual insight into the self-healing processes gained by conducting variable-temperature optical microscopy, see Figure 2F, Figures S52–S56, and Videos S1–S6. The self-healing efficiency of the polymer is defined as the ratio of the recovered YM, UTS, EB, and MoT values of the healed sample relative to the corresponding values of the pristine material.^56^ It is important to note that the self-healing efficiency can be affected by various factors such as the hard segment packing, density of the hydrogen bonding interactions, the reversible chemistry of disulfide units, and the motions of the polymer chains that are necessary to ensure activity of dynamic hard segments.^57,58^ **CEPU1** and **CEPU2** showed excellent self-healing behaviors, and the gap between the two parts of the damaged sample progressively disappeared with complete closure of the damage area achieved, reaching YM of 97% and 92% of their pristine values, respectively, and full recovery of UTS, after 30 min at 40 °C. The movement of molecular chains in the soft segment in **CEPU3** and **CEPU4** could be restricted as a result of strong association between the polymer chains via urea units, which may hinder the reconstruction of dynamic disulfide bonds at low temperature. This could be the reason for the increased healing temperature required (140 °C), and these elastomers exhibited YM of 99% and 69% of their pristine values and UTS of 99% and 69%, respectively, after 4 h. In summary, since the soft segments are identical in all of these CEPUs, the nature of the hard-phase structures and their distribution within the polymer are vital factors with respect to the self-healing performances of these elastomers.

The microstructure of the CEPUs was investigated at room temperature using small-angle X-ray scattering (SAXS) and wide-angle X-ray scattering (WAXS). The changes in the internal structure of the CEPU elastomers during tensile deformation (maximum displacement was 1400% at 100% intervals) were demonstrated by *in situ* SAXS measurements to understand the effect of phase separation on their mechanical characteristics such as elasticity, damage tolerance, and energy dissipation. Furthermore, SAXS profiles of **CEPU3** and **CEPU4** were investigated to examine the self-recoverability behavior when the samples were stretched to different strains. All the CEPU elastomers exhibit clear broad Bragg scattering peaks at 25 °C corresponding to microphase-separated segments arising from the immiscibility between the hard domains (hydrogen bonds and disulfide units) and the soft poly­(butadiene) domains, with scattering vectors *q*_peak_ of 0.88, 1.13, 1.03, and 1.00 nm^–1^, which correspond to length scales (*d*-spacings) of 7.13, 5.55, 6.09, and 6.28 nm for **CEPU1** to **CEPU4**, respectively, see Figures S57–S60. The WAXS patterns presented two distinct peaks, a sharp peak at 3.62 nm^–1^ (*d*-spacing of 1.73 nm) and a broad diffraction peak at approximately 12.50 nm^–1^ (*d*-spacing of 0.50 nm), which are associated with the ordered structure and hydrogen bonding of urethane–urea residues,^59,60^ respectively. Variable-temperature (VT) SAXS and WAXS experiments were investigated on thin-film samples of the CEPU elastomers at 25 °C intervals from 25 to 250 °C to assess thermal susceptibility on the bulk morphology of the CEPUs, Figures S61 and S62. The broad Bragg peaks were used to evaluate the intensity changes as a function of temperature. Generally, the diffraction peaks in the SAXS spectra shift to lower *q* values with increasing temperature ultimately resulting in the loss of the microphase-separated morphologies. Similar behavior in VT-WAXS was observed, where the *q*_max_ of the scattering peak shifted to lower values as result of the changes in the polymer structure via disassociation between the chains.

One-dimensional (1D) intensity profiles, azimuthal intensity profiles, and 2D patterns *in situ* SAXS were acquired to demonstrate the internal structural changes and the evolution of the phase separation morphologies when the CEPU elastomers changed from static to dynamic mode as a function of strain. During the elongation process, the intensity of the diffraction peaks decreased significantly for all the elastomers, whereas the positions of the maxima of the peaks did not change, but the peaks became broader, indicating destruction of the microphase-separated structure through stretching, see Figure S63. The scattering circles in 2D-SAXS scattering patterns were evident as a ring configuration in the initial state as a result of the phase separation phenomena and the isotropic distribution of scattering signals, indicating that the hard segments and the surrounding soft segments were oriented randomly.

2D-SAXS scattering profiles of **CEPU1** and **CEPU2** presented fully isotropic scattering patterns for the covered *q*-range in the initial state and up to 1400% stretching, see Figures S64–S71. The 2D-SAXS patterns of **CEPU3** and **CEPU4** during stretching at different strains ranging from 0 to 1400% (see Figure 3A,B and Figures S72–S84) revealed increased ordering at elevated strains. The scattering pattern for **CEPU3** and **CEPU4** presented more features and complexity when compared to **CEPU1** and **CEPU2**, especially the elliptical scattering at the lower stains. It was noted that the equator of the ellipse of **CEPU3**, see Figure 3AI, at zero strain is tilted by ca. 45°, confirming a macroscopically isotropic nanostructure with no preferential domain orientation. The ellipse tilts and the equator align horizontally with larger strain. At low to moderate strains between 0 and 100%, the 2D patterns of the **CEPU3** elastomer transition from a ring configuration to a weakly elliptical symmetry, with developing intensity concentration in the equatorial direction, see Figure 3AI,II. The meridional 1D profile presented a systematic shift the *q*_max_ of the scattering peak to lower values, reflecting an increase in the interdomain spacing along the stretch direction. At strains ranging from 100 to 300%, the 2D-SAXS profiles for **CEPU3** demonstrated mechanistic behavior corresponding to the start of active disulfide chain exchange. The polymer chain architectures become taut under stress, which leads to activating disulfide chain exchange, allowing structural rearrangement of the covalent network without macroscopic bond rupture. Similar features are found when measuring out of the midpoint and edge-point of the **CEPU3** and **CEPU4** elastomers, which have been stretched to different strains (from 0% to 500% at a 100% interval). 1D- and 2D-SAXS profiles during cyclic tensile tests (from 0% to 100% at a 20% interval) and then retraction for **CEPU3** and **CEPU4** elastomers were examined to investigate the deformation recovery and recoverable of the microphase-separated behavior, see Figure 3C and Figures S85–S89. At 0% strain, the 1D-SAXS profiles for **CEPU3** and **CEPU4** show *q*_max_ values of 1.03 and 1.01 nm^–1^, which are attributed to length scales (*d*-spacings) of 6.09 and 6.21 nm, respectively. As deformation continues, an anisotropic state was observed in 2D-SAXS with a broader shape, with modified scattering values *q*_max_ of 0.95 and 0.80 nm^–1^, which correspond to length scales (*d*-spacings) of 7.85 and 6.61 nm for **CEPU3** and **CEPU4**, respectively. After releasing the strain, the polymer samples slowly relaxed, and the recovery state patterns of the elastomers were observed suggesting that the polymers possess self-recoverability. These behaviors represent the nanoscale structural characteristics of two cooperative self-recovery mechanisms: hydrogen bonding reassembly of phase-separated hard domains, which are dynamically driven to change their equilibrium-aggregated arrangement after stress removal, and disulfide chain exchange, which equilibrates the network structure during recovery, reducing residual chain orientation and restoring chain configurations.

**Figure 3:**
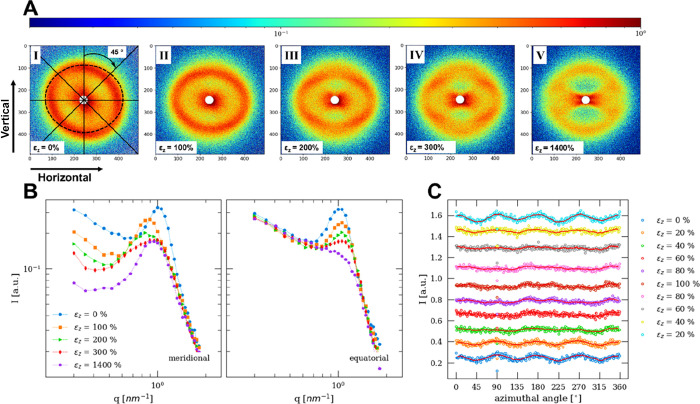
(A) Evolution of 2D-SAXS patterns for **CEPU3** measured as a function of strain during selected elongation from 0% to 1400% at 100% intervals (selected), where the relaxed state refers to images taken 10 min after strain measurements were taken; (B) 1D meridional and equatorial SAXS profiles of **CEPU3** at selected strains; and (C) azimuthal angle profiles of the 2D pattern for **CEPU3** as a function of strain during cycle elongation from 0% to 100% to 20% at 20% intervals.

Consecutive compression cyclic tensile tests of **CEPU3** and **CEPU4** were demonstrated at 25 °C where the samples were deformed to a strain of 20% to investigate their elastic recovery during 10 cycles of deformation, see Figure 4A and Figure S90. Hysteresis compression loading–unloading tensile tests of **CEPU3** and **CEPU4** at various deformations (20%, 40%, 60%, and 80%) were also performed to evaluate the deformation recovery, the recoverable energy of dissipation, and to assess the relaxation time required for the recovery of the original tensile behavior. The energy dissipated was calculated from the area of the hysteresis loop between the loading and unloading curves per unit volume for the cycle. Hysteresis behavior was demonstrated as the ratio of the energy loss and the energy required to deform the sample, i.e., the area under the loading–unloading curve.^61^ Clear hysteresis loops were observed in each cycle through 10 continuous loading–unloading cycles at a fixed strain of 20% for **CEPU3** and **CEPU4**, indicating significant energy absorption (energy dissipation) during repeated cycles. The unloading paths do not fully return to the origin, suggesting incomplete strain recovery within the time scale of the test. Overall, the loading curves show no obvious progressive reduction across the cycles, while the loop area remains appreciable, demonstrating repeatable dissipative behavior under cyclic compression. The stress values at 20% compression decreased as the number of cycles increased, from 34.5 to 27.0 MPa (21.7% reduction over 10 cycles). This decrease suggests that a longer relaxation time is needed to reconfigure the hard domains. **CEPU3** and **CEPU4** elastomers exhibited excellent durability, energy dissipation capability, and fatigue resistance because of the dynamic behavior of the hydrogen bonds and disulfide linkages during the deformation process. Furthermore, Figure 4C,D and Figure S91–S93 display quantified results for the dissipated energies and elastic recovery (ER %) ratios^62^ of **CEPU3**. The area of the hysteresis loop was 7880 J m^–3^ at a fixed strain level of 20%, and the ER % was 67 ± 8.1, indicating that the dynamic system was fractured to dissipate energy during the compression process. At a deformation of 80%, the ER % reached 77 ± 0.3 with dissipated energies of 235,000 J m^–3^ (increased by a factor of 30) while the efficiency of energy dissipation was increased to 54%. Therefore, it can be concluded that the rapid reversible association/dissociation of **CEPU3** enables the energy-dissipative nature of these elastomers allowing them to achieve an impact-protective capability via their dynamic networks.

**Figure 4:**
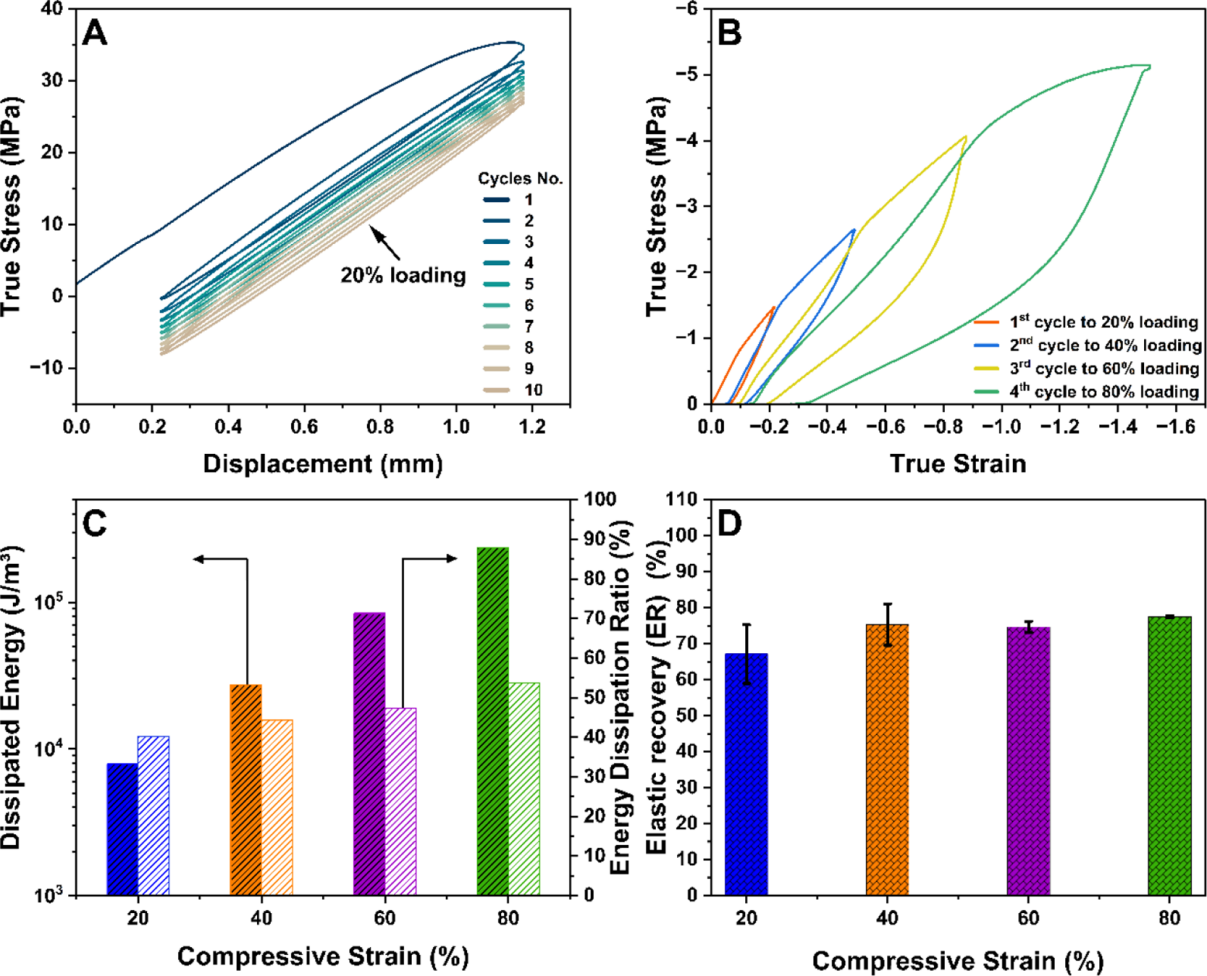
(A) Consecutive compression cyclic tensile tests of **CEPU3** at 25 °C without relaxation, where specimens were compressed 10 times at 20% deformation; (B) single cyclic compression tensile tests of **CEPU3** at 25 °C under various deformations of 20%, 40%, 60%, and 80% in successive compression; (C) compressive strain-dependent energy dissipation ratios and dissipated energies of **CEPU3**; and (D) comparison of elastic recovery of **CEPU3**.

Compression and tension true stress–strain experiments were investigated to further assess the mechanical behaviors of the CEPU elastomers, with a speed of 10 mm min^–1^ at room temperature. Digital image correlation (DIC) was used for direct visualization of the elastomer deformation during the test. A split-Hopkinson pressure bar (SHPB) system and a hydraulic machine were utilized to further evaluate the dynamic impact properties of **CEPU3** and **CEPU4** elastomers at different medium- and high-strain-rate compressive behaviors. Building on the cyclic loading results, load–relax–reload tests were conducted to further assess recovery behavior, as cyclic loading alone may not provide sufficient time for full strain recovery, see Figure 5A,B. When loaded to a true strain of 20%, the **CEPU3** specimen recovered to its original dimensions after unloading and a recovery period of approximately 1 min. The subsequent reload curve almost fully overlapped with the first loading response. For the higher strain level of 40%, recovery was observed after a longer recovery period of approximately 6 min. The reloading response again revealed near-complete overlap with the initial loading curve. These results indicate that the mechanical performance is largely preserved after unloading and recovery, demonstrating the efficient self-recovery capability of the material within the investigated strain range and time scales. Relaxation time and elastic recovery (ER %) were calculated as a function of strain for **CEPU3** and **CEPU4** at two deformations of 20% and 40%; at this level of compression, the elastomers would not incur permanent damage and the dynamic behavior in the system could operate effectively. The elastic recovery of **CEPU3** was recovered to 91% and 99% after relaxing for 1 and 6 min at 20% stretching, respectively. The hysteresis loops and stress values recovered approximately to the initial behavior, suggesting excellent elastic restorability. **CEPU4** exhibited a similar trend of elastic recovery and relaxation time at 20% deformation, while at a fixed strain level of 40%, the elastomer recovered the original hysteresis loop after standing for 3 min. Self-recovery behavior after deformation is fundamentally directed by the noncovalent networks’ reformation following load removal; the elastic recovery in supramolecular elastomers based on hydrogen bonds is a time-dependent process that is affected by chain mobility as well as the density of reversible interactions.^16,63^ Research studies of supramolecular elastomers under cyclic deformation have shown that self-recovery requires time scales ranging from a few minutes to several hours, based on the composition and architecture of the dynamic network.^63,64^ Multifunctional supramolecular polyurethanes^65^ have been reported by Wen and co-workers; with shape memory capability after deformation, the polymer could recover its original shape after molecular relaxation and exposure to thermal external stimuli at 70 °C. The combination between noncovalent interactions (hydrogen bonds) and dynamic covalent disulfide bonds in the polymer networks has provided effective reversible cross-linking and recoverability performance via dynamic dissociation and reassociation during stretching. For example, Zhang and co-workers have developed elastic polyurethanes^66^ that after extension and a 5 min waiting period were able to revert to their initial states. With respect to such materials, the self-recoverability of **CEPU3** and **CEPU4** at room temperature was comparable under less demanding conditions.

**Figure 5:**
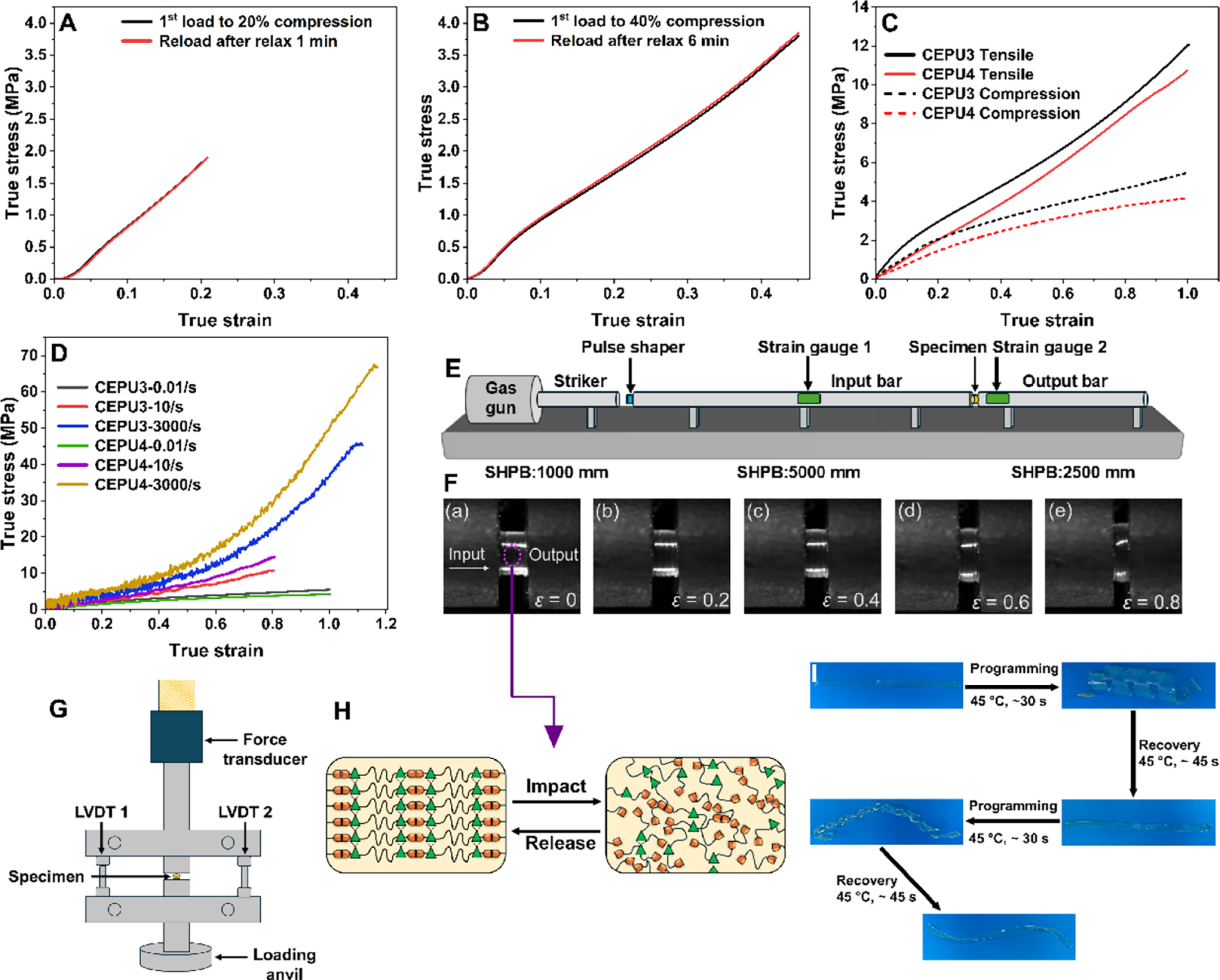
Compression load–relax–reload for **CEPU3** to (A) 20% true strain; (B) 40% true strain, at 25 °C, where after relaxing, the cycle curve was overlapped with the original cycle; (C) quasi-static tensile and compression tests for **CEPU3** and **CEPU4**; (D) rate-dependent compression behaviors for **CEPU3** and **CEPU4**; (E) experimental setup for the SHPB test; (F) images of **CEPU3** specimens during SHPB experiments recorded by a high-speed camera at ∼3000 s^–1^; (G) experimental setup for the hydraulic machine; (H) illustration of impact resistance mechanism of **CEPU3** and **CEPU4**; and (I) photographs illustrating the shape memory performance of **CEPU3**.

The quasi-static true stress–true strain responses of **CEPU3** and **CEPU4** under tension and compression are shown in Figure 5C. During tensile loading, no obvious grip slip or strain localization was observed, and this was further validated using DIC, as shown in Figures S94–S98. The tensile true stress–true strain curves were derived directly from the measured load–displacement data, enabling a consistent, direct comparison with the compression results. All the specimens recovered to their original gauge length after unloading, as indicated by the gauge marks made before testing, although the recovery occurred over different time scales. The results also reveal a clear tension–compression asymmetry: for the same material, higher stress levels were observed under tensile loading than under compression. This behavior is likely to be associated with stretching of the hard segments, which resist deformation more strongly in tension. Similar responses have been reported previously for TPUs.^67,68^ A comparison of these two elastomers reveals that **CEPU3** consistently exhibits higher strength, which can be attributed to differences in the polymer composition. Figure 5D shows the rate-dependent compression responses of **CEPU3** and **CEPU4**. Both materials exhibit pronounced rate sensitivity, with stress increasing markedly as strain rate rises, particularly at larger strains. Notably, while **CEPU3** is stronger under quasi-static loading, **CEPU4** exhibits greater rate dependence at medium (∼10 s^–1^) and high (∼3000 s^–1^) strain rates, resulting in higher stresses under these faster loadings. This difference is likely to be related to the distinct chemistry and microstructural design of the soft/hard segments in the two CEPU elastomers. Notably, *in situ* high-speed photography was used during the SHPB tests to directly observe the deformation process, see Figure 5E–G. No apparent failure was observed for either material. After testing, the specimens were examined and were found to have fully recovered their dimensions, suggesting that hydrogen bonds and disulfide units with significant energy dissipation, see Figure 5H, can provide self-reinforcing and toughening properties to materials. The temperature-responsive shape-fixing and recovery performance of **CEPU3** was investigated, see Figure 5I, Figure S99, and Video S7. According to the findings from DSC and rheological characterization, **CEPU3** has *T*_g_ at ca. −46 °C and an onset of hard segment melting at ca. 41.5 °C, the exploitation of which can be used as a fixation temperature.^69,70^ A flat rectangular strip (145 × 65 × 0.55 mm) was shape-fixed by wrapping around a cylindrical glass rod at 45 °C for ∼30 s in a hot water bath and then cooled at 15 °C for ∼30 s to adopt the first temporary shape of a helical strip. Subsequently, the helix was heated again to 45 °C, whereby near-complete recovery of its original shape was achieved within ∼45 s, indicating excellent editable shape memory behavior.

Finally, the falling steel ball test was used to evaluate the impact-protective performance of the CEPU elastomers as impact-resistant coatings of fragile surfaces. A glass slide with a thickness of 1 mm was covered with the CEPU elastomer of the same dimensions and thickness (75 × 25 × 1 mm); then, three different steel balls weighing 50, 100, and 150 g were dropped sequentially onto the covered sample from a height of 1.20 m to mimic the force of everyday impact processes, see Figure 6. In addition, commercial polymers such as HDPE, PP, and Nylon-6,6 were used as impact-protective materials by way of comparison against the CEPUs. The unprotected glass sample shattered instantly while the sample protected with the CEPUs remained intact upon impact by all of the steel balls. In stark contrast, under identical test conditions, the commercial materials failed to protect the glass slide. The CEPU elastomers thus possess the ability to protect fragile surfaces against impact when compared to commercially available materials, see Videos S8 and S9.

**Figure 6:**
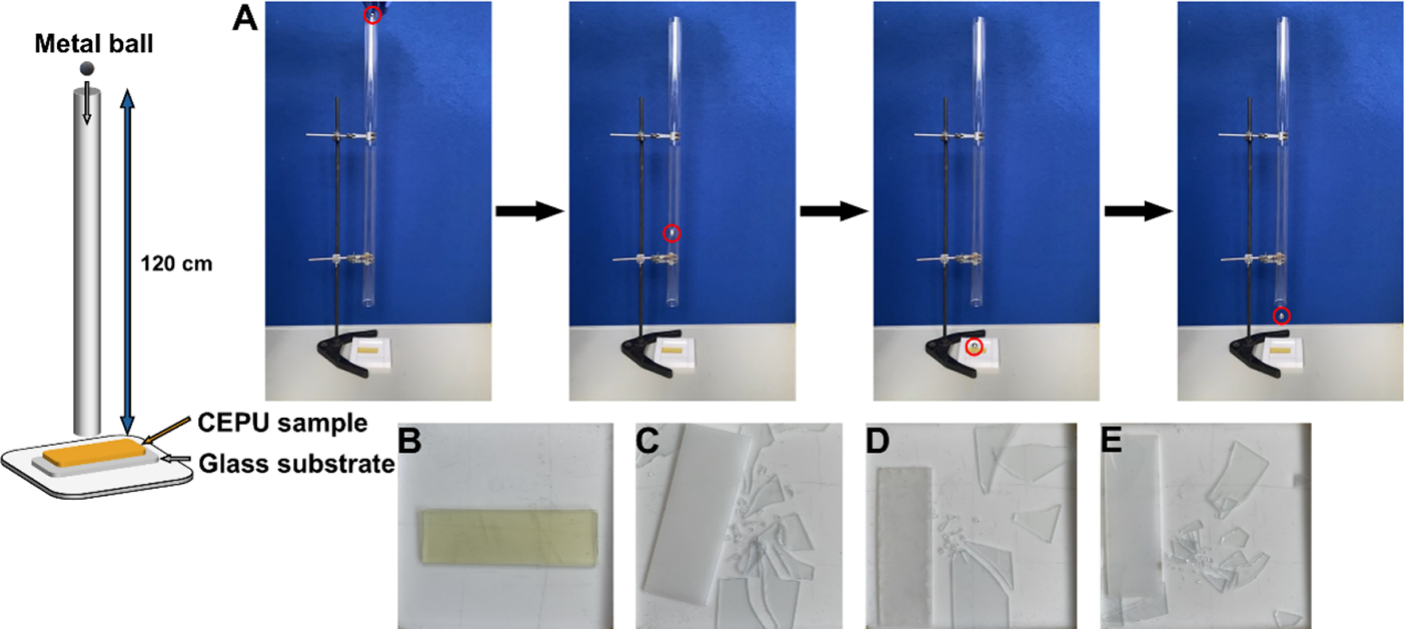
(A) Schematic illustration of the equipment and the setup used for the falling steel ball test (50, 100, and 150 g), where photographs depict a 50 g steel ball falling onto a **CEPU3**-coated glass slide. Photographs of the (B) **CEPU3**, (C) HDPE, (D) PP, and (E) Nylon-6,6 after the impact of a 50 g steel ball from 1.20 m.

## Conclusions

This study reports the development of chain-extended supramolecular polyurethane and poly­(urethane–urea) elastomers that exhibit self-recovery behavior, efficient energy dissipation, and autonomous healing properties, paving the way for the impact-resistant systems. These elastomers were designed to integrate hydrogen bonding interactions and disulfide chain-extender units to construct dynamic networks. The exceptional self-recoverability performance (99%) of the optimum elastomer was exhibited after relaxing the material for 6 min at 40% compression. The elastic recovery ratios have been calculated during loading–unloading compression cycles experiments, and the ER % reached 77 ± 0.3% at a deformation of 80%, which was increased by a factor of 30 of that at a deformation of 20%. The changes in the internal structure of CEPU elastomers were studied by *in situ* SAXS stretching measurements revealing evolution of the phase separation morphologies during deformation from a static to dynamic state. This study highlights a unique and effective strategy for the exploitation of dynamic covalent networks in combination with noncovalent interactions to create dual dynamic networks capable of excellent repair and energy dissipation, key attributes in the development of new materials for deployment in applications such as personal protective equipment.

## Supplementary Material



























## Notes

Crystallographic data for analogue **1** (CIF)
Crystallographic data for analogue **3** (CIF)
Crystallographic data for analogue **4** (CIF)
Detailed synthetic protocols of the small-molecule analogues and CEPUs; single-crystal XRD and MS for small-molecule analogues; characterization data for the CEPUs, including NMR, FT-IR, GPC, TGA, DSC, tensile strength, SAXS and WAXS, rheological experiments, and additional AFM images (PDF)
(Video S1) Self-healing process of a **CEPU1** film (MP4)
(Video S2) Self-healing process of a **CEPU2** film (MP4)
(Video S3) Self-healing process of a **CEPU3** film (MP4)
(Video S4) Self-healing process of a **CEPU4** film (MP4)
(Video S5) Self-healing behavior of a **CEPU1** film (MP4)
(Video S1) Self-healing behavior of a **CEPU2** film (MP4)
(Video S7) Temperature-responsive shape memory of **CEPU3** (MP4)
(Video S8) Dropping ball impact test using **CEPU3** (MP4)
(Video S8) Dropping ball impact test using **CEPU4** (MP4)
