# Supplementary material for: Self-Recoverable, Energy-Dissipating, and Healable Chain-Extended Supramolecular Polyurethanes and Poly(urethane–urea)s for Impact-Resistant Systems
Source: ACS Appl Mater Interfaces. 2026 Jul 1;18(27):38032–44. doi: 10.1021/acsami.6c06182 (PMC13383276; doi:10.1021/acsami.6c06182)
Supplement: Supplementary file 4 [file am6c06182_si_004.pdf]

**Supporting Information**

# Self-recoverable, energy dissipating, and healable chain extended supramolecular polyurethanes and poly(urethane-urea)s for impact resistant systems

Alarqam Z. Tareq,[a,b] Matthew Hyder,[a] Peihao Song,[c] Georgios Kalimeris[c], Thomas Zinn,[d] James E. Hallett[a], Ann M. Chippindale,[a] Clive R. Siviour,[c] and Wayne Hayes[a]*

[a] Department of Chemistry, University of Reading, Whiteknights, Reading, RG6 6DX, UK.
[b] Department of Chemistry, Faculty of Science, University of Zakho, Duhok 42001, Iraq.
[c] Department of Engineering Science, University of Oxford, Parks Road, OX1 3PJ, UK.
[d] Diamond Light Source, Diamond Light Source Ltd, Harwell Science & Innovation Campus, Didcot, OX11 0DE, UK.

*Corresponding author e-mail: w.c.hayes@reading.ac.uk

## Contents

**General synthetic protocol for CEPUs.** .......... 8

**Figure S1**: The synthetic route used to synthesis **CEPU1-CEPU4**. .......... 8

**Synthesis of CEPU1** .......... 9

**Synthesis of CEPU2** .......... 9

**Synthesis of CEPU3** .......... 10

**Synthesis of CEPU4** .......... 11

**Figure S2.** Representative homogeneous cast films (10 cm × 10 cm × 0.5 mm) of (A) **CEPU1**, (B) **CEPU2**, (C) **CEPU3**, and (D) **CEPU4** in aluminium moulds. .......... 12

**Table S1**. The key $^{1}$H and $^{13}$C NMR spectroscopic resonances for **CEPU1-CEPU4** in THF-d8. .......... 12

**Figure S3**. $^{1}$H NMR spectrum of **CEPU1** (400 MHz, THF-$d_8$, at 25 °C). .......... 13

**Figure S4**. $^{13}$C NMR spectrum of **CEPU1** (100 MHz THF-$d_8$, at 25 °C). .......... 13

**Figure S5**. $^{1}$H NMR spectrum of **CEPU2** (400 MHz, THF-$d_8$, at 25 °C). .......... 14

**Figure S6**. $^{13}$C NMR spectrum of **CEPU2** (100 MHz THF-$d_8$, at 25 °C). .......... 14

**Figure S7**. $^{1}$H NMR spectrum of **CEPU3** (400 MHz, THF-$d_8$, at 25 °C). .......... 15

**Figure S8**. $^{13}$C NMR spectrum of **CEPU3** (100 MHz THF-$d_8$, at 25 °C). .......... 15

**Figure S9**. $^{1}$H NMR spectrum of **CEPU4** (400 MHz, THF-$d_8$, at 25 °C). .......... 16

**Figure S10**. $^{13}$C NMR spectrum of **CEPU4** (100 MHz THF-$d_8$, at 25 °C). .......... 16

**Figure S11**. The FTIR spectrum of **CEPU1-CEPU4**. .......... 17

**Figure S12**. GPC eluograms of **CEPU1** (A), **CEPU2** (B), **CEPU3** (C), and **CEPU4** (D), in THF. .......... 18

**Figure S13**. AFM tapping mode phase images of **CEPU1** (**A**, **B**) and **CEPU2** (**C**, **D**) for two different scale bars 1 µm and 500 nm. All polymer samples are prepared by drop casting from THF on the mica disc. .......... 19

**Figure S14**. AFM tapping mode phase images of **CEPU3** (**A**, **B**) and **CEPU4** (**C**, **D**) for two different scale bars 1 µm and 500 nm. All polymer samples are prepared by drop casting from THF on the mica disc. .......... 20

**General Synthesis of disulfide small molecule analogues** .......... 21

**Synthesis of 1,1'-(disulfanediylbis(ethane-2,1-diyl))bis(3-phenylurea) (1).** .......... 21

**Synthesis of disulfanediylbis(4,1-phenylene) bis(phenylcarbamate) (2).** .......... 21

**Synthesis of 1,1'-(disulfanediylbis(ethane-2,1-diyl))bis(3-phenylurea) (3).** .......... 22

**Synthesis 1,1'-(disulfanediylbis(4,1-phenylene))bis(3-phenylurea) (4).** .......... 22

**Protocol for solution state $^{1}$H NMR kinetic study of chains exchange disulfide small molecule analogues** .......... 23

**Figure S15**. $^{1}$H NMR spectrum of analogues **1** (100 MHz, DMSO-$d_6$, at 25 °C). .......... 23

**Figure S16**. $^{13}$C NMR spectrum of **analogue 1** (400 MHz, DMSO-$d_6$, at 25 °C). .......... 24

**Figure S17**. 2D COSY ($^{1}$H-$^{1}$H) NMR spectrum of **analogue 1** (400 MHz, DMSO-$d_6$, at 25 °C). .......... 24

**Figure S18**. $^{1}$H NMR spectrum of **analogue 2** (400 MHz, DMSO-$d_6$, at 25 °C). .......... 25

**Figure S19**. $^{13}$C NMR spectrum of **analogue 2** (100 MHz, DMSO-$d_6$, at 25 °C). .......... 25

**Figure S20**. 2D COSY ($^{1}$H-$^{1}$H) NMR spectrum of **analogue 2** (400 MHz, DMSO-$d_6$, at 25 °C). .......... 26

**Figure S21**. $^{1}$H NMR spectrum of **analogue 3** (400 MHz, DMSO-$d_6$, at 25 °C). .......... 26

**Figure S22**. $^{13}$C NMR spectrum of **analogue 3** (100 MHz, DMSO-$d_6$, at 25 °C). .......... 27

**Figure S23**. 2D COSY ($^{1}$H-$^{1}$H) NMR spectrum of **analogue 3** (400 MHz, DMSO-$d_6$, at 25 °C). .......... 27

**Figure S24**. $^{1}$H NMR spectrum of **analogue 4** (400 MHz, DMSO-$d_6$, at 25 °C). .......... 28

**Figure S25**. $^{13}$C NMR spectrum of **analogue 4** (100 MHz, DMSO-$d_6$, at 25 °C). .......... 28

**Figure S26**. 2D COSY ($^{1}$H-$^{1}$H) NMR spectrum of **analogue 4** (400 MHz, DMSO-$d_6$, at 25 °C). .......... 29

**Figure S27**. The asymmetric unit of **analogue 1** was determined from single-crystal X-ray diffraction analysis. One molecule of **analogue 1** (Symmetry Code (i): 2 – *x*, 2 – *y*, *z*). There are two molecules in the unit cell. Thermal ellipsoids are drawn at 50% probability. .......... 30

**Table S2**. Crystallographic details for **analogue 1** .......... 30

**Table S3**. Selected bond lengths (Å) and angles (°) in **analogue 1** .......... 31

**Table S4**. Hydrogen-bond and close-contact geometry (Å, °) in **analogue 1**.............. 31

**Figure S28**. Packing of the molecules of **analogue 1**. view along the *c* axis showing the stacking. ........................................................................................................................ 32

**Figure S29**. Packing of the molecules of **analogue 1**, view along the *a* axis showing the N-H…O hydrogen-bonding interactions (pale-blue lines, N(7) – H(71)…O(6)$^{ii}$, 2.953(2) Å) between adjacent molecules to form layers lying in the *bc* plane. Symmetry Code: (ii) *x* , *y*, *z* + 1........................................................................................................................ 33

**Figure S30**. The asymmetric unit of **analogue 3** was determined from single-crystal X-ray diffraction analysis. There are two molecules in the asymmetric unit and eight in the unit cell. Thermal ellipsoids are drawn at 50% probability............................................. 34

**Table S5**. Crystallographic details for **analogue 3**......................................................... 34

**Table S6**. Selected bond lengths (Å) and angles (°) in **analogue 3**.............................. 35

**Table S7**. Hydrogen-bond and close-contact geometry (Å, °) in **analogue 3**.............. 37

**Figure S31**. Packing of the molecules of compound **analogue 3**. view along the *a* axis showing the bifurcated N-H…O hydrogen-bonding interactions (pale-blue lines) within and between the two distinct molecules in the asymmetric unit leading to chains running in the *c* direction.......................................................................................................... 38

**Figure S32**. The asymmetric unit of **analogue 4** was determined from single-crystal X-ray diffraction analysis. one molecule of **analogue 4** (Symmetry Code (i): –*x* + 1, *y*, – *z* + 3/2). There are four molecules in the unit cell. Thermal ellipsoids are drawn at 50% probability.......................................................................................................................... 38

**Table S8**. Crystallographic details for **analogue 4**......................................................... 39

**Table S9**. Selected bond lengths (Å) and angles (°) in **analogue 4**.............................. 39

**Table S10**. Hydrogen-bond and close-contact geometry (Å, °) in **analogue 4**. ........... 40

**Figure S33**. Packing of the molecules of compound **analogue 4**, view along the *a* axis showing the stacking of the molecules............................................................................ 41

**Figure S34**. Packing of the molecules of compound **analogue 4**, view along the *b* axis showing the bifurcated N-H…O hydrogen-bonding interactions (pale-blue lines) between adjacent molecules (N(6) – H(61)…O(8)$^{ii}$, 2.875(3) Å and N(9) – H(91)…O(8)$^{ii}$, 2.937(3) Å)........................................................................................................................ 41

**Figure S35**. $^{1}$H NMR spectra of (A) **analogue 4**, (B) **analogue 3** (C) mixture of **analogue 3** and **4** at room temperature, and (D,E) mixture of **analogue 3** and **4** after heating to 80 °C for 30, 60 and 120 minutes, respectively, in DMSO-$d_6$. ..................... 42

**Figure S36**. (A) Raman spectra of **CEPU4** as a function of strain, (B) ordered/disordered disulfide signals, and (C) load force development during stretching from 0% to a maximum of 100% at 10 % interval............................................................ 43

**Figure S37**. TGA thermogram of **CEPU1** at 10 °C min$^{-1}$ under nitrogen...................... 44

**Figure S38**. TGA thermogram of **CEPU2** at 10 °C min$^{-1}$ under nitrogen...................... 44

**Figure S39**. TGA thermogram of **CEPU3** at 10 °C min$^{-1}$ under nitrogen...................... 45

**Figure S40**. TGA thermogram of **CEPU4** at 10 °C $min^{-1}$ under nitrogen. 45

**Figure S41**. DSC thermogram of **CEPU1** shows the 1st, 2nd, and 3rd heating and cooling cycles from -80 °C to 200 °C at 10 °C $min^{-1}$, 1st heating after isotherm at -90 °C for 60 min, and 3rd heating up to 250 °C. 46

**Figure S42**. DSC thermogram of **CEPU2** shows the 1st, 2nd, and 3rd heating and cooling cycles from -80 °C to 200 °C at 10 °C $min^{-1}$, 1st heating after isotherm at -90 °C for 60 min, and 3rd heating up to 250 °C. 46

**Figure S43**. DSC thermogram of **CEPU3** shows the 1st, 2nd, and 3rd heating and cooling cycles from -80 °C to 200 °C at 10 °C $min^{-1}$, 1st heating after isotherm at -90 °C for 60 min, and 3rd heating up to 250 °C. 47

**Figure S44**. DSC thermogram of **CEPU4** shows the 1st, 2nd, and 3rd heating and cooling cycles from -80 °C to 200 °C at 10 °C $min^{-1}$, 1st heating after isotherm at -90 °C for 60 min, and 3rd heating up to 250 °C. 47

**Table S11**: GPC molecular weight, polydispersity data and thermal properties for **CEPU1-CEPU4**. 48

**Figure S45**. Temperature sweep analysis of **CEPU1** (A), **CEPU2** (B), **CEPU3** (C), and **CEPU4** (D), using a normal force of 1 N and a frequency of 1 Hz. 48

**Figure S46**. (A) Raw rheological data for **CEPU1**, (B) Master curves with a reference temperature ($T_{ref}$) of 20 °C, the rheological master curves were obtained by shifting the frequency sweep curves of different temperatures horizontally (αT) without shifting in the vertical. 49

**Figure S47**. (A) Raw rheological data for **CEPU2**, (B) Master curves with a reference temperature ($T_{ref}$) of 20 °C, the rheological master curves were obtained by shifting the frequency sweep curves of different temperatures horizontally (αT) without shifting in the vertical. 50

**Figure S48**. (A) Raw rheological data for **CEPU3**, (B) Master curves with a reference temperature ($T_{ref}$) of 20 °C, the rheological master curves were obtained by shifting the frequency sweep curves of different temperatures horizontally (αT) without shifting in the vertical. 51

**Figure S49**. (A) Raw rheological data for **CEPU4**, (B) Master curves with a reference temperature ($T_{ref}$) of 20 °C, the rheological master curves were obtained by shifting the frequency sweep curves of different temperatures horizontally (αT) without shifting in the vertical. 52

**Figure S50**. Tensile stress-strain curves of CEPUs, (A) **CEPU1** and **CEPU2**, (B) **CEPU3** and **CEPU4**. 53

**Figure S51**. Mechanical properties, Ultimate Tensile Strength (UTS), Young's modulus (YM), Modulus of toughness (MoT), and elongation at break (EB) for **CEPU1-CEPU4**. The error shown is the standard deviation (values shown are the averages of 3 repeat measurements for each sample). 53

**Figure S52**. Tensile stress-strain curves of the pristine and healed **CEPU1** (A), **CEPU2** (B), **CEPU3** (C), and **CEPU4** (D) at different healing times. 54

**Figure S53**. Healing efficiency of the YM (A), UTS (B), EB (C), and MoT (D) of the healed **CEPU1** at different healing times at 40 °C. The error shown is the standard deviation (values shown are the averages of 3 repeat measurements for each sample). ........ 55

**Figure S54**. Healing efficiency of the YM (A), UTS (B), EB (C), and MoT (D) of the healed **CEPU2** at different healing times at 40 °C. The error shown is the standard deviation (values shown are the averages of 3 repeat measurements for each sample). ........ 56

**Figure S55**. Healing efficiency of the YM (A), UTS (B), EB (C), and MoT (D) of the healed **CEPU3** at different healing times at 140 °C. The error shown is the standard deviation (values shown are the averages of 3 repeat measurements for each sample). ........ 57

**Figure S56**. Healing efficiency of the YM (A), UTS (B), EB (C), and MoT (D) of the healed **CEPU4** at different healing times at 140 °C. The error shown is the standard deviation (values shown are the averages of 3 repeat measurements for each sample). ........ 58

**Figure S57**. In-air setup of labSAXS with mounted micro mechanical tensile tester installed. ........ 58

**Figure S58**. (A) SAXS and (B) WAXS scattering patterns of **CEPU1**- **CEPU4** at 25 ◦C. ........ 59

**Figure S59**. 1D SAXS profiles of (A) **CEPU1**, (B) **CEPU2**, (C) **CEPU3**, and (D) **CEPU4** and corresponding fitlines at 25 °C. ........ 59

**Figure S60**. SAXS fitting residuals of (A) **CEPU1**, (B) **CEPU2**, (C) **CEPU3**, and (D) **CEPU4** at 25 °C. ........ 60

**Figure S61**. VT-SAXS profiles of (A) **CEPU1**, (B) **CEPU2**, (C) **CEPU3** and (D) **CEPU4** as a function of temperature, recorded at 25 °C intervals from 25 °C to 250 °C at a heating rate of 25 °C $min^{-1}$. ........ 61

**Figure S62**. VT-WAXS profiles of (A) **CEPU1**, (B) **CEPU2**, (C) **CEPU3** and (D) **CEPU4** as a function of temperature, recorded at 25 °C intervals from 25 °C to 250 °C at a heating rate of 25 °C $min^{-1}$. ........ 62

**Figure S63**. The evolution of 1D SAXS pattern as a function of strain from 0% to a maximum of 1400%, (A) **CEPU1**, (B) **CEPU2**, (C) **CEPU3**, and (D) **CEPU4**. The stretching direction is vertical. ........ 63

**Figure S64**. 2D SAXS pattern for the **CEPU1** elastomer: (A) at 0% strain and (B) at 1400% strain, respectively. ........ 64

**Figure S65**. Azimuthal profile for the **CEPU1** elastomer: (A) at 0% strain and (B) at 1400% strain, respectively. ........ 64

**Figure S66**. Intensity vs azimuthal angle of **CEPU1** elastomer at 0% strain (black open points) and 1400% strain (blue open symbols). The red line is smoothing of the data. ........ 65

**Figure S67**. Intensity vs q of the **CEPU1** elastomer 0% strain (black open symbols) and 1300% (black squares) strain. 65

**Figure S68**. 2D SAXS pattern for the **CEPU2** elastomer: (A) at 0% strain and (B) at 1300% strain, respectively. 66

The scattering is fully isotropic for the covered q-range (indicated by the red dashed circular line). While as before the scattering intensity decreases with increasing strain due to thinning of the sample the 2D scattering pattern develops an anisotropy in the equatorial plane, see (B) indicated by the two red circles. 66

**Figure S69**. Azimuthal profile for the **CEPU2** elastomer: (A) at 0% strain and (B) at 1300% strain, respectively. 66

**Figure S70**. Intensity vs azimuthal angle of **CEPU2** elastomer at 0% strain (black open points) and 1300% strain (blue open symbols). 67

**Figure S71**. Intensity vs q of the **CEPU2** elastomer 0% strain (black open symbols) and 1300% (black squares) strain. 67

**Figure S72**. Azimuthal angle profiles of the **CEPU3** elastomer for all q-values for increasing stretching 0% to 1400%. 68

**Figure S73**. The evolution of the 2D-SAXS pattern of **CEPU3** during relaxation from 1400% strain to 700%. 69

**Figure S74**. Azimuthal angle profiles of **CEPU3** for the relaxation from 1400% strain to 700%. 69

**Figure S75**. Azimuthal angle profile evolution of **CEPU3** at q = 1.02nm$^{-1}$ for each profile the mean value of $q$ was 1.02 nm$^{-1}$ for a range of $\Delta q$ = 0.13 nm$^{-1}$ (A) stretching and (B) relaxation. In the 1D plot the azimuthal profiles are systematically shifted for sake of visibility by an arbitrary constant intensity. 70

**Figure S76**. Selected 1D-SAXS pattern of **CEPU3** as a function of strain from 0% to 500% taken in the center of the flap (A) and the edge (B). 70

**Figure S77**. Selected 2D-SAXS pattern of **CEPU3** as a function of strain from 0% to 500% taken in the center of the flap (a-k) and the edge (b-l). 71

**Figure S78**. The evolution of 2D-SAXS pattern of **CEPU4** as a function of strain from 0% to a maximum of 1400%. 71

**Figure S79**. Azimuthal angle profiles of the **CEPU4** elastomer for all q-values for increasing stretching 0% to 1400%. 72

**Figure S80**. Azimuthal angle profiles of the **CEPU4** elastomer for all q-values for increasing stretching 0% to 1400%. 73

**Figure S81**. Azimuthal angle profiles of the **CEPU4** elastomer for all q-values for increasing stretching 0% to 1400%. 73

**Figure S82**. Selected 1D-SAXS pattern of **CEPU4** as a function of strain from 0% to 500% taken in the center of the flap (A) and the edge (B). 73

**Figure S83**. Selected 2D-SAXS pattern of **CEPU4** as a function of strain from 0% to 500% taken in the center of the flap (a-k) and the edge (b-l). 74

**Figure S84**. The evolution of the 2D-SAXS pattern of **CEPU4** during relaxation from 1400% strain to 700%. .......... 74

**Figure S85**. 1D-SAXS pattern for **CEPU3** as a function of strain during cycle elongation from 0% to 100% to 20% at 20% intervals. .......... 75

**Figure S86**. 2D-SAXS pattern for **CEPU3** as a function of strain during cycle elongation from 0% to 100% to 20% at 20% intervals. .......... 75

**Figure S87**. Azimuthal profile for the **CEPU3** elastomer as a function of strain during cycle elongation from 0% to 100% to 20% at 20% intervals. .......... 76

**Figure S88**. 1D-SAXS pattern for **CEPU4** as a function of strain during cycle elongation from 0% to 100% to 40% at 20% intervals. .......... 76

**Figure S89**. 2D-SAXS pattern for **CEPU4** as a function of strain during cycle elongation from 0% to 100% to 40% at 20% intervals. .......... 77

**Figure S90.** (A) cyclic compression (B) load-relax-reload compression. .......... 78

**Figure S91**. Parameters measured during cyclic tensile test. Calculation of elastic recovery (ER), $\varepsilon_{max}$ (maximum strain), $\varepsilon_{min}$ (minimum strain), residual strain ($\varepsilon_R$).[2] .......... 78

**Figure S92**. (A) Single cyclic compression tensile tests of **CEPU4** at 25 °C under various deformations of 20%, 40%, 60% and 80% in successive stretching, (B) Compression hysteresis energy recovery tests for **CEPU4** at two various deformation 20% and 40% at 25 °C, after relaxing, the cycle curve was overlapped with the original cycle (C) Compressive strain-dependent energy dissipation ratios and dissipated energies of **CEPU4**, and (D) Comparison of elastic recovery of **CEPU4**. .......... 79

**Figure S93**. Consecutive compression cyclic tensile tests of **CEPU4** at 25 °C without relaxation, specimens were compressed ten times at 20% deformation. .......... 80

**Figure S94**. Speckle pattern used for DIC in tension tests. .......... 80

**Figure S95**. Comparison of DIC and crosshead displacements. .......... 81

**Figure S96**. Strain field evolution on the surface of the CEPU specimen. .......... 82

**Figure S97**. Axial deformation over a cross-section of the specimen during the tensile test. .......... 83

**Figure S98**. Incompressibility (Volume Conservation) V'/V over a cross-section of the specimen during the tensile test. .......... 84

**Figure S99**. Photographs depicting shape memory performance of **CEPU3**. .......... 84

**References** .......... 85

## General synthetic protocol for CEPUs.

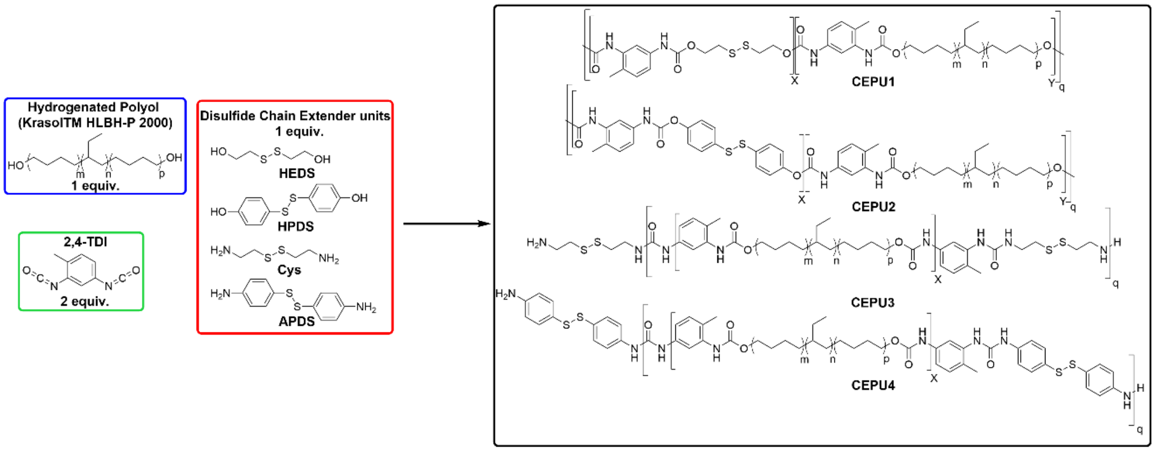


**Figure S1**: The synthetic route used to synthesis **CEPU1**-**CEPU4**.

A typical process to prepare CEPUs is described in the following text. Briefly, hydrogenated poly(butadiene) diol (Krasol HLBH-P 2000), molecular weight as supplied = 2100 g mol$^{-1}$,was dried under vacuum at 80 °C for 3 hours prior to use. For the one-step route, the dried polymer diol (10.00 g, 5 mmol, 1 equiv.) and disulfide chain extender unit (5 mmol, 1 equiv.) were mixed together and dissolved in 100 mL dry THF at 50 °C, toluene-2,4-diisocyanate (2,4-TDI) (1.67 g, 10 mmol, 2 equiv.) along with catalyst (dibutyltin dilaurate (DBTDL 0.5 wt.% reactants)) were then added to the mixture. For the two-step route, in the bulk, the dried polymer diol (10.00 g, 5 mmol, 1 equiv.) was reacted with 2,4-TDI (1.67 g, 10 mmol, 2 equiv.) with catalyst (DBTDL 0.5 wt.% reactants) were then added to the mixture, after reacting for 3 hours under an argon atmosphere at 80 °C, the desired isocyanate terminated polyurethane prepolymer (NCO:OH ratio of 2:1) was created. The prepolymer was then solvated in dry THF (100 mL) at 50 °C and the disulfide chain extender unit (10 mmol, 1 equiv.) was then added. For both synthetic routes (one and two steps) the mixture was stirred for 18 hours at 60 °C to obtain the desired CEPUs. Each reaction was monitored via FT-IR spectroscopy, until the isocyanate absorbance band at 2272-2248 cm$^{-1}$ was no longer evident, a period of *ca.* 18 hours was typically required. The CEPUs were purified and isolated after repeated precipitations into ice-cold methanol (3 × 900 mL). CEPUs films were subsequently solvent cast from a concentrated solution of THF (3 g/mL$^{-1}$). The viscous polymer solution was poured into a 10 cm × 10 cm mould with a PTFE base and then placed into an oven at 60 °C for 18 hours; then under partial

vacuum (*ca*. 600 mbar) at 60 °C for 24 hours, the polymer film was then allowed to reach room temperature before being removed from the mould and a polymer film was obtained, see **Figure S1**.

**Synthesis of CEPU1**

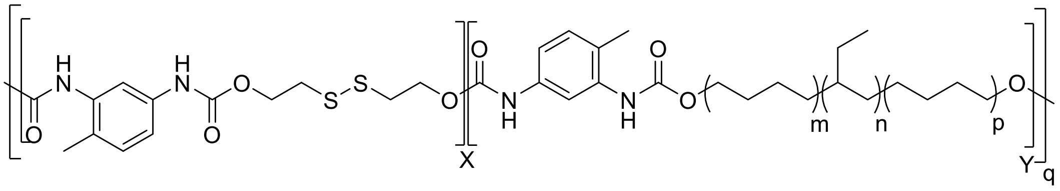

The synthesis was carried out according to the general synthetic protocol described above for CEPUs; **CEPU1** was obtained as a yellow transparent elastomeric solid (13.41 g, 90%). $T_g$ = -45.82 °C; FT-IR ATR (ν/cm$^{-1}$): 3315 (νN-H$_{stretch}$), 2958 (νC-H$_{alkyl}$), 2919 (νC-H$_{alkyl}$), 2851 (νC-H$_{alkyl}$), 1707 (νC=O$_{urethane}$), 1533 (C-N$_{stretch}$), 1459 (νC-H$_{alkyl}$), 1378 (νC-H$_{alkyl}$); Raman spectra (cm$^{-1}$): *ca.* 641 (νC-S$_{stretch}$), *ca.* 513 (νS-S$_{stretch}$); $^1$H NMR (400 MHz, THF-$d_8$) δ 8.79 (s, 2H), 8.60 (s, 2H), 7.62 (d, 4H), 7.42 – 7.24 (m, 5H), 6.99 (d, 4H), 4.43 – 4.30 (m, 8H), 4.16 – 4.01 (m, 8H), 3.05 – 2.97 (m, 8H), 2.49 (s, 5H), 2.16 (s, 12H), 2.02 (s, 10H), 1.70 – 0.64 (m, 760H); $^{13}$C NMR (100 MHz, THF-$d_8$) δ 154.8, 154.5, 139.1, 138.7, 138.1, 137.8, 131.2, 131.1, 126.1, 68.1, 67.9, 67.7, 40.1, 39.6, 39.6, 39.0, 38.6, 37.4, 34.6, 34.35, 31.7, 31.3, 30.9, 27.8, 27.7, 27.5, 27.3, 27.1, 26.9, 25.8, 25.6, 17.5, 11.4, 11.2, 10.9, 10.8; GPC (THF) $M_n$ = 17400 g mol$^{-1}$, $M_w$ = 45500 g mol$^{-1}$, Đ = 2.61.

**Synthesis of CEPU2**

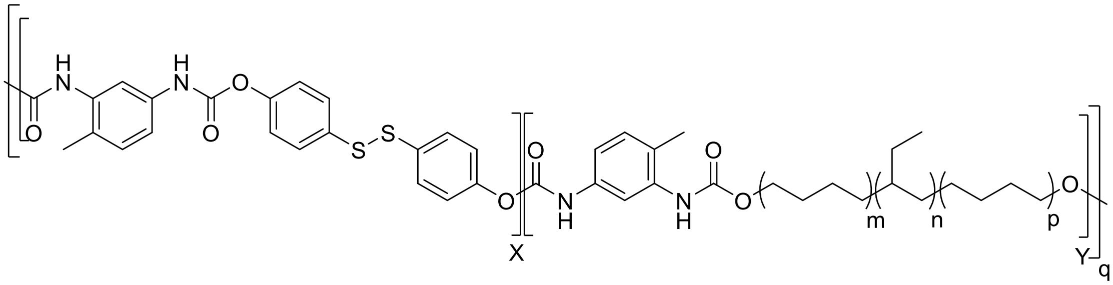

The synthesis was carried out according to the general synthetic protocol described above for CEPUs; **CEPU2** was obtained as a yellow opaque elastomeric solid (13.71 g, 92%). $T_g$

= -45.61 °C; FT-IR ATR (v/cm$^{-1}$): 3314 (vN-$H_{stretch}$), 2950 (vC-$H_{alkyl}$), 2919 (vC-$H_{alkyl}$), 2851 (vC-$H_{alkyl}$), 1711 (vC=$O_{urethane}$),1533 (C-$N_{stretch}$), 1459 (vC-$H_{alkyl}$), 1378 (vC-$H_{alkyl}$); Raman spectra (cm$^{-1}$): *ca.* 640 (vC-$S_{stretch}$), *ca.* 487 (vS-$S_{stretch}$); $^{1}$H NMR (400 MHz, THF-$d_8$) δ 9.28 (d, $J$ = 19.8 Hz, 2H), 8.60 (s, 3H), 7.72 (s, 3H), 7.64 – 6.95 (m, 28H), 6.72 – 6.65 (m, 4H), 4.17 – 4.04 (m, 9H), 3.64 – 3.60 (m, 34H), 2.51 (s, 6H), 2.29 – 2.23 (m, 3H), 2.18 (d, $J$ = 9.8 Hz, 10H), 2.08 – 1.96 (m, 8H), 1.80 – 1.75 (m, 33H), 1.70 – 0.82 (m, 796H); $^{13}$C NMR (100 MHz, THF-$d_8$) δ 159.7, 154.8, 154.5, 139.1, 138.3, 138.1, 134.3, 133.8, 131.3, 131.1, 130.9, 130.4, 123.5, 123.3, 117.1, 116.9, 68.4, 68.1, 40.1, 39.6, 39.5, 39.0, 37.3, 34.6, 34.3, 31.7, 31.3, 30.8, 27.8, 27.1, 26.9, 26.5, 26.0, 17.5, 11.4, 11.2; GPC (THF) $M_n$ = 15600 g mol$^{-1}$, $M_w$ = 49100 g mol$^{-1}$, Đ = 3.15.

**Synthesis of CEPU3**

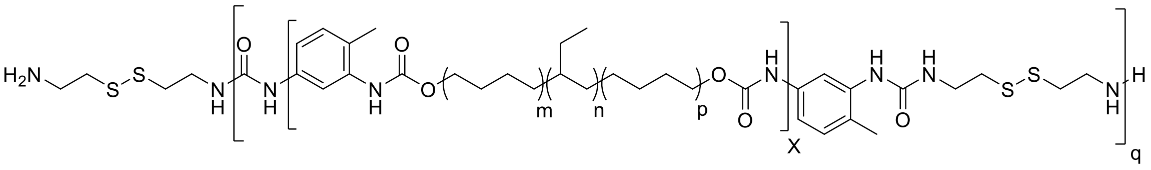


The synthesis was carried out according to the general synthetic protocol described above for CEPUs; **CEPU3** was obtained as a colourless elastomeric solid (14.00 g, 94%). $T_g$ = -45.11 °C; FT-IR ATR (v/cm$^{-1}$): 3323 (vN-$H_{stretch}$), 2950 (vC-$H_{alkyl}$), 2919 (vC-$H_{alkyl}$), 2851 (vC-$H_{alkyl}$), 1701 (vC=$O_{urethane}$), 1636 (vC=$O_{urea}$), 1544 (C-$N_{stretch}$), 1459 (vC-$H_{alkyl}$), 1378 (vC-$H_{alkyl}$), 1223 (vS=$O_{stretch}$); Raman spectra (cm$^{-1}$): *ca.* 639 (vC-$S_{stretch}$), *ca.* 510 (vS-$S_{stretch}$); $^{1}$H NMR (400 MHz, THF-$d_8$) δ 8.57 (s, 4H), 7.74 (s, 4H), 7.40 (s, 4H), 7.31 (s, 6H), 6.92 (d, $J$ = 8.2 Hz, 6H), 6.60 (s, 4H), 4.16 – 4.02 (m, 16H), 3.55 – 3.48 (m, 10H), 2.83 (t, $J$ = 6.6 Hz, 14H), 2.51 (s, 10H), 2.17 (s, 4H), 2.10 – 1.87 (m, 38H), 1.69 – 0.66 (m, 1333H); $^{13}$C NMR (100 MHz, THF-$d_8$) δ 156.9, 154.4, 139.0, 138.8, 130.9, 123.3, 114.6, 114.0, 113.1, 68.0, 67.8, 65.0, 63.3, 51.9, 40.0, 39.5, 39.4, 38.9, 37.2, 34.5, 34.2, 31.6, 31.2, 30.7, 27.7, 27.6, 27.4, 27.0, 26.8, 25.9, 25.7, 17.4, 11.3, 11.1; GPC (THF) $M_n$ = 25800 g mol$^{-1}$, $M_w$ = 217800 g mol$^{-1}$, Đ = 8.44.

**Synthesis of CEPU4**

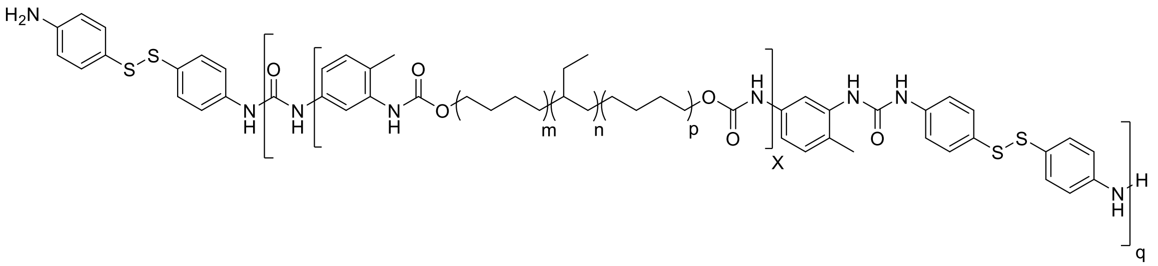

The synthesis was carried out according to the general synthetic protocol described above for CEPUs; **CEPU4** was obtained as a yellow transparent elastomeric solid (14.16 g, 95%). $T_g$ = -45.77 °C; FT-IR ATR (ν/cm$^{-1}$): 3299 (νN-H$_{stretch}$), 2950 (νC-H$_{alkyl}$), 2919 (νC-H$_{alkyl}$), 2851 (νC-H$_{alkyl}$), 1701 (νC=O$_{urethane}$), 1644 (νC=O$_{urea}$), 1536 (C-N$_{stretch}$), 1459 (νC-H$_{alkyl}$), 1378 (νC-H$_{alkyl}$), 1307 (νS=O$_{stretch}$); Raman spectra (cm$^{-1}$): *ca.* 633 (νC-S$_{stretch}$), *ca.* 486 (νS-S$_{stretch}$); $^{1}$H NMR (400 MHz, THF-$d_8$) δ 8.65 (s, 2H), 8.32 (s, 2H), 7.88 (s, 4H), 7.50 – 7.24 (m, 23H), 7.01 – 6.93 (m, 4H), 4.16 – 4.03 (m, 8H), 2.59 (s, 6H), 2.17 (s, 12H), 2.08 – 1.86 (m, 10H), 1.69 – 0.64 (m, 723H); $^{13}$C NMR (100 MHz, THF-$d_8$) δ 154.4, 152.9, 141.7, 139.2, 138.6, 131.9, 130.9, 130.2, 119.4, 68.0, 67.8, 67.6, 65.0, 63.3, 39.4, 38.9, 37.2, 34.5, 34.2, 31.6, 31.2, 30.7, 30.2, 27.7, 27.6, 3.00, 26.8, 25.9, 25.7, 25.50, 17.3, 11.3, 11.1; GPC (THF) $M_n$ = 11400 g mol$^{-1}$, $M_w$ = 53500 g mol$^{-1}$, Đ = 4.69.

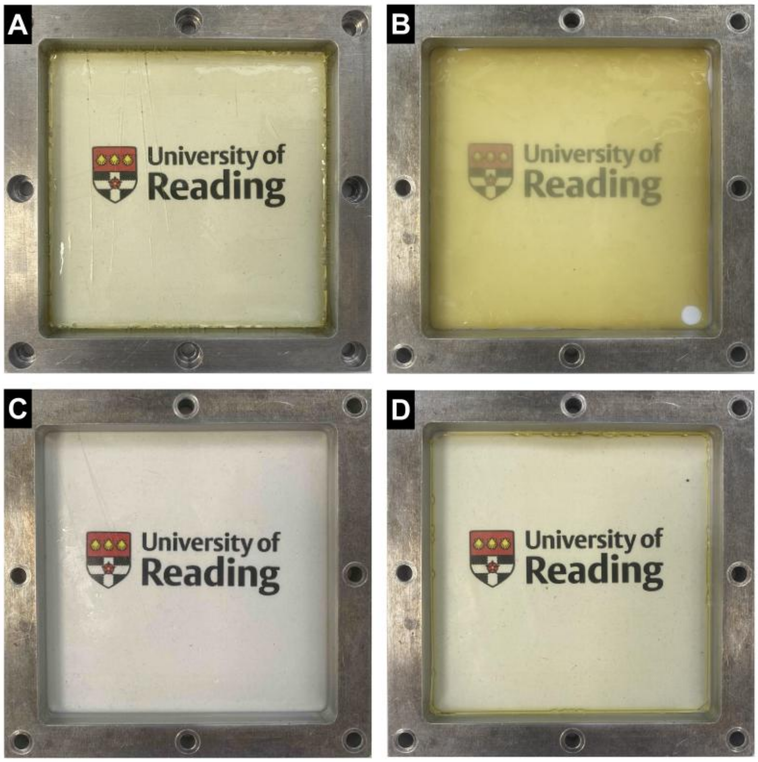


**Figure S2.** Representative homogeneous cast films (10 cm × 10 cm × 0.5 mm) of (A) **CEPU1**, (B) **CEPU2**, (C) **CEPU3**, and (D) **CEPU4** in aluminium moulds.

**Table S1**. The key $^{1}H$ and $^{13}C$ NMR spectroscopic resonances for **CEPU1-CEPU4** in THF-d8.

| CEPU | $^{1}H$ NMR urethane resonance (ppm) | $^{1}H$ NMR chain-extender urethane resonance (ppm) | $^{1}H$ NMR chain-extender urea resonance (ppm) | $^{13}C$ NMR urethane resonance (ppm) | $^{13}C$ NMR chain-extender urethane/urea resonance (ppm) |
|---|---|---|---|---|---|
| **CEPU1** | 8.60 | 8.79 | - | 154.5 | 154.8 (urethane) |
| **CEPU2** | 8.60 | 7.72 | - | 154.5 | 159.7 (urethane) |
| **CEPU3** | 8.61 | - | 7.74, 6.60 | 154.5 | 156.9 (urea) |
| **CEPU4** | 8.65 | - | 8.32, 7.88 | 154.5 | 152.9 (urea) |

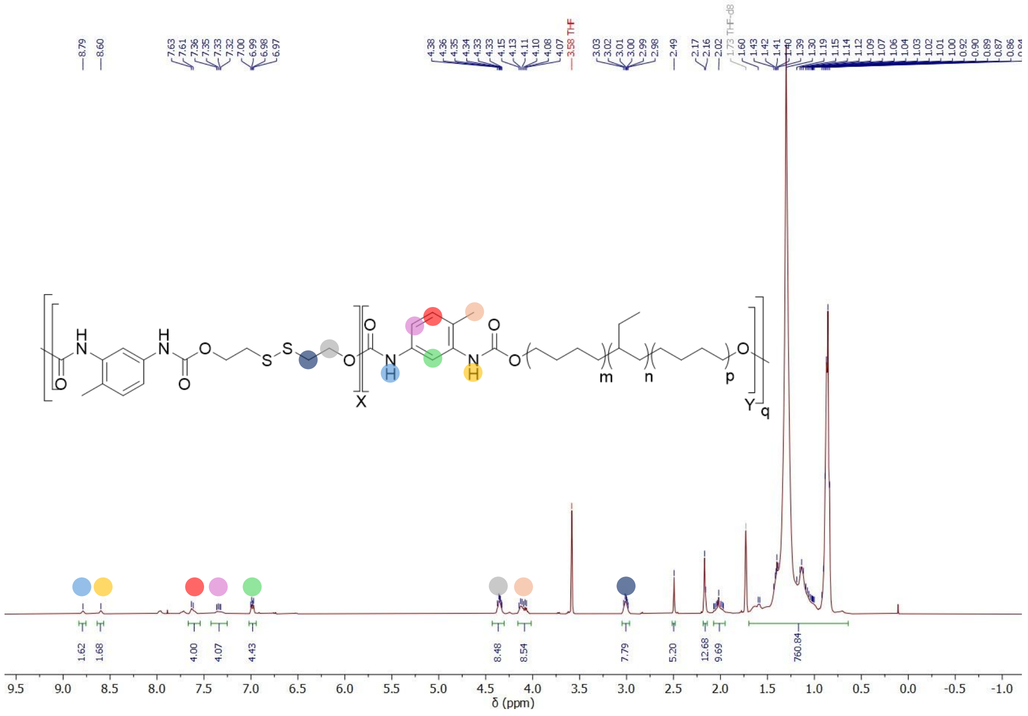


**Figure S3**. $^{1}$H NMR spectrum of **CEPU1** (400 MHz, THF-$d_8$, at 25 °C).

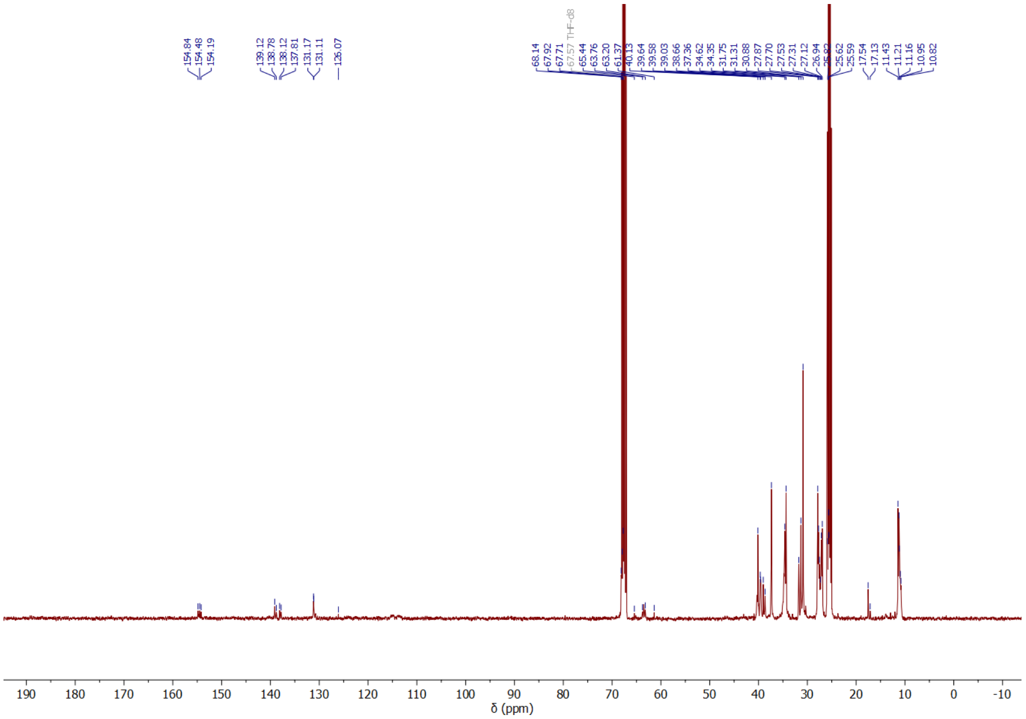


**Figure S4**. $^{13}$C NMR spectrum of **CEPU1** (100 MHz THF-$d_8$, at 25 °C).

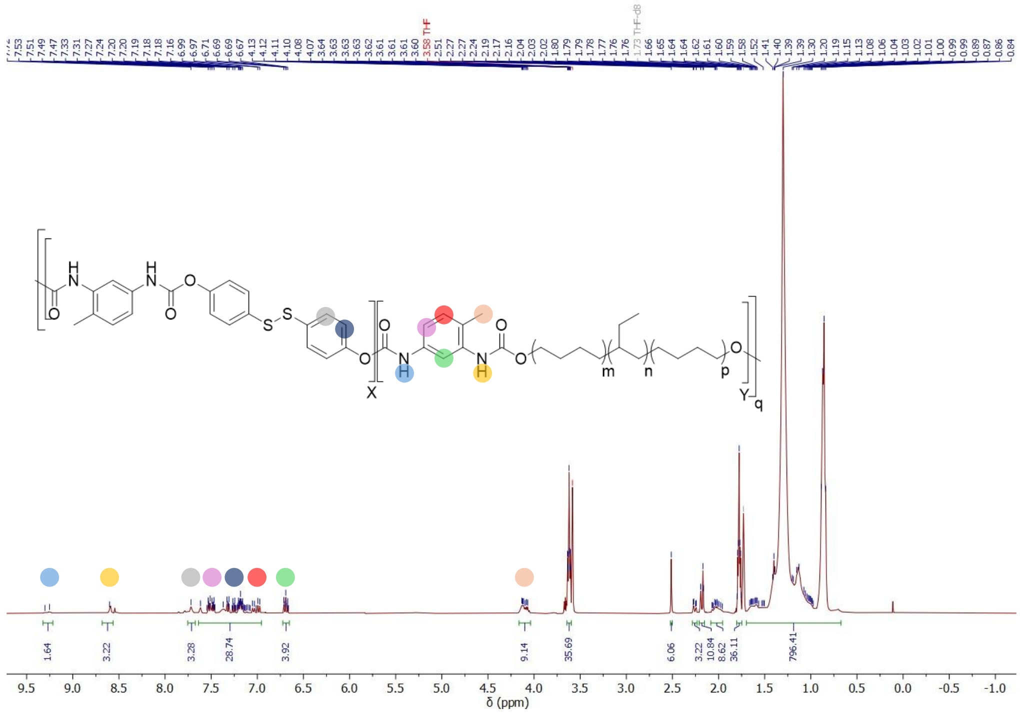

**Figure S5**. $^1$H NMR spectrum of **CEPU2** (400 MHz, THF-$d_8$, at 25 °C).

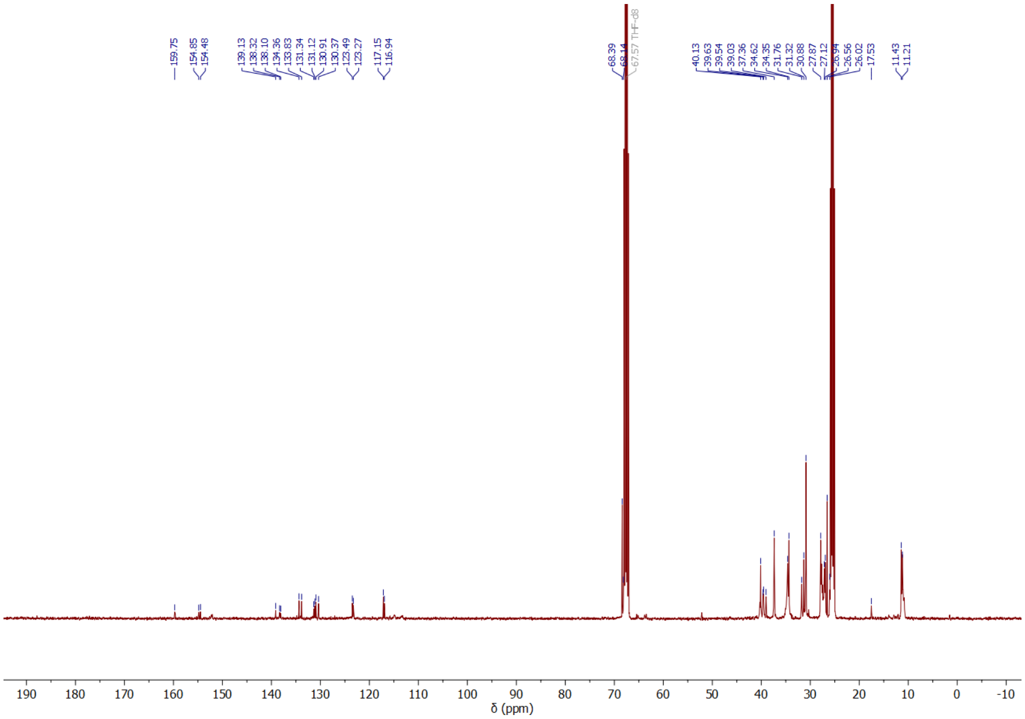

**Figure S6**. $^{13}$C NMR spectrum of **CEPU2** (100 MHz THF-$d_8$, at 25 °C).

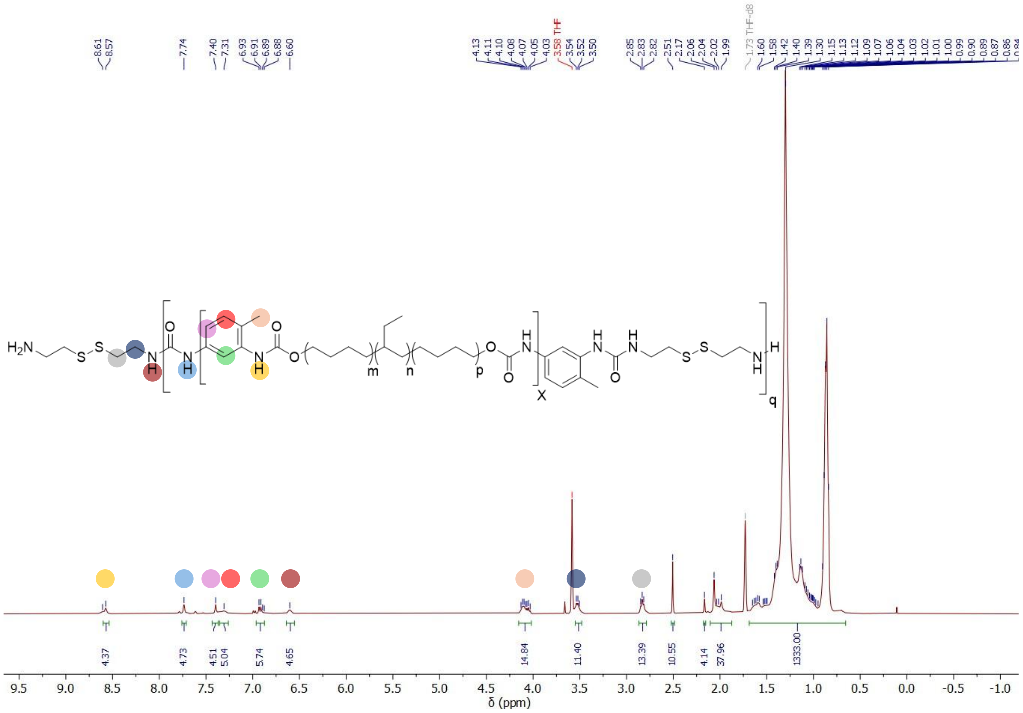

**Figure S7**. $^{1}$H NMR spectrum of **CEPU3** (400 MHz, THF-$d_8$, at 25 °C).

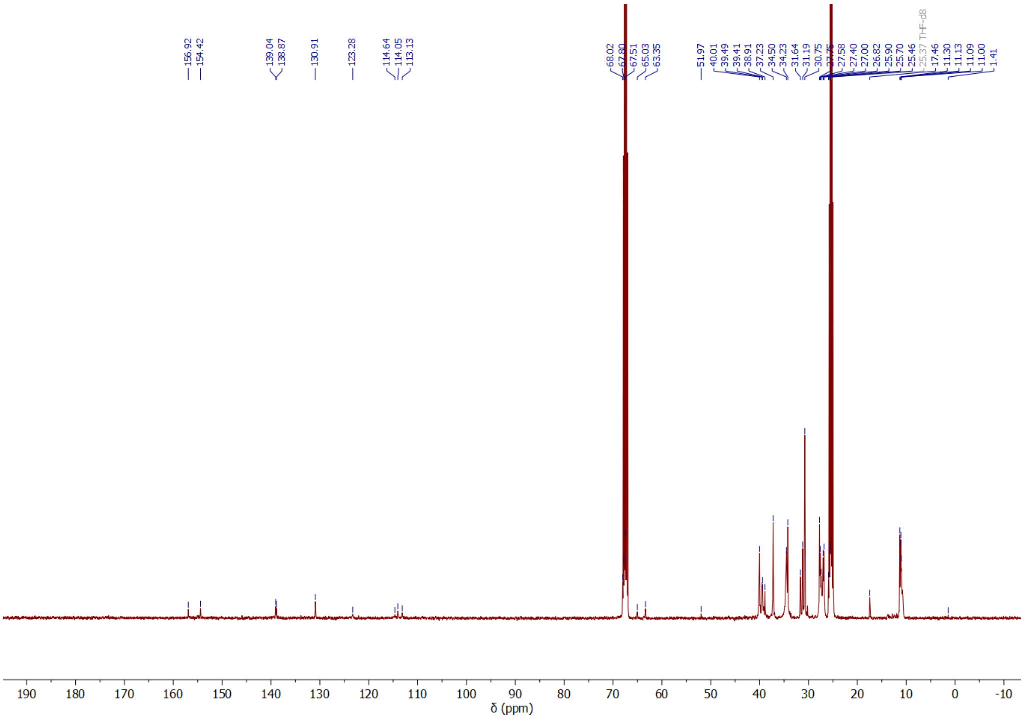

**Figure S8**. $^{13}$C NMR spectrum of **CEPU3** (100 MHz THF-$d_8$, at 25 °C).

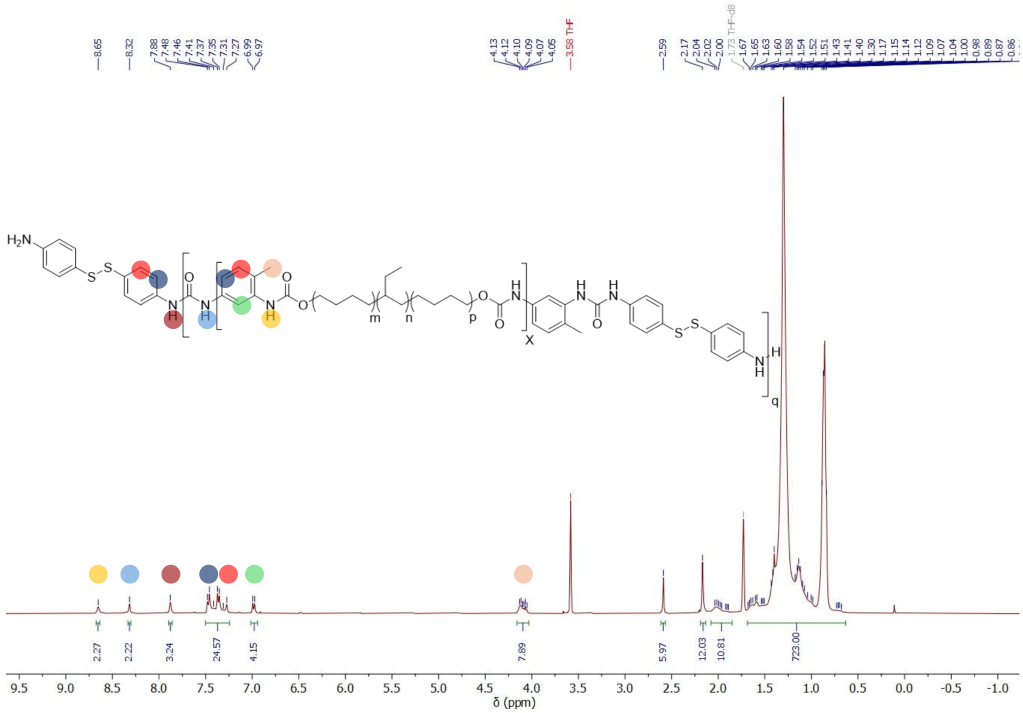

**Figure S9**. $^{1}$H NMR spectrum of **CEPU4** (400 MHz, THF-$d_8$, at 25 °C).

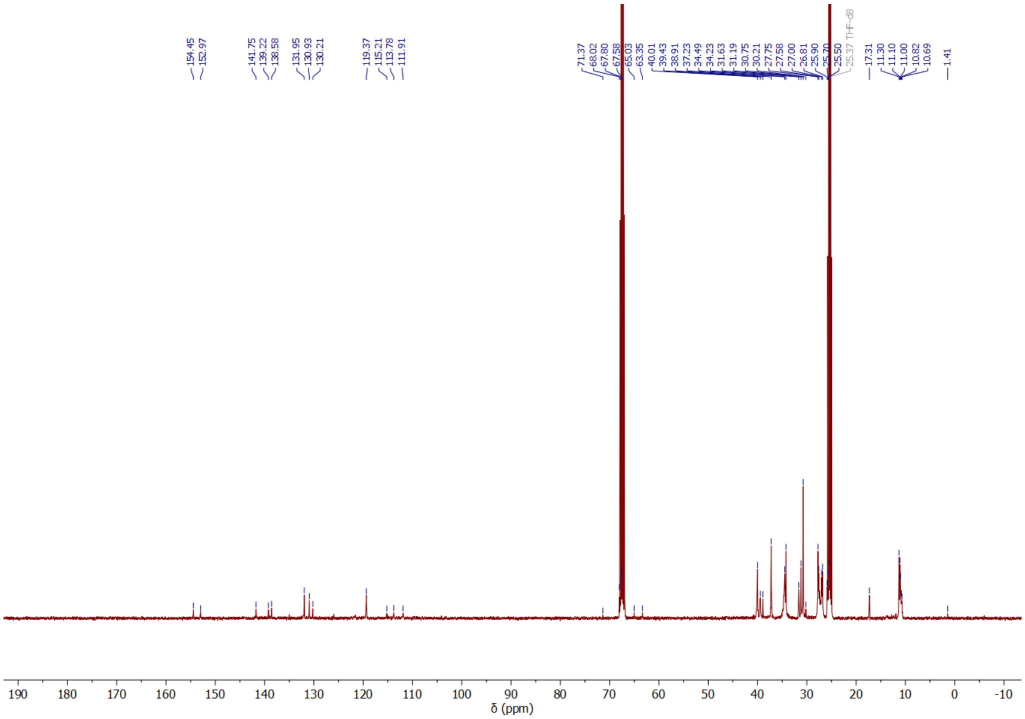

**Figure S10**. $^{13}$C NMR spectrum of **CEPU4** (100 MHz THF-$d_8$, at 25 °C).

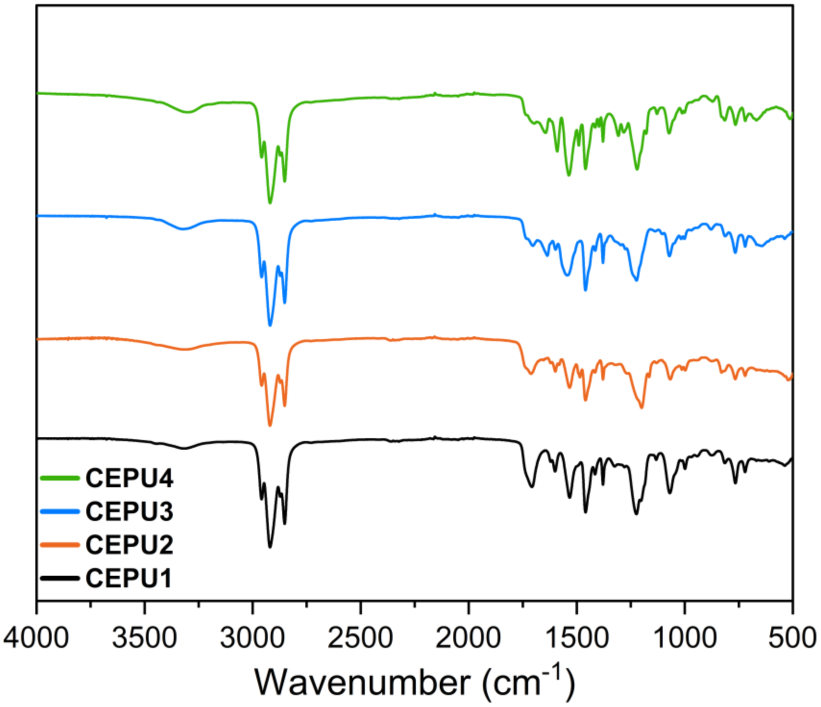


**Figure S11**. The FTIR spectrum of **CEPU1-CEPU4**.

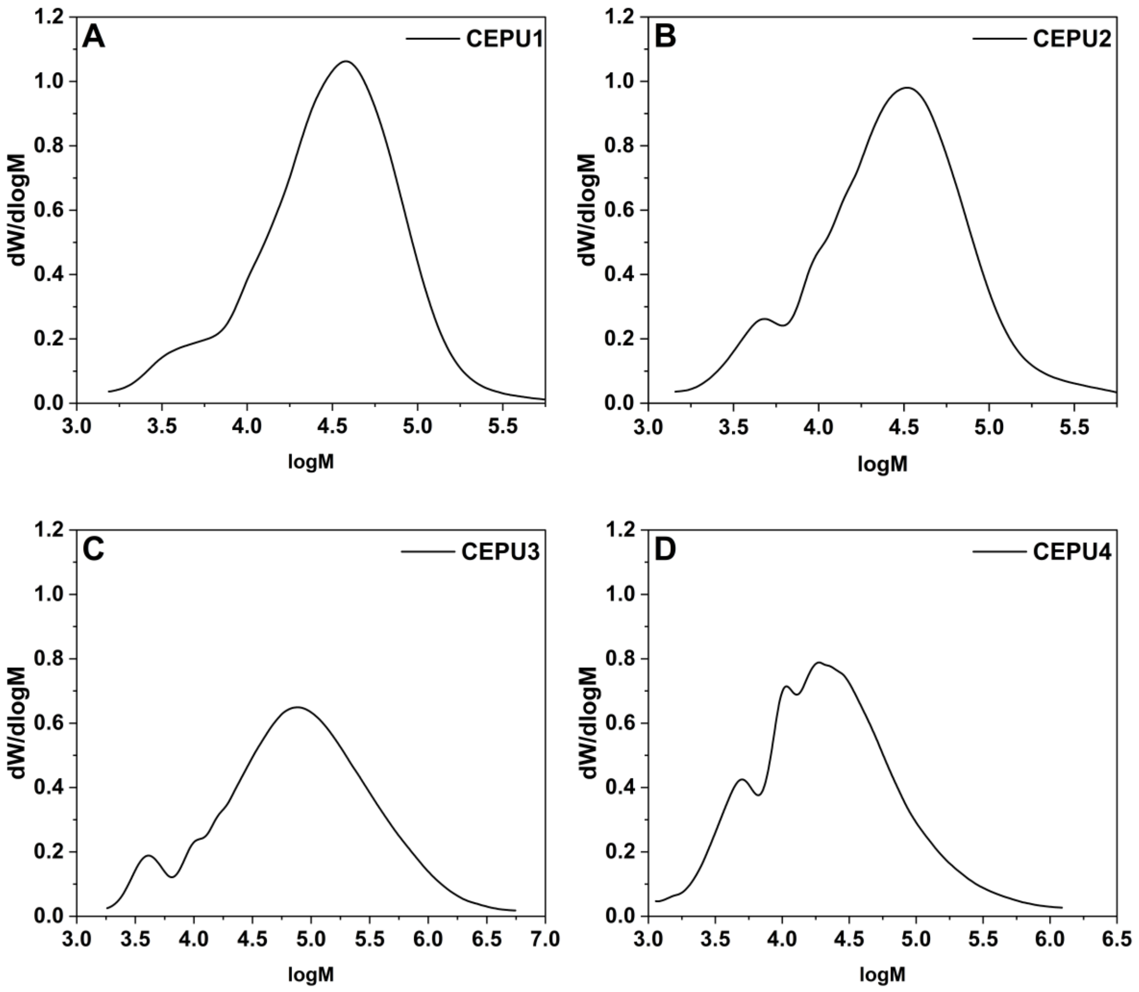


**Figure S12**. GPC eluograms of **CEPU1** (A), **CEPU2** (B), **CEPU3** (C), and **CEPU4** (D), in THF.

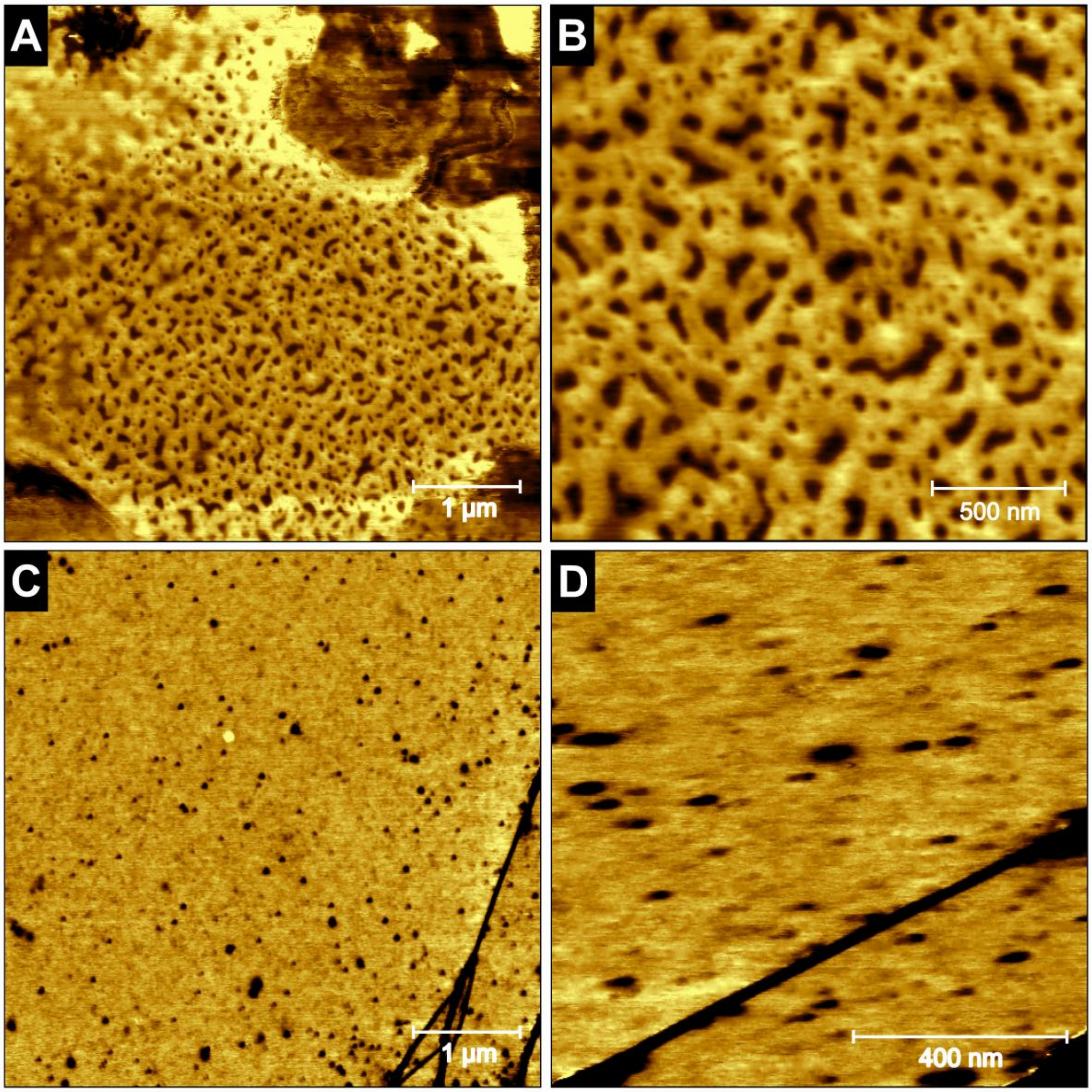


**Figure S13**. AFM tapping mode phase images of **CEPU1** (**A**, **B**) and **CEPU2** (**C**, **D**) for two different scale bars 1 µm and 500 nm. All polymer samples are prepared by drop casting from THF on the mica disc.

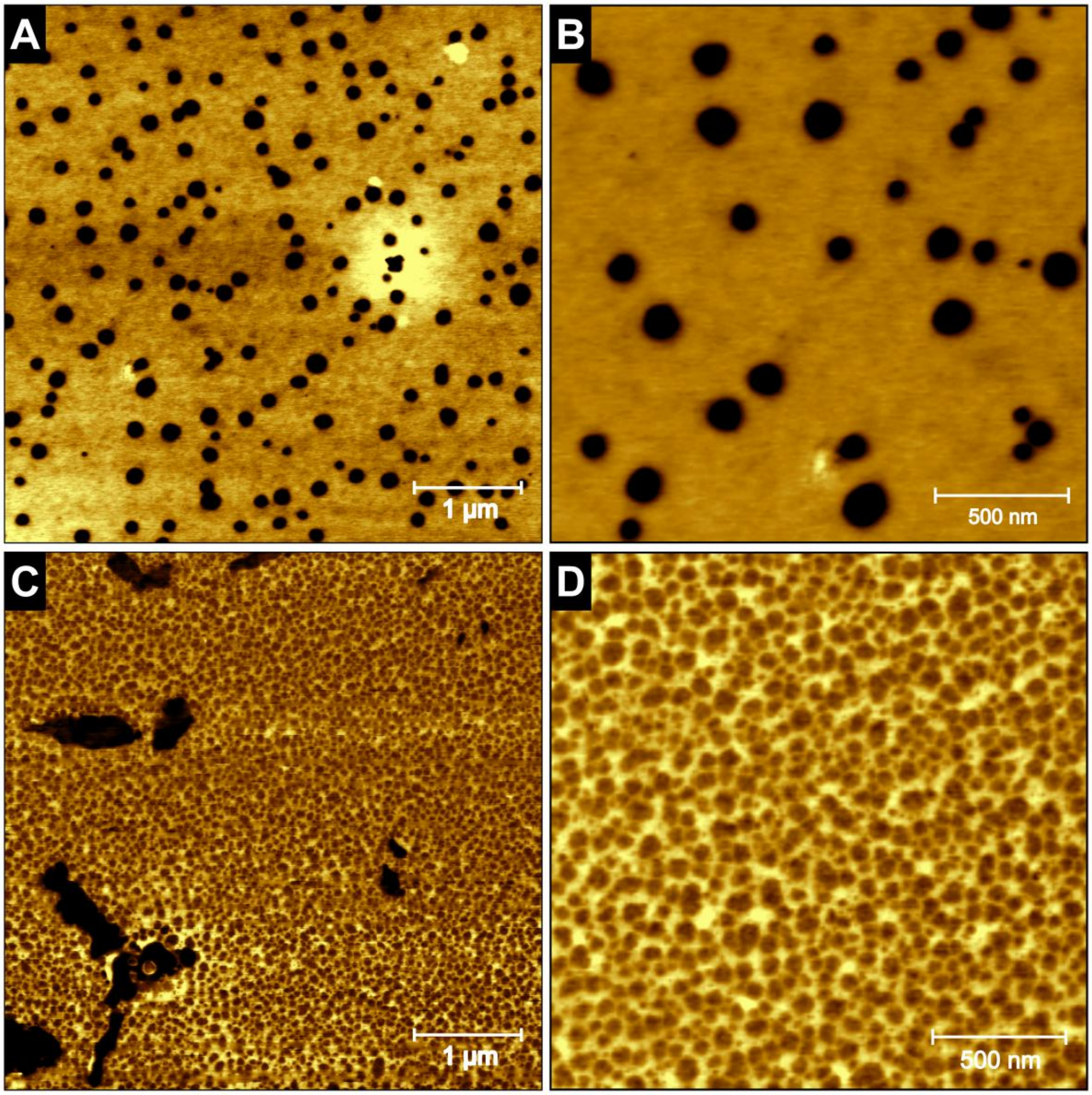


**Figure S14**. AFM tapping mode phase images of **CEPU3** (**A**, **B**) and **CEPU4** (**C**, **D**) for two different scale bars 1 µm and 500 nm. All polymer samples are prepared by drop casting from THF on the mica disc.

**General Synthesis of disulfide small molecule analogues**

A general synthetic protocol for the disulfide small molecule analogues is as follows. To a solution of phenyl isocyanate (2 equiv.) in dry THF (25 mL) the corresponding disulfide chain extender units (1 equiv.) were added dropwise. The resulting mixture was stirred overnight at 40 °C under Argon. Each reaction was monitored via FT-IR spectroscopy, until the isocyanate absorbance band at 2272-2248 $cm^{-1}$ was no longer evident. The solid product was then filtered and washed with THF (3 × 50 mL) and dried *in vacuo* to afford the desired product.

**Synthesis of 1,1'-(disulfanediylbis(ethane-2,1-diyl))bis(3-phenylurea) (1).**

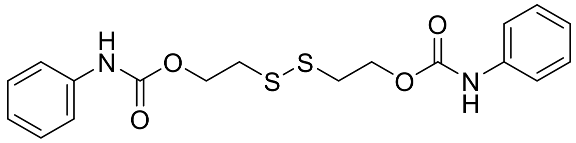

Analogue **1** was obtained as a white solid (2.21 g, 5.63 mmol, 87%) following the general method outlined above from phenyl isocyanate (1.54 g, 12.97 mmol, 2 equiv.) and bis(2-hydroxyethyl) disulfide (1.00 g, 6.48 mmol, 1 equiv.).

FT-IR ATR (v/$cm^{-1}$): 3344 ($vN\text{-}H_{stretch}$), 1704 ($vC{=}O_{urethane}$), 1532 ($vC{=}C_{Ar\text{-}stretch}$), 1232 ($vC\text{-}N_{stretch}$); $^{1}H$ NMR (400 MHz, DMSO-$d_6$) δ 9.71 (s, 2H, Ar-*NH*), 7.50 – 7.42 (m, 4H, Ar-*H*), 7.30 – 7.22 (m, 4H, Ar-*H*), 7.02 – 6.94 (m, 2H, Ar-*H*), 4.34 (t, *J* = 6.3 Hz, 4H, Ar-NHC(O)O$CH_2CH_2$), 3.06 (t, *J* = 6.3 Hz, 4H, 4H, Ar-NHC(O)O$CH_2CH_2$); $^{13}C$ NMR (100 MHz, DMSO-$d_6$) δ 153.3, 138.9, 128.7, 122.5, 118.3, 61.9, 36.8; FT-MS (ESI) *m/z* [M + $H^+$] calculated for $C_{18}H_{21}N_2O_4S_2$ = 393.0864; found = 393.0957.

**Synthesis of disulfanediylbis(4,1-phenylene) bis(phenylcarbamate) (2).**

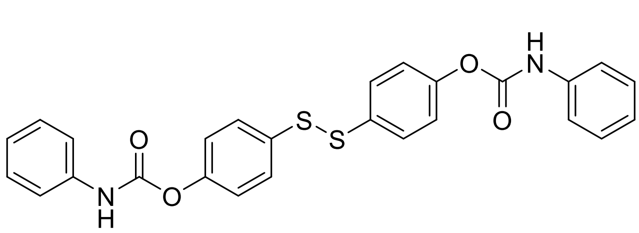

Analogue **2** was obtained as a white solid (1.63 g, 3.33 mmol, 83%) following the general method outlined above from phenyl isocyanate (0.951 g, 7.99 mmol, 2 equiv.) and bis(4-hydroxyphenyl) disulfide (1.00 g, 3.99 mmol, 1 equiv.).

FT-IR ATR (v/$cm^{-1}$): 3325 ($vN\text{-}H_{stretch}$), 1645 ($vC{=}O_{urethane}$), 1543 ($vC{=}C_{Ar\text{-}stretch}$), 1229 ($vC\text{-}N_{stretch}$); $^{1}H$ NMR (400 MHz, DMSO-$d_6$) δ 10.27 (s, 2H, Ar-*NH*), 7.63 – 7.57 (m, 4H), 7.53

– 7.47 (m, 4H, Ar-*H*), 7.38 – 7.23 (m, 8H, Ar-*H*), 7.07 – 7.02 (m, 2H, Ar-*H*).; $^{13}$C NMR (100 MHz, DMSO-*d*$_6$) δ 151.8, 150.7, 138.9, 132.9, 129.5, 129.4, 123.6, 118.9; FT-MS (ESI) *m/z* [M + H$^+$] calculated for $C_{26}H_{21}N_2O_4S_2$ = 489.0864; found = 489.0939.

**Synthesis of 1,1'-(disulfanediylbis(ethane-2,1-diyl))bis(3-phenylurea) (3).**

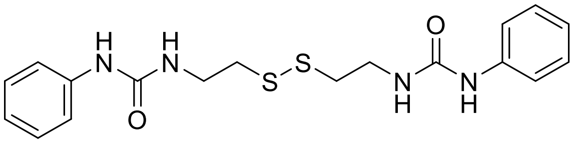


Analogue **3** was obtained as a white solid (2.41 g, 5.49 mmol, 94%) following the general method outlined above from phenyl isocyanate (1.56 g, 13.13 mmol, 2 equiv.) and cystamine (1.00 g, 6.57 mmol, 1 equiv.).

FT-IR ATR (ν/cm$^{-1}$): 3333 (νN-H$_{stretch}$), 1641 (νC=O$_{urethane}$), 1555 (νC=C$_{Ar\text{-}stretch}$), 1233 (νC-N$_{stretch}$); $^1$H NMR (400 MHz, DMSO-*d*$_6$) δ 8.56 (s, 2H, Ar-*NH*), 7.40 – 7.35 (m, 4H, Ar-*H*), 7.23 – 7.17 (m, 4H, Ar-*H*), 6.91 – 6.85 (m, 2H, Ar-*H*), 6.34 (t, *J* = 5.8 Hz, 2H, Ar-NHC(O)*NH*), 3.44 – 3.37 (m, 4H, Ar-NHC(O)NH*CH*$_2$CH$_2$), 2.84 (t, *J* = 6.6 Hz, 4H, Ar-NHC(O)NHCH$_2$*CH*$_2$); $^{13}$C NMR (100 MHz, DMSO-*d*$_6$) δ 155.1, 140.3, 128.6, 121.1, 117.7, 38.2, 37.9; FT-MS (ESI) *m/z* [M + H$^+$] calculated for $C_{18}H_{23}N_4O_2S_2$ = 391.1184; found = 391.1271.

**Synthesis 1,1'-(disulfanediylbis(4,1-phenylene))bis(3-phenylurea) (4).**

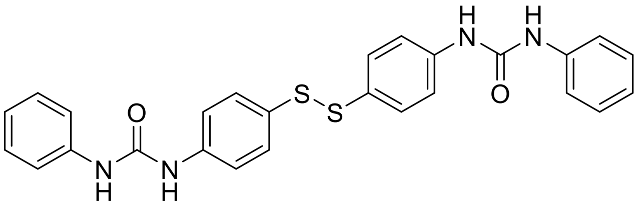


Analogue **4** was obtained as a white solid (1.88 g, 3.86 mmol, 95%) following the general method outlined above from phenyl isocyanate (0.959 g, 8.05 mmol, 2 equiv.) and 4-aminophenyl disulfide (1.00 g, 4.03 mmol, 1 equiv.).

FT-IR ATR (ν/cm$^{-1}$): 3301 (νN-H$_{stretch}$), 1635 (νC=O$_{urethane}$), 1553 (νC=C$_{Ar\text{-}stretch}$), 1234 (νC-N$_{stretch}$); $^1$H NMR (400 MHz, DMSO-*d*$_6$) δ 8.85 (s, 2H, Ar-*NH*), 8.71 (s, 2H, Ar-NHC(O)*NH*), 7.51 – 7.40 (m, 13H, Ar-*H*), 7.31 – 7.24 (m, 4H, Ar-*H*), 7.00 – 6.95 (m, 2H, Ar-*H*); $^{13}$C NMR (100 MHz, DMSO-*d*$_6$) δ 152.80, 140.67, 139.9, 131.3, 129.3, 128.5, 122.5, 119.32, 118.7; FT-MS (ESI) *m/z* [M + H$^+$] calculated for $C_{26}H_{23}N_4O_2S_2$ = 487.1184; found = 487.1261.

**Protocol for solution state $^1$H NMR kinetic study of chains exchange disulfide small molecule analogues**

Model small molecules (**3**:**4**) were made to 10 mg mL$^{-1}$ of (1:1 mole equiv.) in DMSO-$d_6$. The small molecule analogues were mixed, added directly to the NMR tube, and $^1$H NMR spectra were recorded at regular time intervals at room temperature and after heating to 80 °C for 60 and 120 minutes.

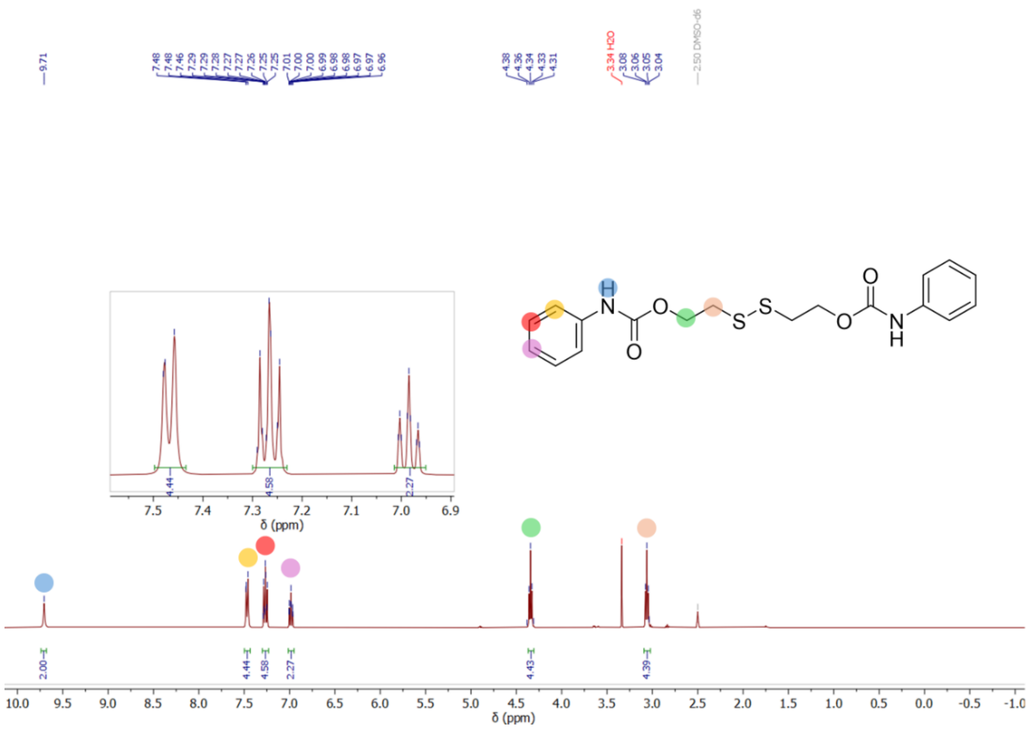

**Figure S15**. $^1$H NMR spectrum of analogues **1** (100 MHz, DMSO-$d_6$, at 25 °C).

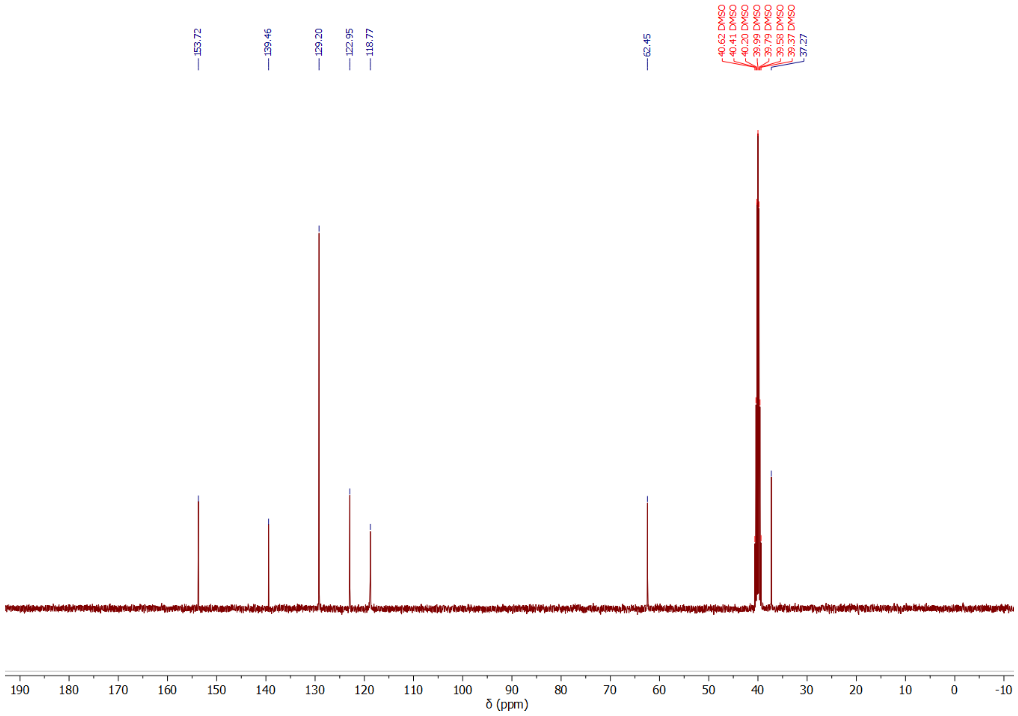


**Figure S16**. $^{13}C$ NMR spectrum of **analogue 1** (400 MHz, DMSO-$d_6$, at 25 °C).

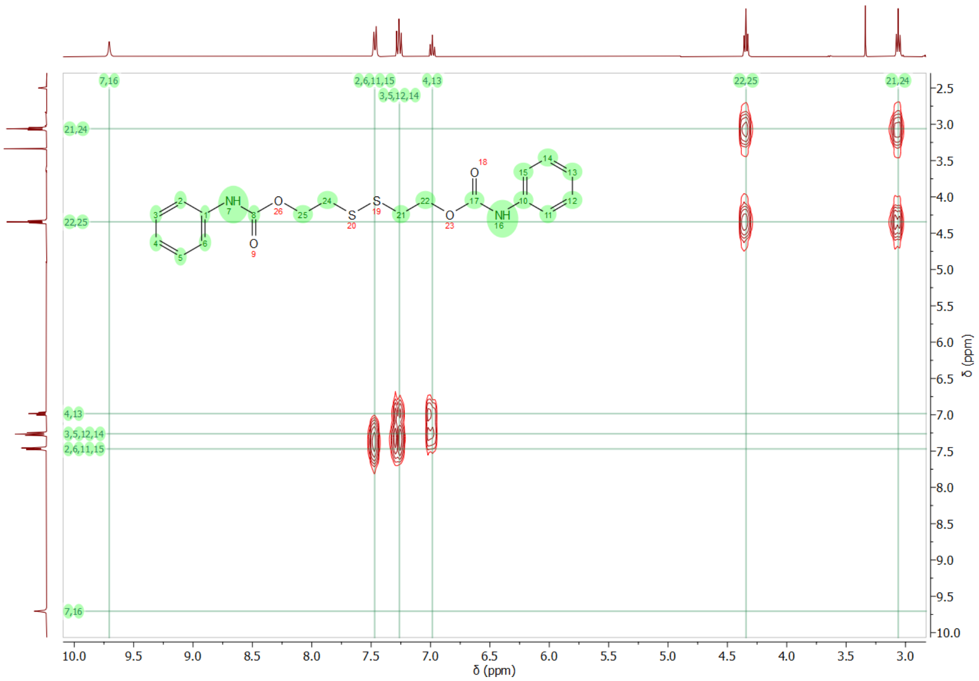


**Figure S17**. 2D COSY ($^1H$-$^1H$) NMR spectrum of **analogue 1** (400 MHz, DMSO-$d_6$, at 25 °C).

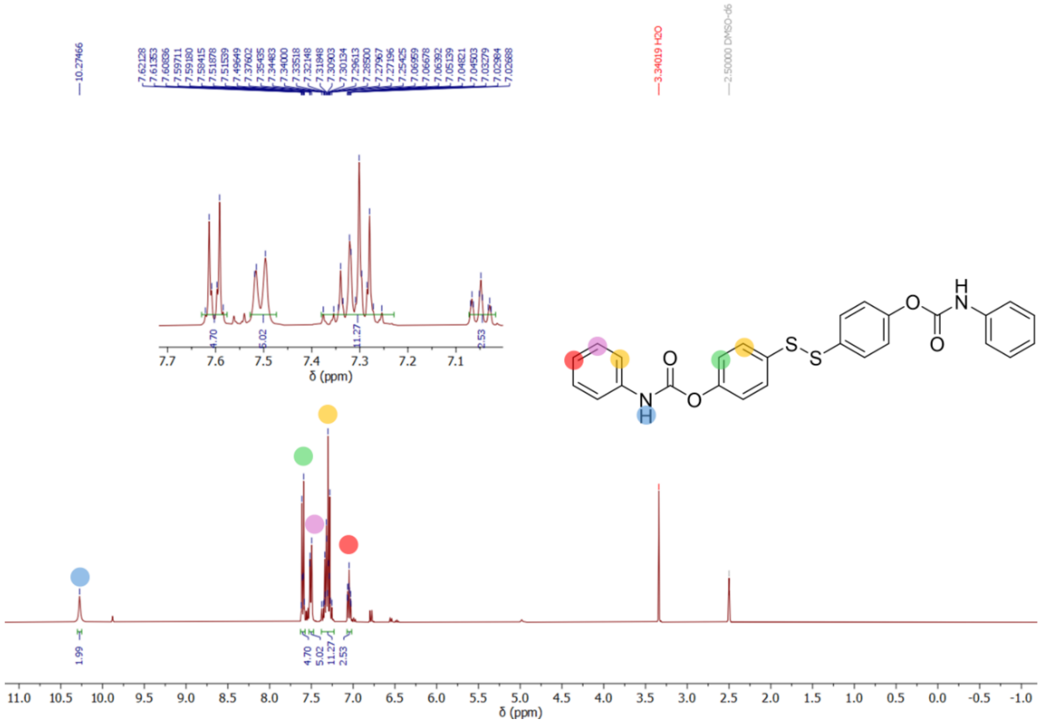


Figure S18. $^{1}$H NMR spectrum of **analogue 2** (400 MHz, DMSO-$d_6$, at 25 °C).

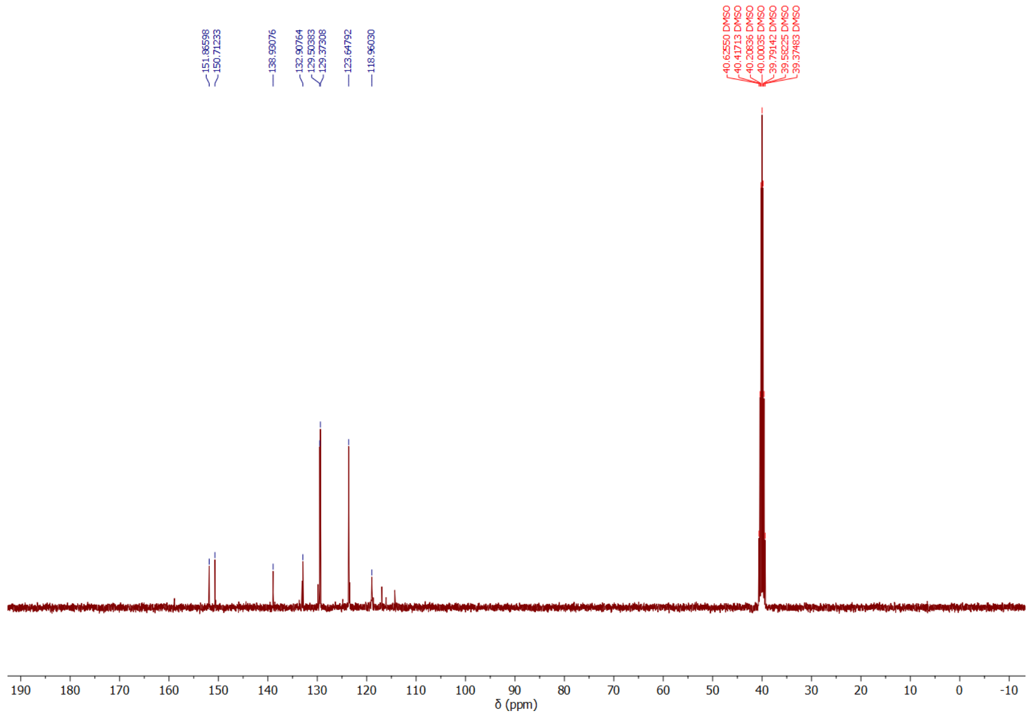


Figure S19. $^{13}$C NMR spectrum of **analogue 2** (100 MHz, DMSO-$d_6$, at 25 °C).

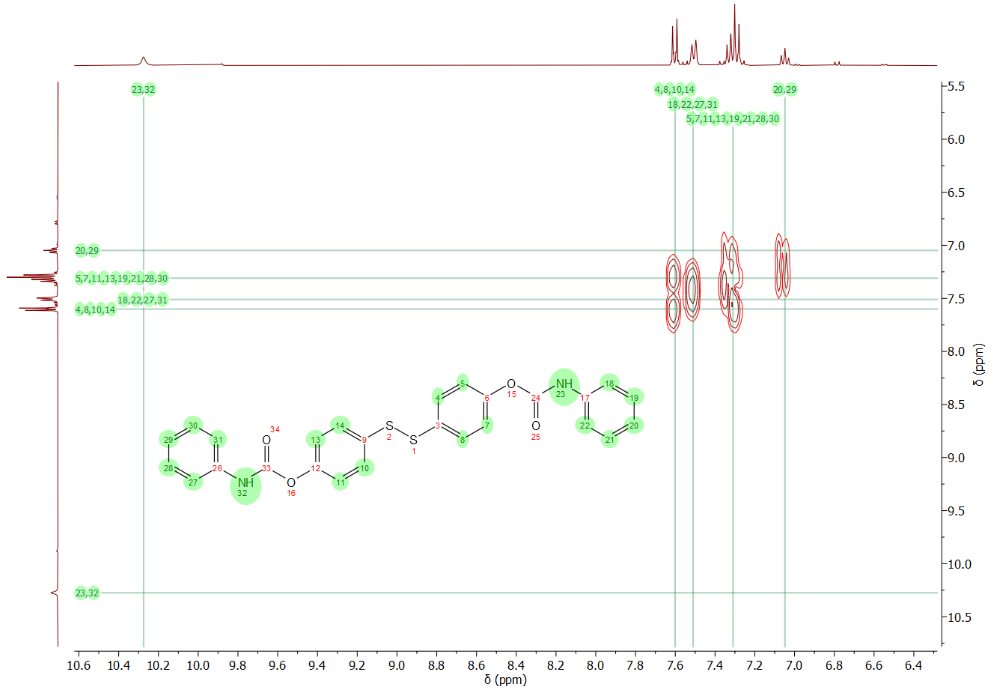

**Figure S20**. 2D COSY ($^1H$-$^1H$) NMR spectrum of **analogue 2** (400 MHz, DMSO-$d_6$, at 25 °C).

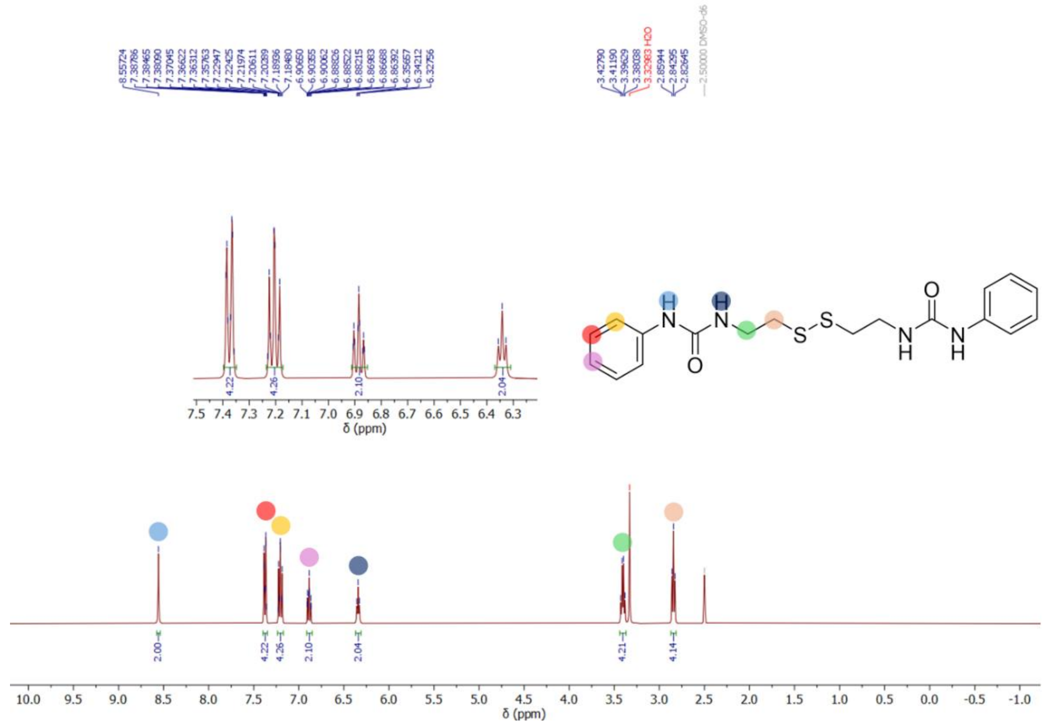

**Figure S21**. $^1H$ NMR spectrum of **analogue 3** (400 MHz, DMSO-$d_6$, at 25 °C).

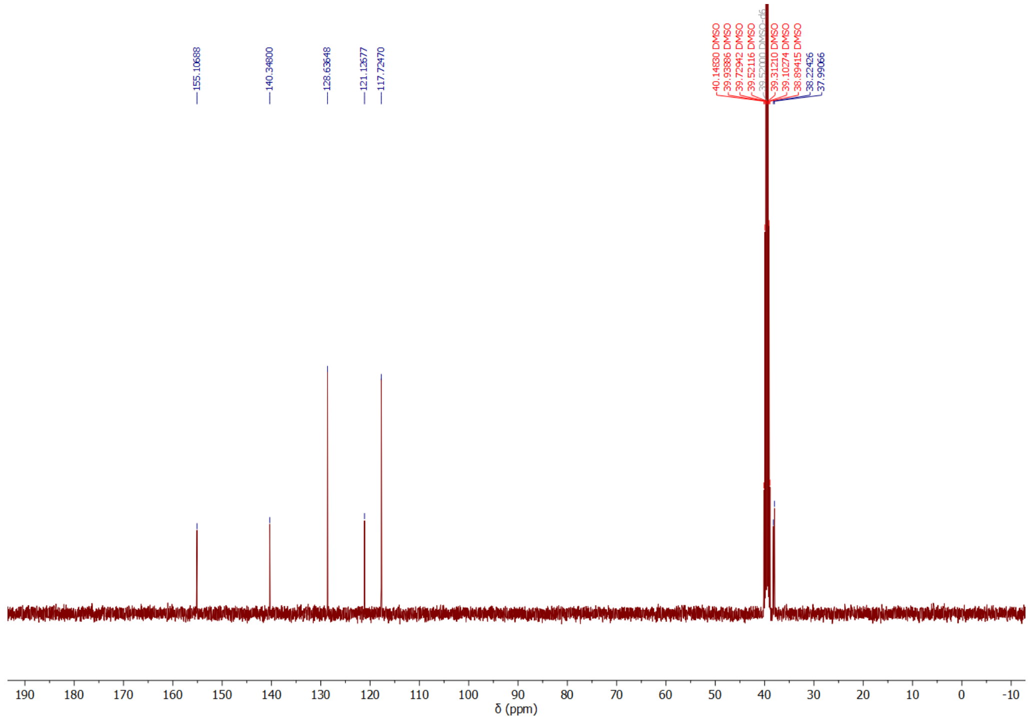

**Figure S22**. $^{13}C$ NMR spectrum of **analogue 3** (100 MHz, DMSO-$d_6$, at 25 °C).

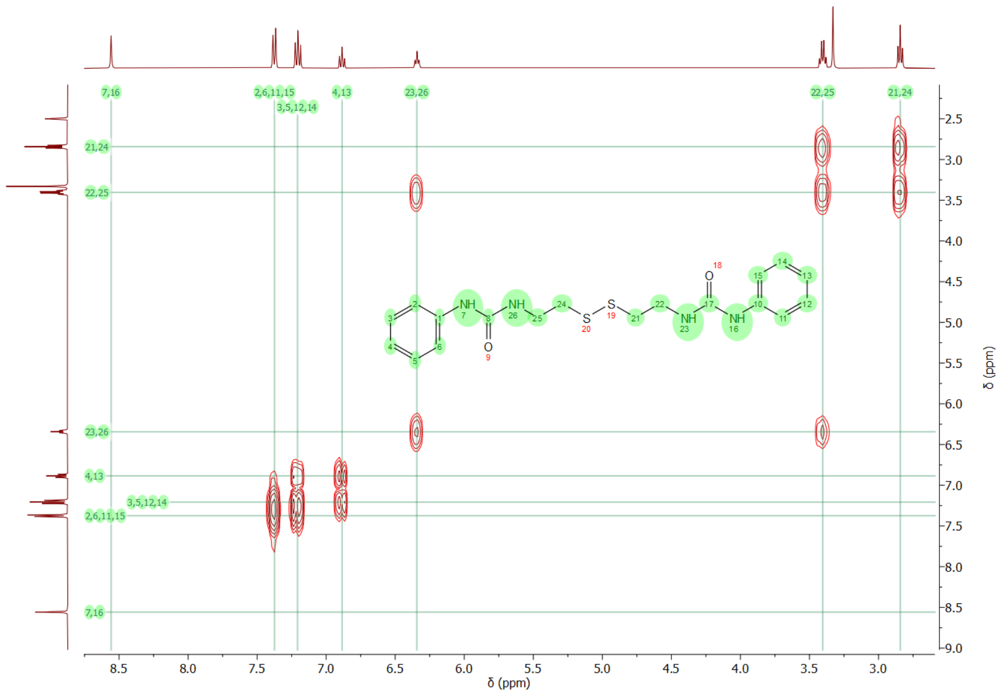

**Figure S23**. 2D COSY ($^1H$-$^1H$) NMR spectrum of **analogue 3** (400 MHz, DMSO-$d_6$, at 25 °C).

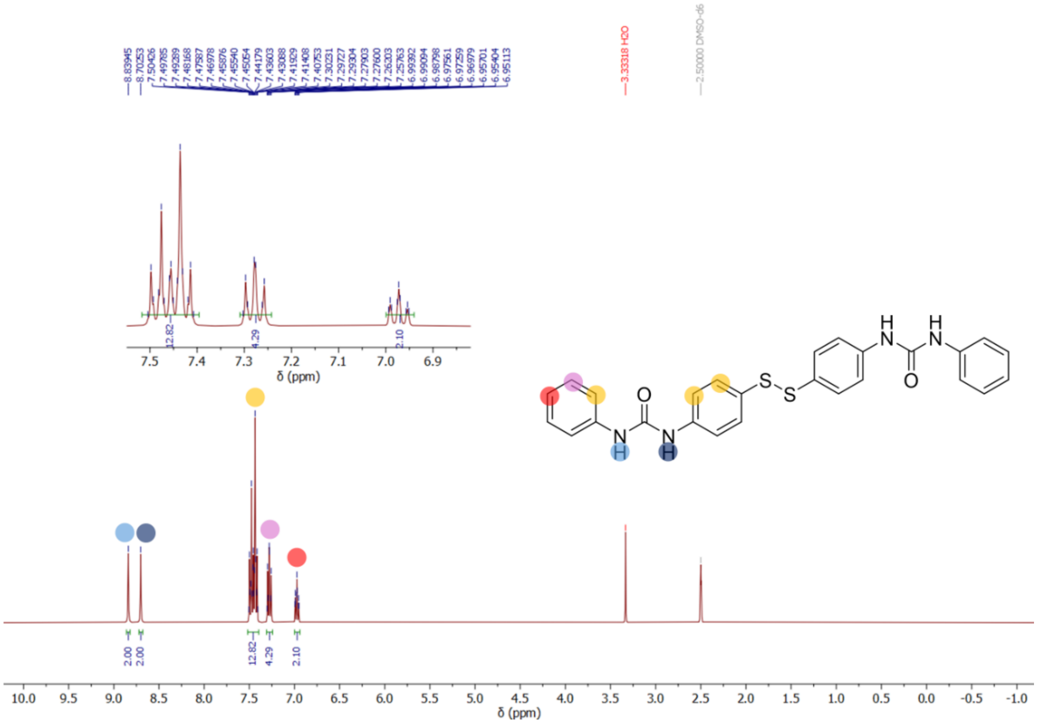


**Figure S24**. $^{1}$H NMR spectrum of **analogue 4** (400 MHz, DMSO-$d_6$, at 25 °C).

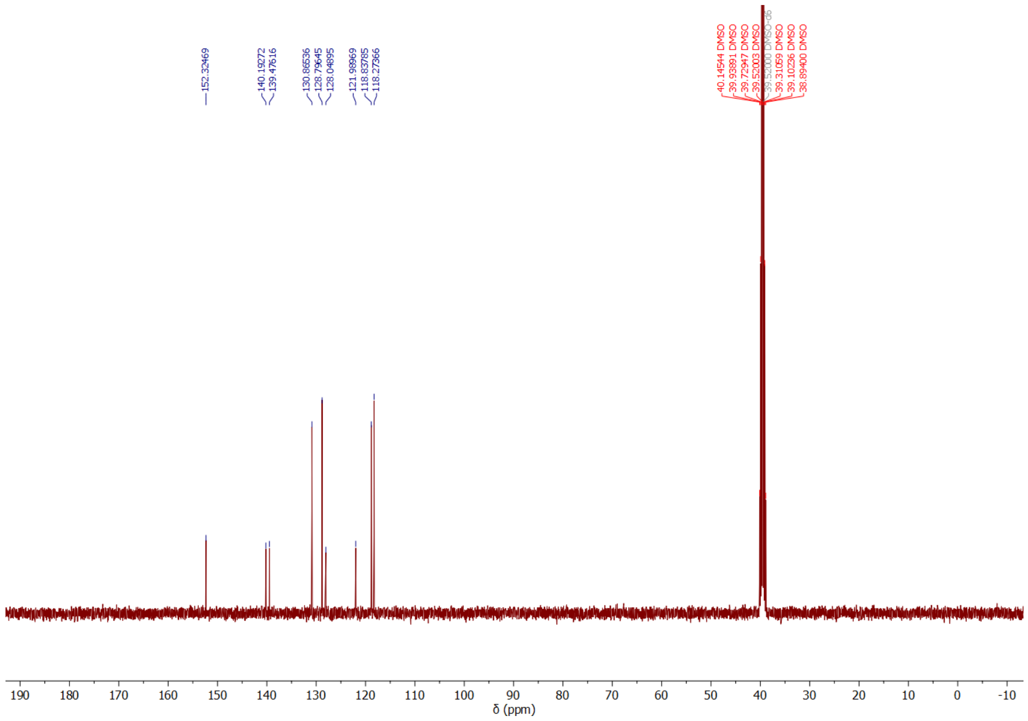


**Figure S25**. $^{13}$C NMR spectrum of **analogue 4** (100 MHz, DMSO-$d_6$, at 25 °C).

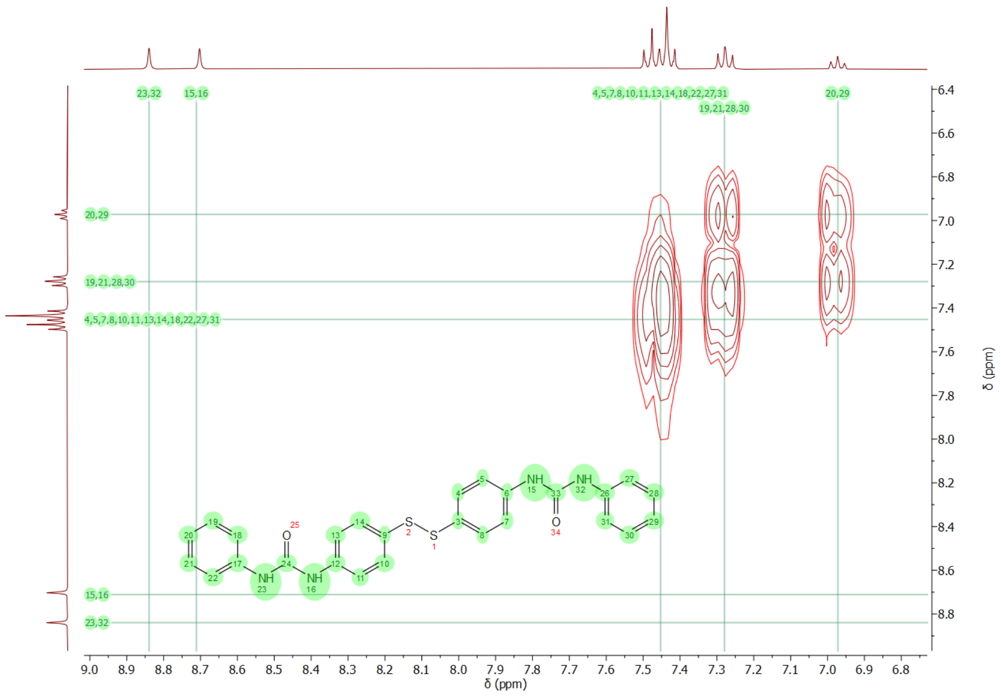

**Figure S26**. 2D COSY ($^1H$-$^1H$) NMR spectrum of **analogue 4** (400 MHz, DMSO-$d_6$, at 25 °C).

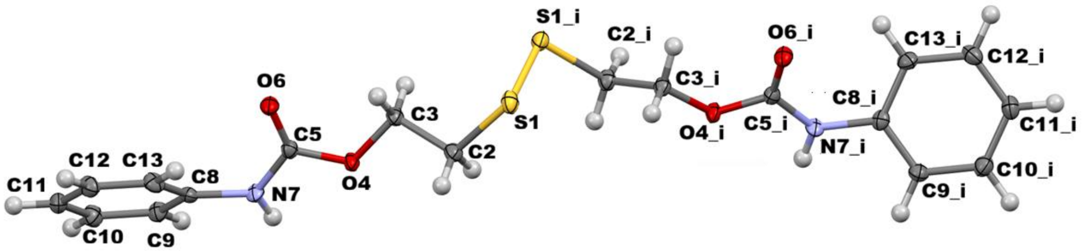


**Figure S27**. The asymmetric unit of **analogue 1** was determined from single-crystal X-ray diffraction analysis. One molecule of **analogue 1** (Symmetry Code (i): 2 – *x*, 2 – *y*, *z*). There are two molecules in the unit cell. Thermal ellipsoids are drawn at 50% probability.

**Table S2**. Crystallographic details for **analogue 1**.

| | |
|---|---|
| Formula | $C_{18}$ $H_{20}$ $N_2$ $O_4$ $S_2$ |
| $M_r$ | 392.48 |
| Crystal system | orthorhombic |
| Space group | *P n n* 2 |
| Z | 2 |
| *a* /Å | 14.0482(4) |
| *b*/Å | 12.4346(3) |
| *c* /Å | 5.13432(16) |
| *V* / Å$^3$ | 896.88(5) |
| $D_{calc}$ / g cm$^{-3}$ | 1.453 |
| Crystal habit | Colourless block |
| Crystal dimensions /mm | 0.020 × 0.041× 0.186 |
| Radiation | Cu $K_\alpha$ (1.54184 Å) |
| T /K | 100 |
| *μ* /mm$^{-1}$ | 2.929 |
| *R*(*F*), *Rw*(*F*) | 2.27, 5.50 |
| CCDC cif deposition number | CCDC  2517784 |

**Table S3**. Selected bond lengths (Å) and angles (°) in **analogue 1**.

| | | | |
|---|---|---|---|
| S(1) – S(1)[i] | 2.0424(8) | S(1)[i] – S(1) – C(2) | 105.02(6) |
| S(1) – C(2) | 1.8164(18) | S(1) – C(2) – C(3) | 111.72(12) |
| C(2) – C(3) | 1.515(2) | C(2) – C(3) – O(4) | 106.66(13) |
| C(3) – O(4) | 1.446(2) | C(3) – O(4) – C(5) | 116.30(13) |
| O(4) – C(5) | 1.351(2) | O(4) – C(5) – O(6) | 124.46(15) |
| C(5) – O(6) | 1.214(2) | O(4) – C(5) – N(7) | 109.39(15) |
| C(5) – N(7) | 1.357(2) | O(6) – C(5) – N(7) | 126.14(15) |
| N(7) – C(8) | 1.4192(18) | C(5) – N(7) – C(8) | 124.39(15) |
| C(8) – C(9) | 1.388(2) | N(7) – C(8) – C(9) | 118.71(15) |
| C(8) – C(13) | 1.392(3) | N(7) – C(8) – C(13) | 121.25(15) |
| C(9) – C(10) | 1.392(2) | C(9) – C(8) – C(13) | 119.99(14) |
| C(10) – C(11) | 1.381(3) | C(8) – C(9) – C(10) | 119.66(17) |
| C(11) – C(12) | 1.387(3) | C(9) – C(10) – C(11) | 120.65(17) |
| C(12) – C(13) | 1.389(2) | C(10) – C(11) – C(12) | 119.49(14) |
| | | C(11) – C(12) – C(13) | 120.52(16) |
| | | C(8) – C(13) – C(12) | 119.69(16) |

Symmetry Code: (i): 2 – *x*, 2 – *y*, *z*).

**Table S4**. Hydrogen-bond and close-contact geometry (Å, °) in **analogue 1**.

| *D* – H•••A | *D* – H | H•••*A* | *D* •••*A* | *D* – H•••*A* |
|---|---|---|---|---|
| N(7) – H(71)•••O(6)[ii] | 0.833(9) | 2.149(11) | 2.953(2) | 162.2(19) |
| C(12) – H(121)•••O(4)[iii] | 0.95 | 2.56 | 3.482(2) | 164 |

Symmetry Codes: (ii) *x* , *y*, *z* + 1; (iii) –*x* + 3/2, *y* – 1/2, *z* – ½.

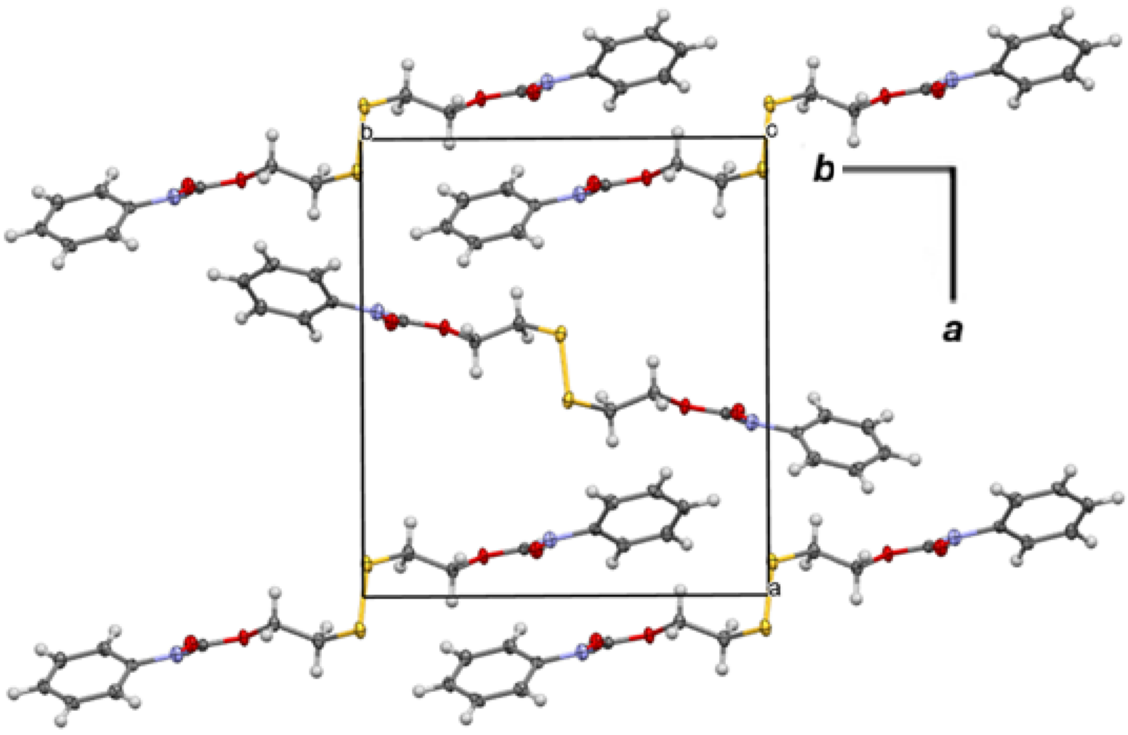


**Figure S28**. Packing of the molecules of **analogue 1**. view along the *c* axis showing the stacking.

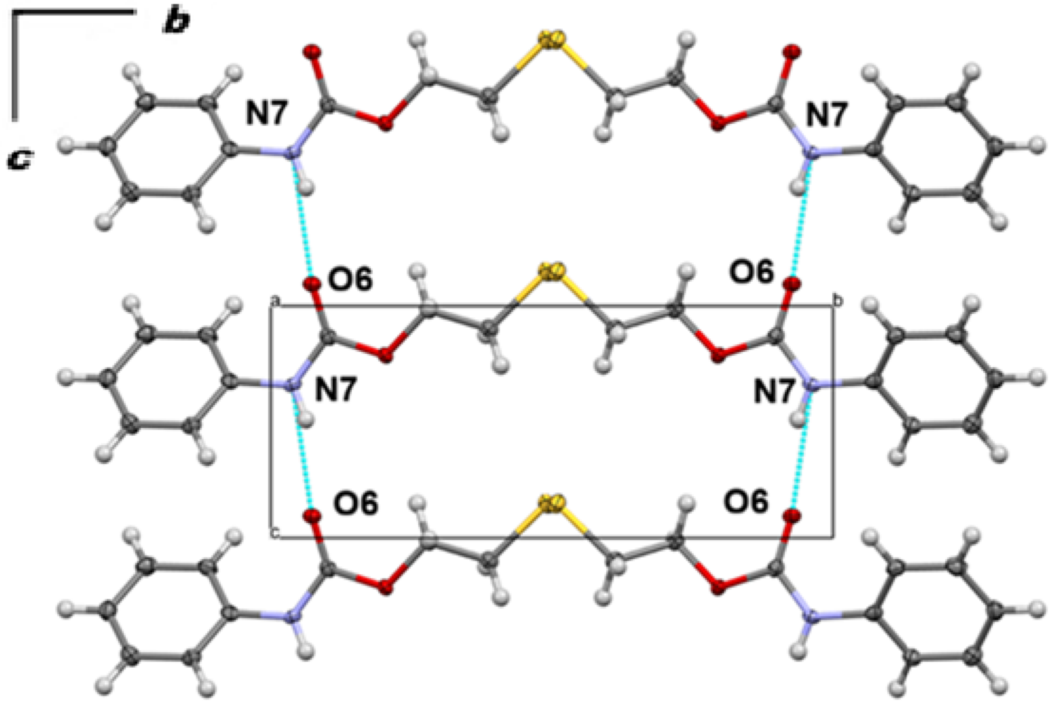


**Figure S29**. Packing of the molecules of **analogue 1**, view along the *a* axis showing the N-H…O hydrogen-bonding interactions (pale-blue lines, N(7) – H(71)…O(6)[ii], 2.953(2) Å) between adjacent molecules to form layers lying in the *bc* plane. Symmetry Code: (ii) *x* , *y*, *z* + 1.

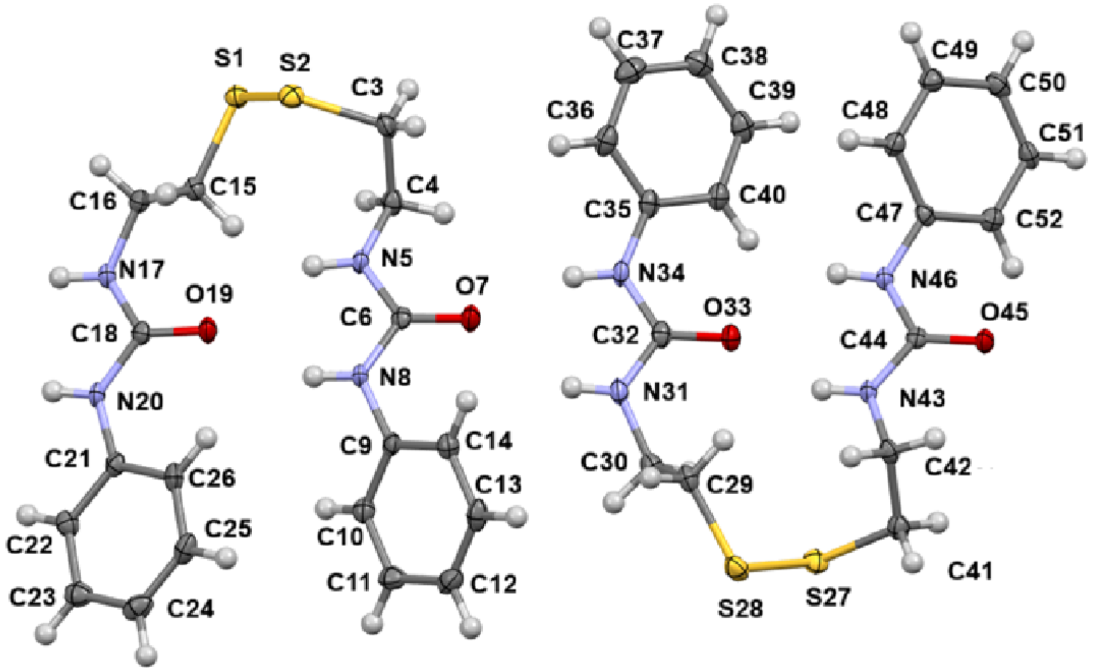


**Figure S30**. The asymmetric unit of **analogue 3** was determined from single-crystal X-ray diffraction analysis. There are two molecules in the asymmetric unit and eight in the unit cell. Thermal ellipsoids are drawn at 50% probability.

**Table S5**. Crystallographic details for **analogue 3**.

| | |
|---|---|
| Formula | $C_{18}$ $H_{20}$ $N_2$ $O_4$ $S_2$ |
| $M_r$ | 390.52 |
| Crystal system | orthorhombic |
| Space group | $P\ n\ 2_1\ a$ |
| Z | 8 |
| *a* /Å | 8.91998(8) |
| *b*/Å | 22.53911(19) |
| *c* /Å | 18.57490(15) |
| *V* / Å$^3$ | 3734.45(6) |
| $D_{calc}$ / g cm$^{-3}$ | 1.389 |
| Crystal habit | Colourless plate |
| Crystal dimensions /mm | 0.027 × 0.097× 0.109 |
| Radiation | Cu $K_\alpha$ (1.54184 Å) |
| T /K | 100 |
| $\mu$ /mm$^{-1}$ | 2.758 |
| *R*(*F*), *Rw*(*F*) | 4.39, 11.04 |
| CCDC cif deposition number | CCDC 2517785 |

**Table S6**. Selected bond lengths (Å) and angles (°) in **analogue 3**.

| | | | |
|---|---|---|---|
| S(1) – S(2) | 2.0452(10) | S(2) – S(1) – C(15) | 103.72(10) |
| S(1) – C(15) | 1.823(3) | S(1) – S(2) – C(3) | 104.61(10) |
| S(2) – C(3) | 1.823(3) | S(2) – C(3) – C(4) | 114.27(19) |
| C(3) – C(4) | 1.522(4) | C(3) – C(4) – N(5) | 113.8(2) |
| C(4) – N(5) | 1.454(4) | C(4) – N(5) – C(6) | 123.0(2) |
| N(5) – C(6) | 1.347(4) | N(5) – C(6) – O(7) | 123.6(3) |
| C(6) – O(7) | 1.238(4) | N(5) – C(6) – N(8) | 114.4(2) |
| C(6) – N(8) | 1.372(4) | O(7) – C(6) – N(8) | 122.0(3) |
| N(8) – C(9) | 1.417(4) | C(6) – N(8) – C(9) | 123.4(2) |
| C(9) – C(10) | 1.387(4) | N(8) – C(9) – C(10) | 118.9(2) |
| C(9) – C(14) | 1.393(4) | N(8) – C(9) – C(14) | 121.2(3) |
| C(10) – C(11) | 1.383(4) | C(10) – C(9) – C(14) | 119.9(3) |
| C(11) – C(12) | 1.392(4) | C(9) – C(10) – C(11) | 120.3(3) |
| C(12) – C(13) | 1.383(5) | C(10) – C(11) – C(12) | 120.4(3) |
| C(13) – C(14) | 1.393(4) | C(11) – C(12) – C(13) | 119.1(3) |
| C(15) – C(16) | 1.519(4) | C(12) – C(13) – C(14) | 121.0(3) |
| C(16) – N(17) | 1.455(4) | C(9) – C(14) – C(13) | 119.2(3) |
| N(17) – C(18) | 1.352(4) | S(1) – C(15) – C(16) | 111.94(19) |
| C(18) – O(19) | 1.243(3) | C(15) – C(16) – N(17) | 112.3(2) |
| C(18) – N(20) | 1.369(4) | C(16) – N(17) – C(18) | 120.6(2) |
| N(20) – C(21) | 1.400(4) | N(17) – C(18) – O(19) | 122.5(3) |
| C(21) – C(22) | 1.404(4) | N(17) – C(18) – N(20) | 113.7(2) |
| C(21) – C(26) | 1.392(4) | O(19) – C(18) – N(20) | 123.8(3) |
| C(22) – C(23) | 1.381(4) | C(18) – N(20) – C(21) | 128.9(2) |
| C(23) – C(24) | 1.388(5) | N(20) – C(21) – C(22) | 116.2(3) |
| C(24) – C(25) | 1.381(5) | N(20) – C(21) – C(26) | 124.6(3) |

| | | | |
|---|---|---|---|
| C(25) – C(26) | 1.388(4) | C(22) – C(21) – C(26) | 119.1(3) |
| S(27) – S(28) | 2.0428(10) | C(21) – C(22) – C(23) | 120.8(3) |
| S(27) – C(41) | 1.821(3) | C(22) – C(23) – C(24) | 119.9(3) |
| S(28) – C(29) | 1.822(3) | C(23) – C(24) – C(25) | 119.3(3) |
| C(29) – C(30) | 1.519(4) | C(24) – C(25) – C(26) | 121.6(3) |
| C(30) – N(31) | 1.462(4) | C(21) – C(26) – C(25) | 119.2(3) |
| N(31) – C(32) | 1.352(4) | S(28) – S(27) – C(41) | 104.16(10) |
| C(32) – O(33) | 1.237(4) | S(27) – S(28) – C(29) | 104.25(10) |
| C(32) – N(34) | 1.374(4) | S(28) – C(29) – C(30) | 111.8(2) |
| N(34) – C(35) | 1.409(4) | C(29) – C(30) – N(31) | 113.0(2) |
| C(35) – C(36) | 1.391(4) | C(30) – N(31) – C(32) | 119.8(2) |
| C(35) – C(40) | 1.397(4) | N(31) – C(32) – O(33) | 122.4(3) |
| C(36) – C(37) | 1.388(5) | N(31) – C(32) – N(34) | 114.0(2) |
| C(37) – C(38) | 1.383(5) | O(33) – C(32) – N(34) | 123.6(3) |
| C(38) – C(39) | 1.390(5) | C(32) – N(34) – C(35) | 127.0(2) |
| C(39) – C(40) | 1.386(4) | N(34) – C(35) – C(36) | 117.9(3) |
| C(41) – C(42) | 1.522(4) | N(34) – C(35) – C(40) | 122.7(3) |
| C(42) – N(43) | 1.447(4) | C(36) – C(35) – C(40) | 119.4(3) |
| N(43) – C(44) | 1.352(4) | C(35) – C(36) – C(37) | 120.6(3) |
| C(44) – O(45) | 1.240(3) | C(36) – C(37) – C(38) | 120.1(3) |
| C(44) – N(46) | 1.370(4) | C(37) – C(38) – C(39) | 119.5(3) |
| N(46) – C(47) | 1.411(4) | C(38) – C(39) – C(40) | 120.9(3) |
| C(47) – C(48) | 1.403(4) | C(35) – C(40) – C(39) | 119.5(3) |
| C(47) – C(52) | 1.389(4) | S(27) – C(41) – C(42) | 115.04(19) |
| C(48) – C(49) | 1.386(4) | C(41) – C(42) – N(43) | 114.0(2) |

| | | | |
|---|---|---|---|
| C(49) – C(50) | 1.378(4) | C(42) – N(43) – C(44) | 122.6(2) |
| C(50) – C(51) | 1.389(4) | N(43) – C(44) – O(45) | 122.7(2) |
| C(51) – C(52) | 1.390(4) | N(43) – C(44) – N(46) | 114.6(2) |
| | | O(45) – C(44) – N(46) | 122.7(2) |
| | | C(44) – N(46) – C(47) | 123.1(2) |
| | | N(46) – C(47) – C(48) | 118.7(2) |
| | | N(46) – C(47) – C(52) | 122.1(3) |
| | | C(48) – C(47) – C(52) | 119.2(3) |
| | | C(47) – C(48) – C(49) | 120.1(3) |
| | | C(48) – C(49) – C(50) | 120.6(3) |
| | | C(49) – C(50) – C(51) | 119.6(3) |
| | | C(50) – C(51) – C(52) | 120.5(3) |
| | | C(47) – C(52) – C(51) | 120.1(3) |

**Table S7**. Hydrogen-bond and close-contact geometry (Å, °) in **analogue 3**.

| *D* – H•••A | *D* – H | H•••*A* | *D* •••*A* | *D* – H•••*A* |
|---|---|---|---|---|
| N(5) – H(51)•••O(19) | 0.848(5) | 2.245(17) | 3.027(4) | 153(3) |
| N(8) – H(81)•••O(19) | 0.850 (5) | 2.102(16) | 2.898(4) | 156(3) |
| N(17) – H(171)•••O(45)$^{i}$ | 0.851(5) | 2.29(2) | 3.037(4) | 147(3) |
| N(20) – H(201)•••O(45)$^{i}$ | 0.849(5) | 2.012(13) | 2.830(4) | 162(3) |
| N(31) – H(311)•••O(7) | 0.849 (5) | 2.26(2) | 3.017(4) | 148(3) |
| N(34) – H(341)•••O(7) | 0.850(5) | 1.997(13) | 2.819(4) | 163(4) |
| N(43) – H(431)•••O(33) | 0.849(5) | 2.150(19) | 2.917(4) | 150(3) |
| N(46) – H(461)•••O(33) | 0.849(5) | 2.23(2) | 2.952(4) | 143(3) |

Symmetry Codes: (i) *x*, *y*, *z* – 1.

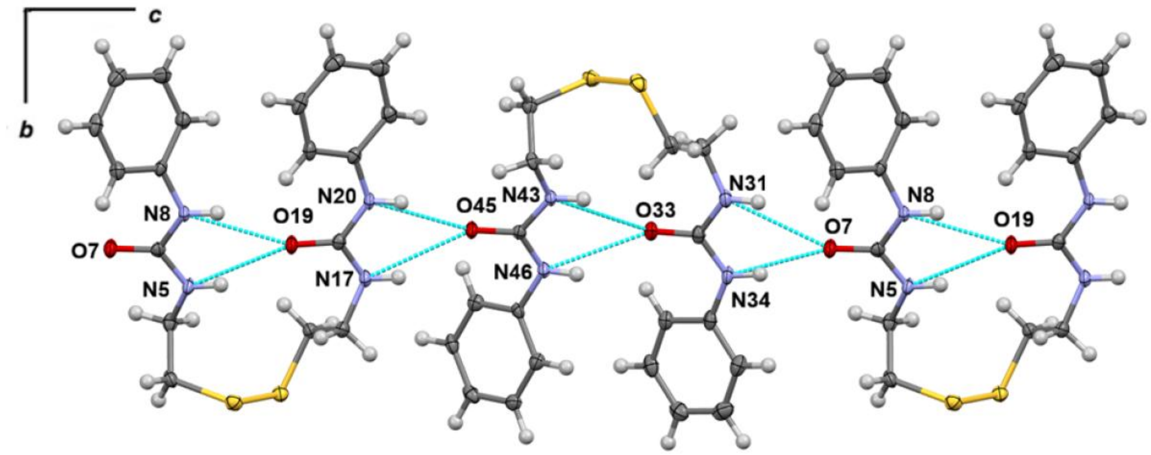


**Figure S31**. Packing of the molecules of compound **analogue 3**. view along the *a* axis showing the bifurcated N-H…O hydrogen-bonding interactions (pale-blue lines) within and between the two distinct molecules in the asymmetric unit leading to chains running in the *c* direction.

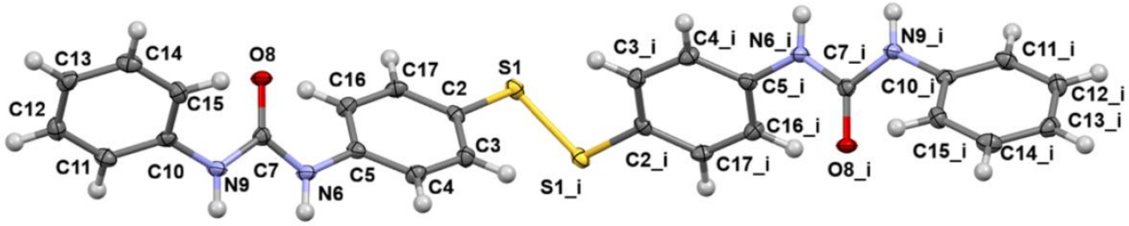


**Figure S32**. The asymmetric unit of **analogue 4** was determined from single-crystal X-ray diffraction analysis. one molecule of **analogue 4** (Symmetry Code (i): –*x* + 1, *y*, – *z* + 3/2). There are four molecules in the unit cell. Thermal ellipsoids are drawn at 50% probability.

**Table S8**. Crystallographic details for **analogue 4**.

| | |
|---|---|
| Formula | $C_{26} H_{22} N_4 O_2 S_2$ |
| $M_r$ | 486.60 |
| Crystal system | orthorhombic |
| Space group | *P b c n* |
| Z | 4 |
| *a* /Å | 4.65056(6) |
| *b*/Å | 9.96593(18) |
| *c* /Å | 47.9982(7) |
| *V* / Å$^3$ | 2224.58(6) |
| $D_{calc}$ / g cm$^{-3}$ | 1.453 |
| Crystal habit | Colourless plate |
| Crystal dimensions /mm | 0.024 × 0.032× 0.145 |
| Radiation | Cu $K_\alpha$ (1.54184 Å) |
| T /K | 100 |
| $\mu$ /mm$^{-1}$ | 2.444 |
| *R*(*F*), *Rw*(*F*) | 5.03, 8.95 |
| CCDC cif deposition number | CCDC 2517786 |

**Table S9**. Selected bond lengths (Å) and angles (°) in **analogue 4**.

| | | | |
|---|---|---|---|
| S(1) – S(1)$^i$ | 2.0313(12) | S(1)$^i$ – S(1) – C(2) | 105.22(8) |
| S(1) – C(2) | 1.785(2) | S(1) – C(2) – C(3) | 124.29(18) |
| C(2) – C(3) | 1.385(3) | S(1) – C(2) – C(17) | 115.87(18) |
| C(2) – C(17) | 1.395(3) | C(3) – C(2) – C(17) | 119.8(2) |
| C(3) – C(4) | 1.387(3) | C(2) – C(3) – C(4) | 119.5(2) |
| C(4) – C(5) | 1.392(3) | C(3) – C(4) – C(5) | 120.9(2) |
| C(5) – N(6) | 1.425(3) | C(4) – C(5) – N(6) | 118.4(2) |
| C(5) – C(16) | 1.391(3) | C(4) – C(5) – C(16) | 119.3(2) |
| N(6) – C(7) | 1.357(3) | N(6) – C(5) – C(16) | 122.2(2) |
| C(7) – O(8) | 1.238(3) | C(5) – N(6) – C(7) | 124.3(2) |
| C(7) – N(9) | 1.367(3) | N(6) – C(7) – O(8) | 123.3(2) |
| N(9) – C(10) | 1.423(3) | N(6) – C(7) – N(9) | 114.1(2) |

| | | | |
|---|---|---|---|
| C(10) – C(11) | 1.384(3) | O(8) – C(7) – N(9) | 122.6(2) |
| C(10) – C(15) | 1.389(3) | C(7) – N(9) – C(10) | 122.8(2) |
| C(11) – C(12) | 1.386(3) | N(9) – C(10) – C(11) | 119.8(2) |
| C(12) – C(13) | 1.386(4) | N(9) – C(10) – C(15) | 120.7(2) |
| C(13) – C(14) | 1.388(3) | C(11) – C(10) – C(15) | 119.5(2) |
| C(14) – C(15) | 1.390(3) | C(10) – C(11) – C(12) | 119.9(2) |
| C(16) – C(17) | 1.381(3) | C(11) – C(12) – C(13) | 120.9(2) |
| | | C(12) – C(13) – C(14) | 119.3(2) |
| | | C(13) – C(14) – C(15) | 119.9(2) |
| | | C(10) – C(15) – C(14) | 120.5(2) |
| | | | |
| | | C(5) – C(16) – C(17) | 119.9(2) |
| | | C(2) – C(17) – C(16) | 120.5(2) |

**Table S10**. Hydrogen-bond and close-contact geometry (Å, °) in **analogue 4**.

| *D* – H•••A | *D* – H | H•••*A* | *D* •••*A* | *D* – H•••*A* |
|---|---|---|---|---|
| N(6) – H(61)•••O(8)$^{ii}$ | 0.854(10) | 2.070(15) | 2.875(3) | 157(3) |
| N(9) – H(91)•••N(37)$^{ii}$ | 0.850(10) | 2.165(16) | 2.937(3) | 151 (3) |

Symmetry Code: (ii) *x* + 1, *y*, *z*.

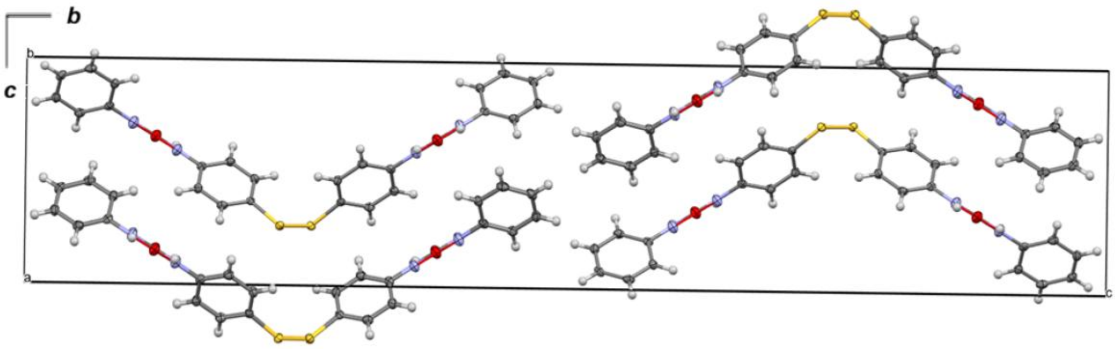


**Figure S33**. Packing of the molecules of compound **analogue 4**, view along the *a* axis showing the stacking of the molecules

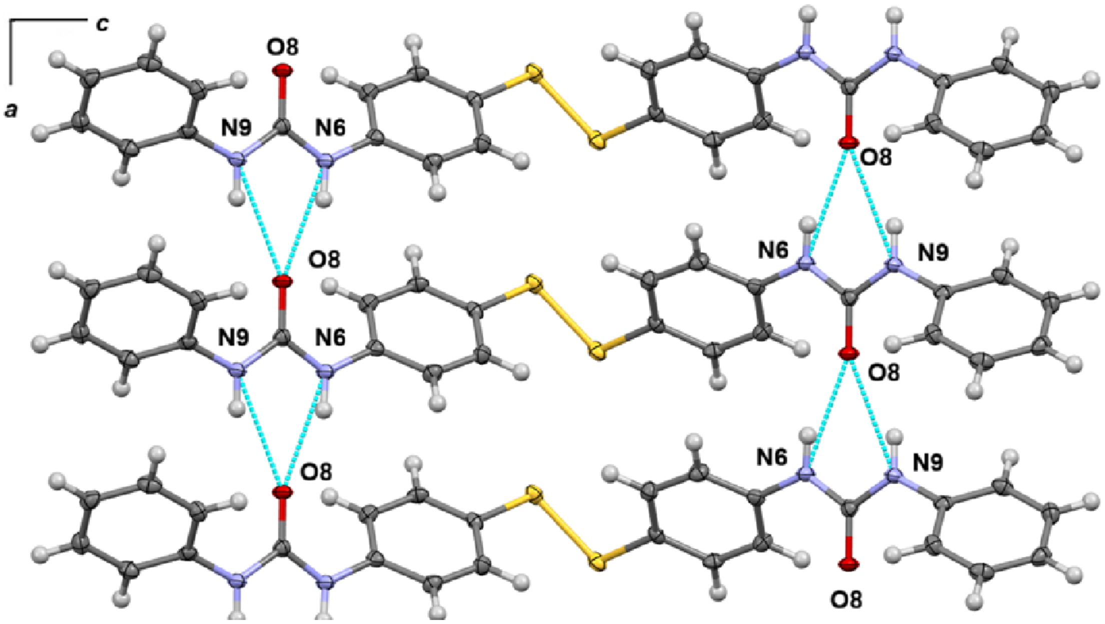


**Figure S34**. Packing of the molecules of compound **analogue 4**, view along the *b* axis showing the bifurcated N-H…O hydrogen-bonding interactions (pale-blue lines) between adjacent molecules (N(6) – H(61)…O(8)$^{ii}$, 2.875(3) Å and N(9) – H(91)…O(8)$^{ii}$, 2.937(3) Å).

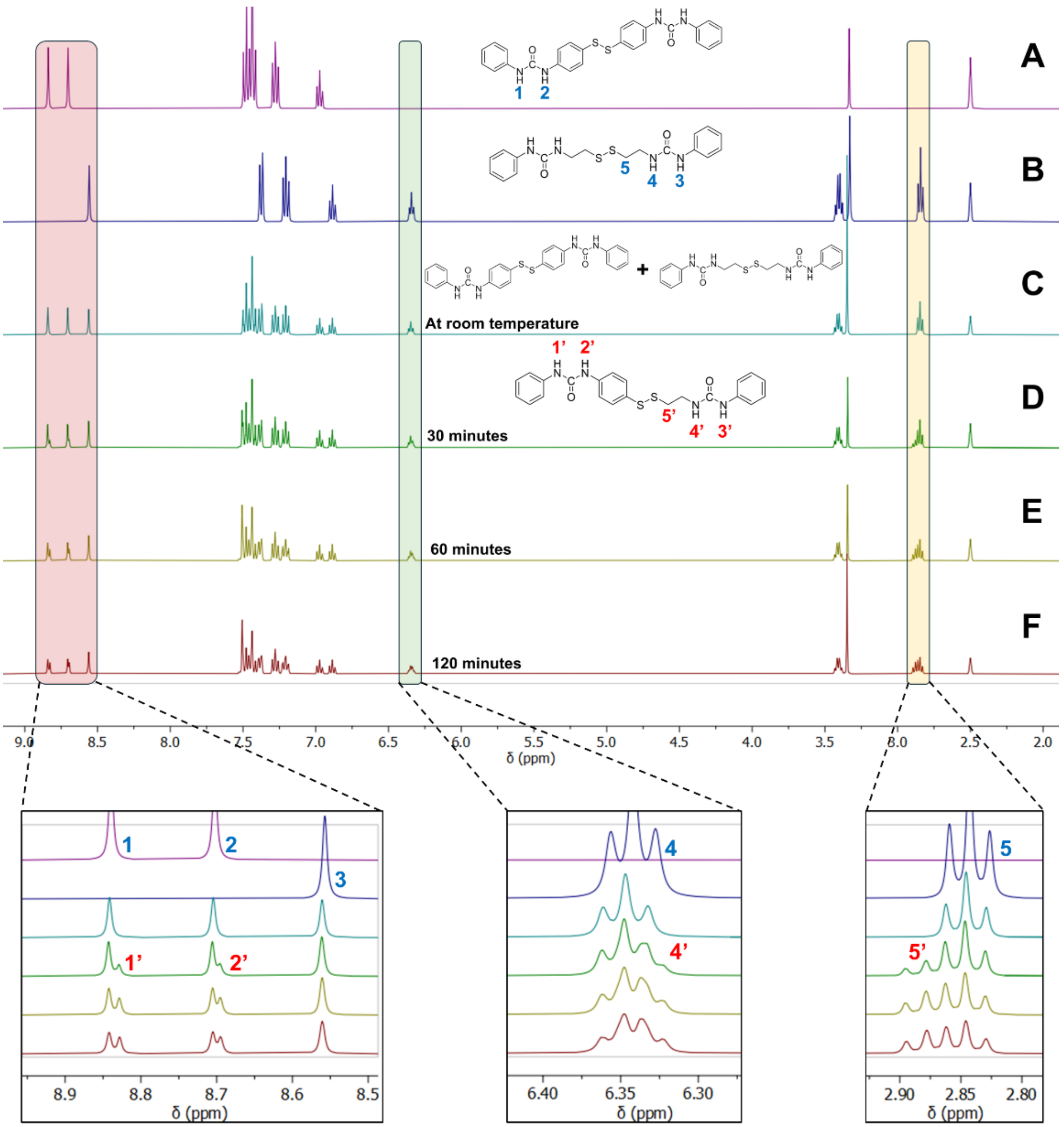


Disulfide chain exchange (%) = $\frac{\int 1'}{\int 1} * 100$

**Figure S35**. $^{1}$H NMR spectra of (A) **analogue 4**, (B) **analogue 3** (C) mixture of **analogue 3** and **4** at room temperature, and (D,E) mixture of **analogue 3** and **4** after heating to 80 °C for 30, 60 and 120 minutes, respectively, in DMSO-$d_6$.

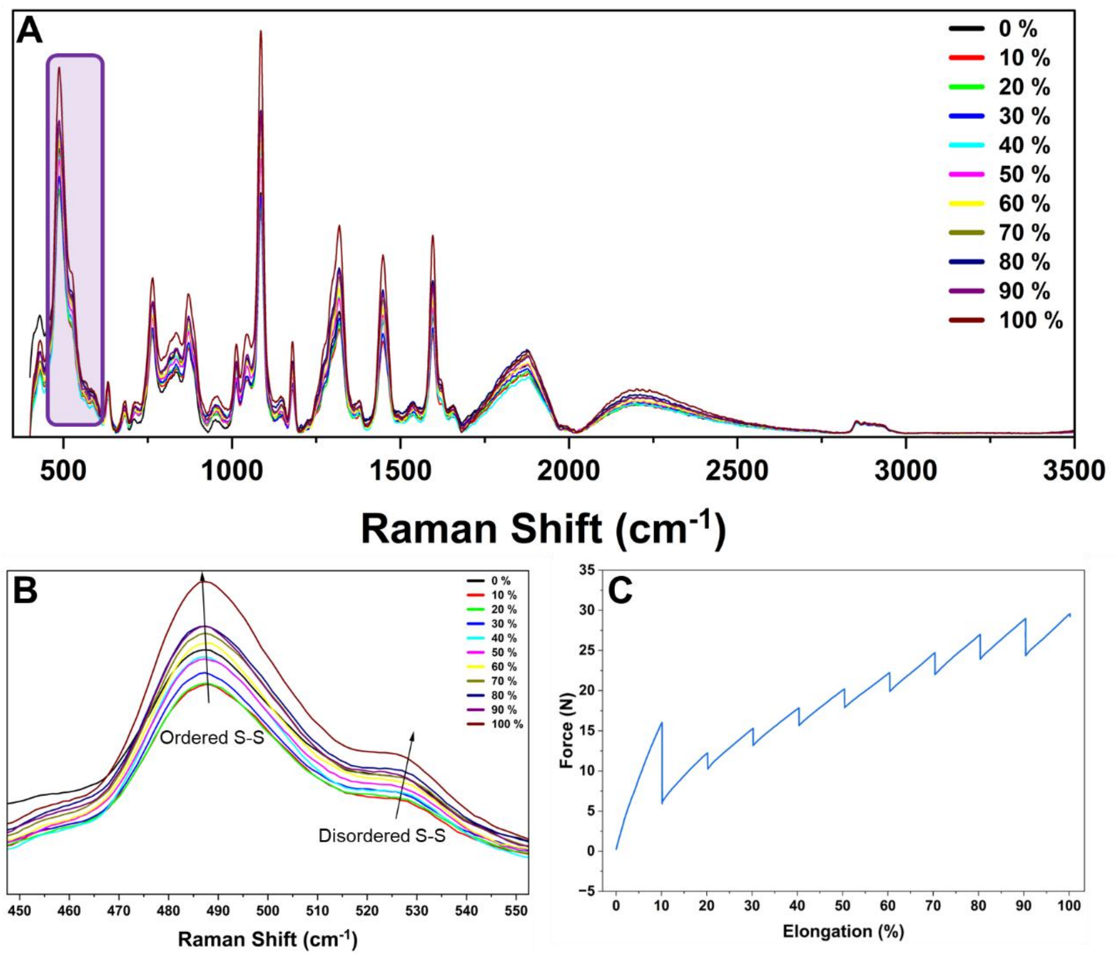


**Figure S36**. (A) Raman spectra of **CEPU4** as a function of strain, (B) ordered/disordered disulfide signals, and (C) load force development during stretching from 0% to a maximum of 100% at 10 % interval.

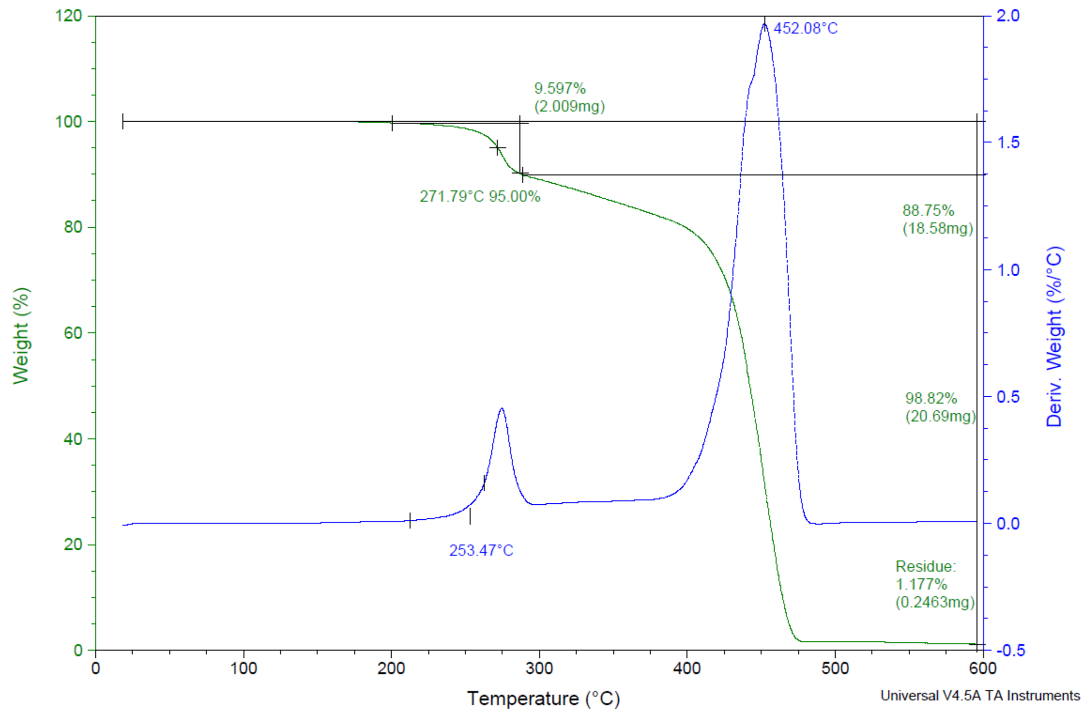


**Figure S37**. TGA thermogram of **CEPU1** at 10 °C min$^{-1}$ under nitrogen.

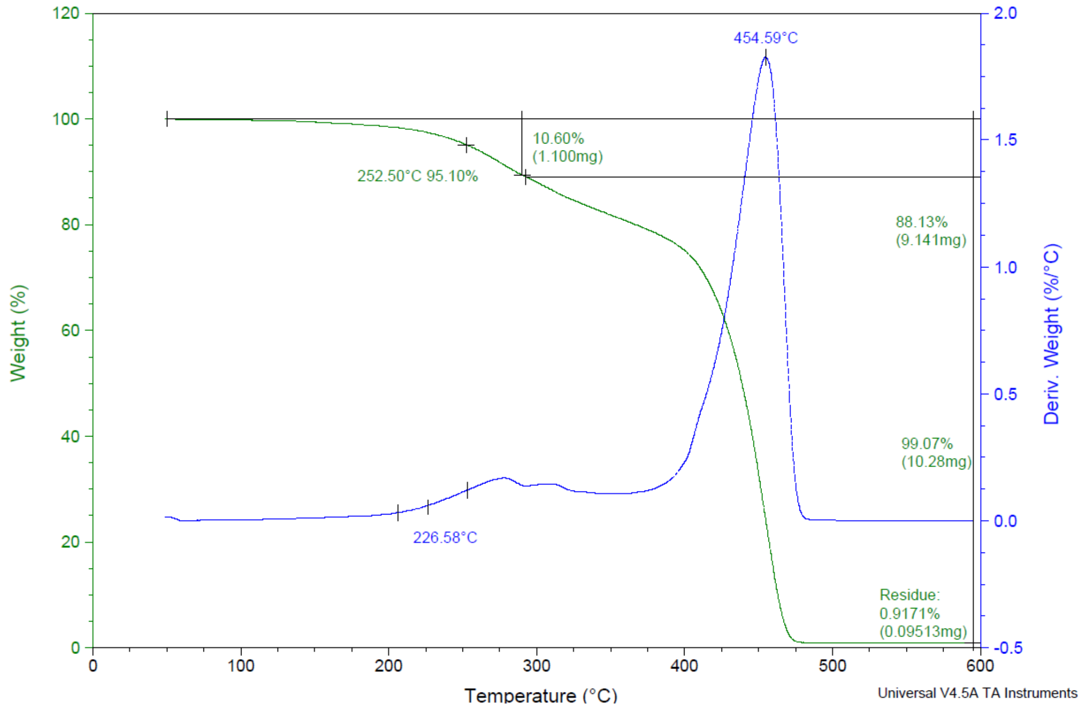


**Figure S38**. TGA thermogram of **CEPU2** at 10 °C min$^{-1}$ under nitrogen.

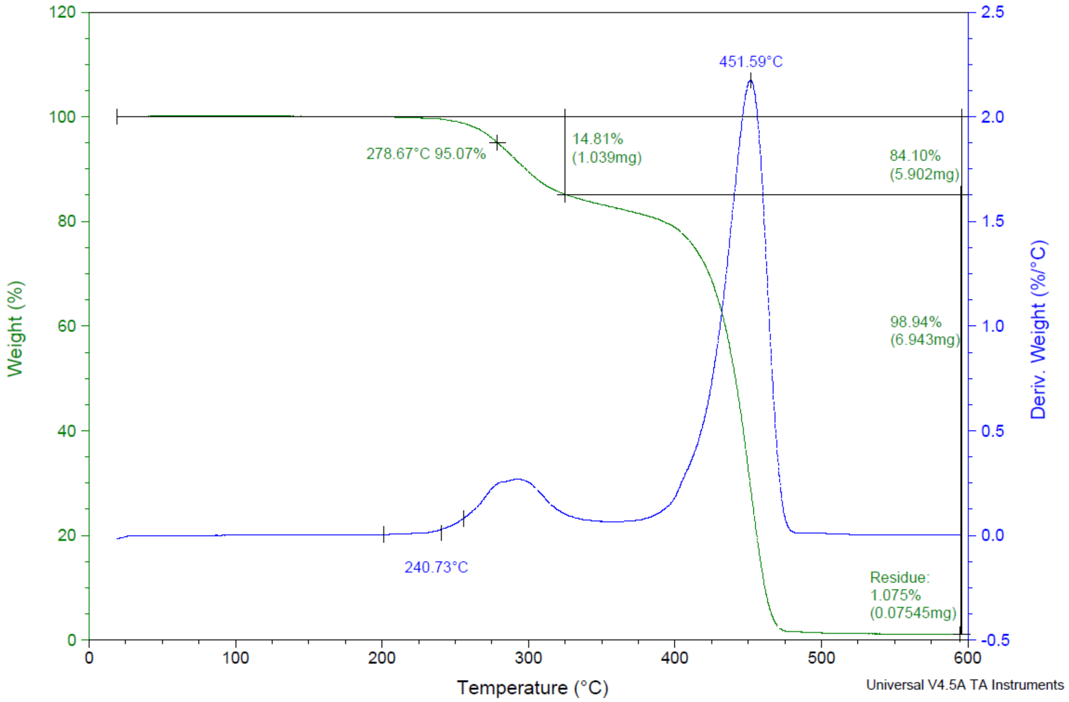


**Figure S39**. TGA thermogram of **CEPU3** at 10 °C min$^{-1}$ under nitrogen.

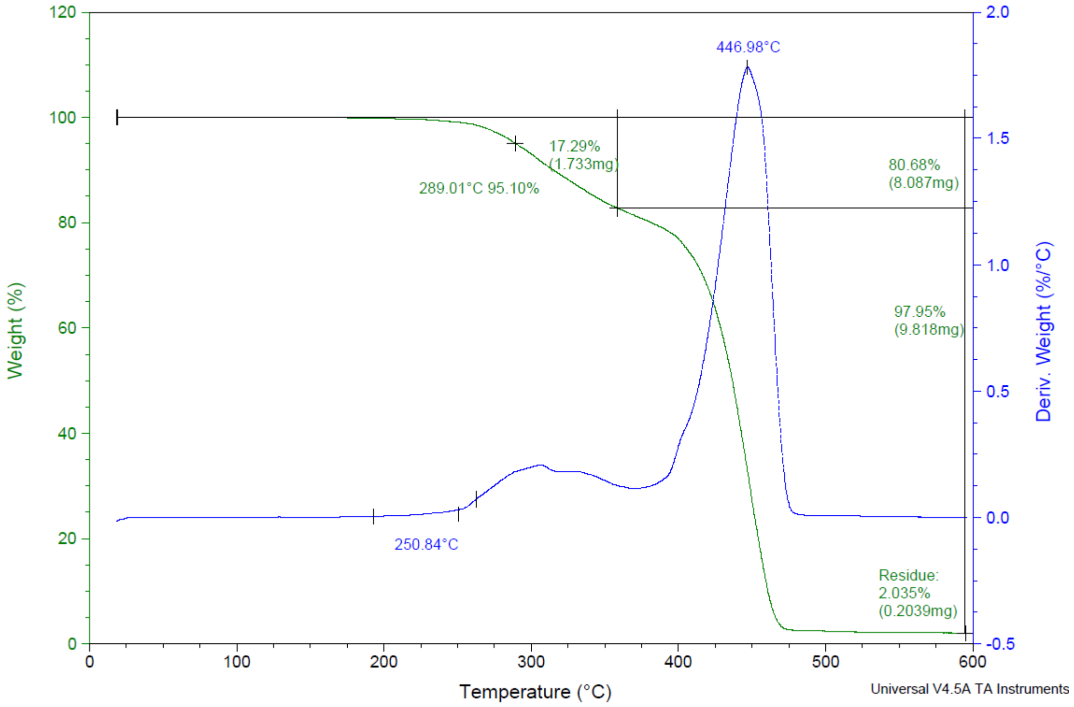


**Figure S40**. TGA thermogram of **CEPU4** at 10 °C min$^{-1}$ under nitrogen.

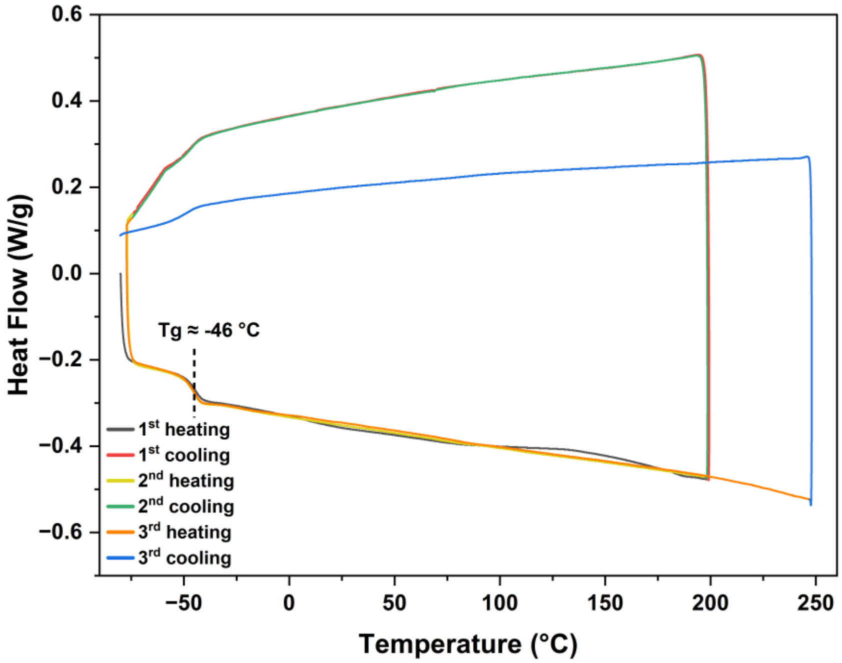


**Figure S41**. DSC thermogram of **CEPU1** shows the 1st, 2nd, and 3rd heating and cooling cycles from -80 °C to 200 °C at 10 °C min$^{-1}$, 1st heating after isotherm at -90 °C for 60 min, and 3rd heating up to 250 °C.

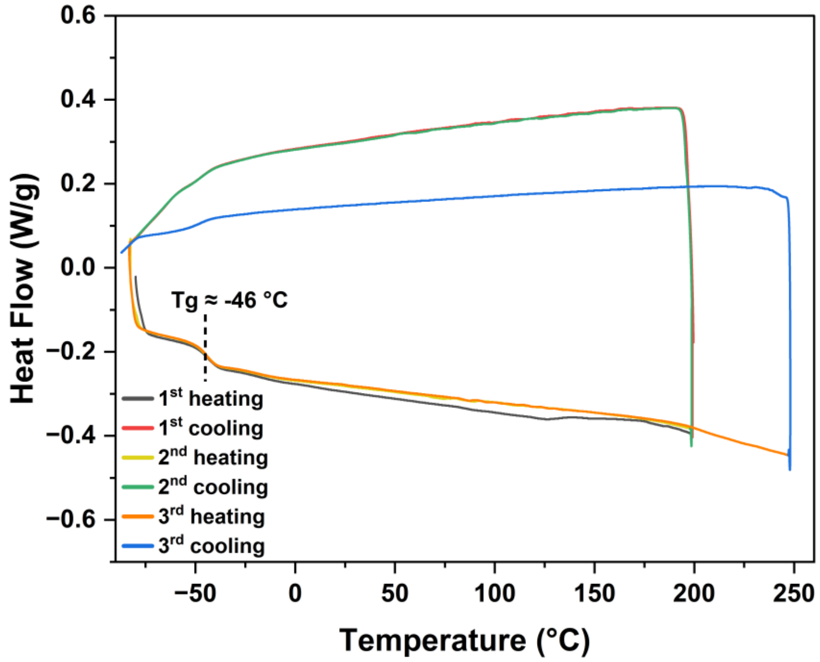


**Figure S42**. DSC thermogram of **CEPU2** shows the 1st, 2nd, and 3rd heating and cooling cycles from -80 °C to 200 °C at 10 °C min$^{-1}$, 1st heating after isotherm at -90 °C for 60 min, and 3rd heating up to 250 °C.

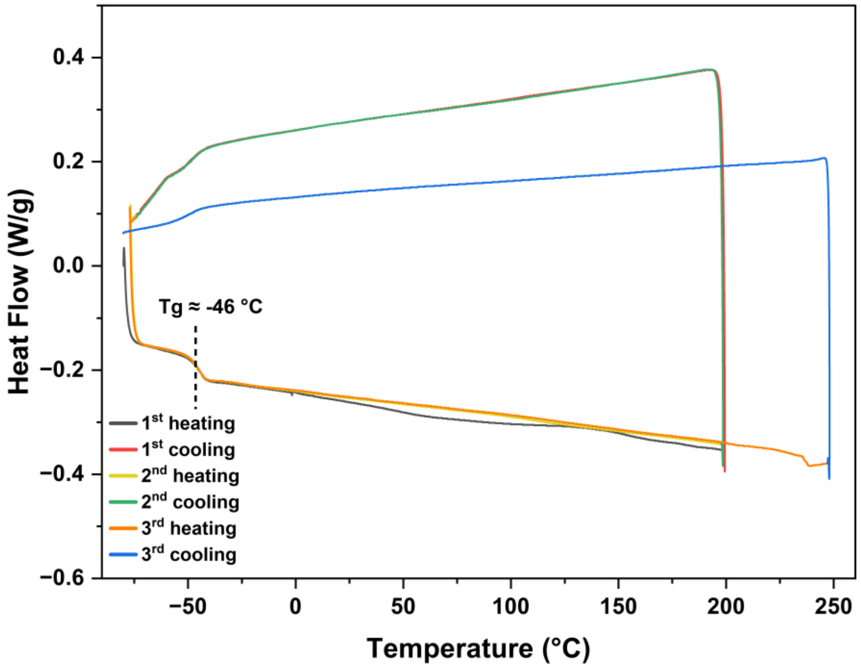


**Figure S43**. DSC thermogram of **CEPU3** shows the 1st, 2nd, and 3rd heating and cooling cycles from -80 °C to 200 °C at 10 °C min$^{-1}$, 1st heating after isotherm at -90 °C for 60 min, and 3rd heating up to 250 °C.

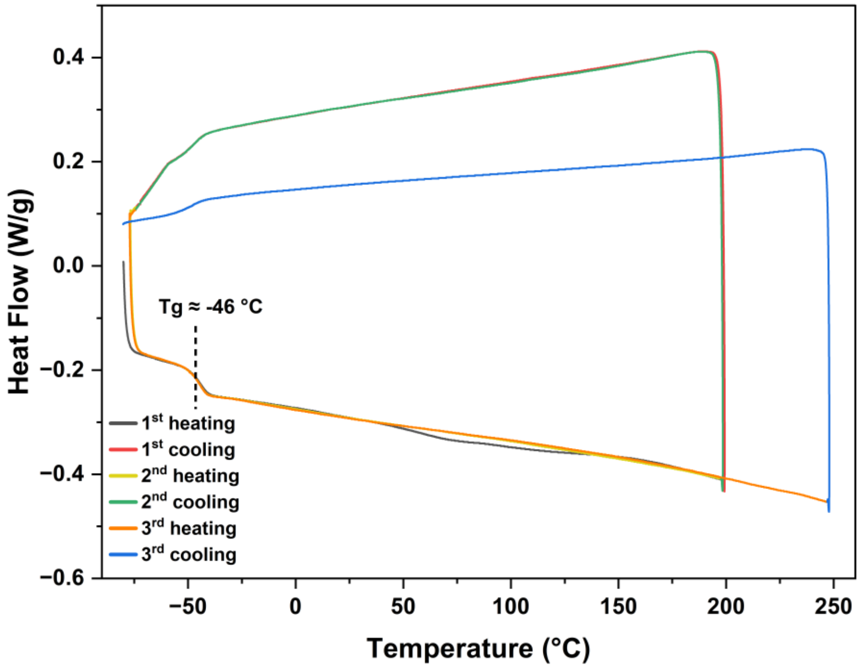


**Figure S44**. DSC thermogram of **CEPU4** shows the 1st, 2nd, and 3rd heating and cooling cycles from -80 °C to 200 °C at 10 °C min$^{-1}$, 1st heating after isotherm at -90 °C for 60 min, and 3rd heating up to 250 °C.

**Table S11**: GPC molecular weight, polydispersity data and thermal properties for **CEPU1-CEPU4**.

| CEPUs (Yield %) | $M_n$ (g mol$^{-1}$) | $M_w$ (g mol$^{-1}$) | Đ | $T_d$ 5 % (°C)[a] | $T_g$ (°C) | $T_m$ (°C)[b] | Enthalpy (J/g) |
|---|---|---|---|---|---|---|---|
| **CEPU1** (92%) | 17400 | 45500 | 2.61 | 272 | -46.03 | 26.6, 82.4 | 0.18, 0.62 |
| **CEPU2** (90%) | 15600 | 49100 | 3.15 | 253 | -46.00 | 9.5 | 0.044 |
| **CEPU3** (94%) | 25800 | 217900 | 8.45 | 279 | -46.16 | 41.5, 155.6 | 0.93, 0.10 |
| **CEPU4** (95%) | 11400 | 53500 | 4.69 | 289 | -46.46 | 72.6 | 0.24 |

[a] Onset of 5% thermal decomposition temperature, [b] First heating run 10 °C min$^{-1}$

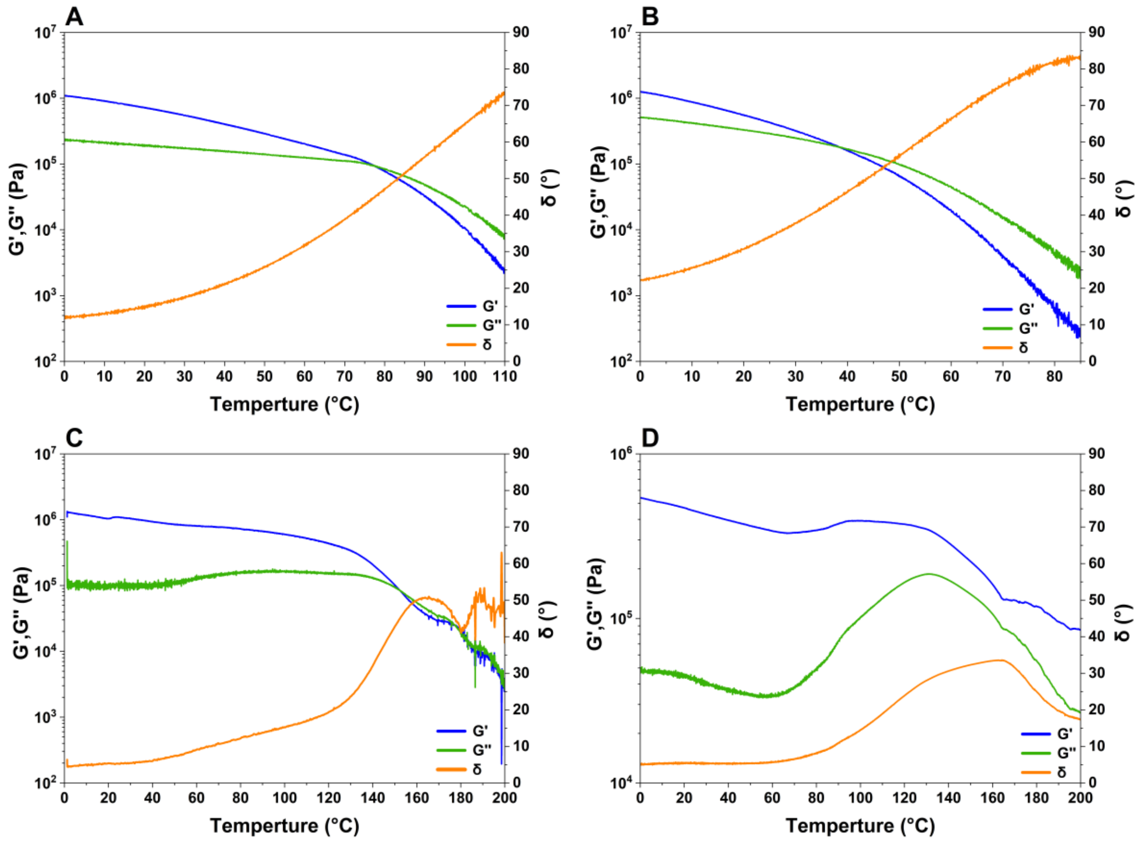


**Figure S45**. Temperature sweep analysis of **CEPU1** (A), **CEPU2** (B), **CEPU3** (C), and **CEPU4** (D), using a normal force of 1 N and a frequency of 1 Hz.

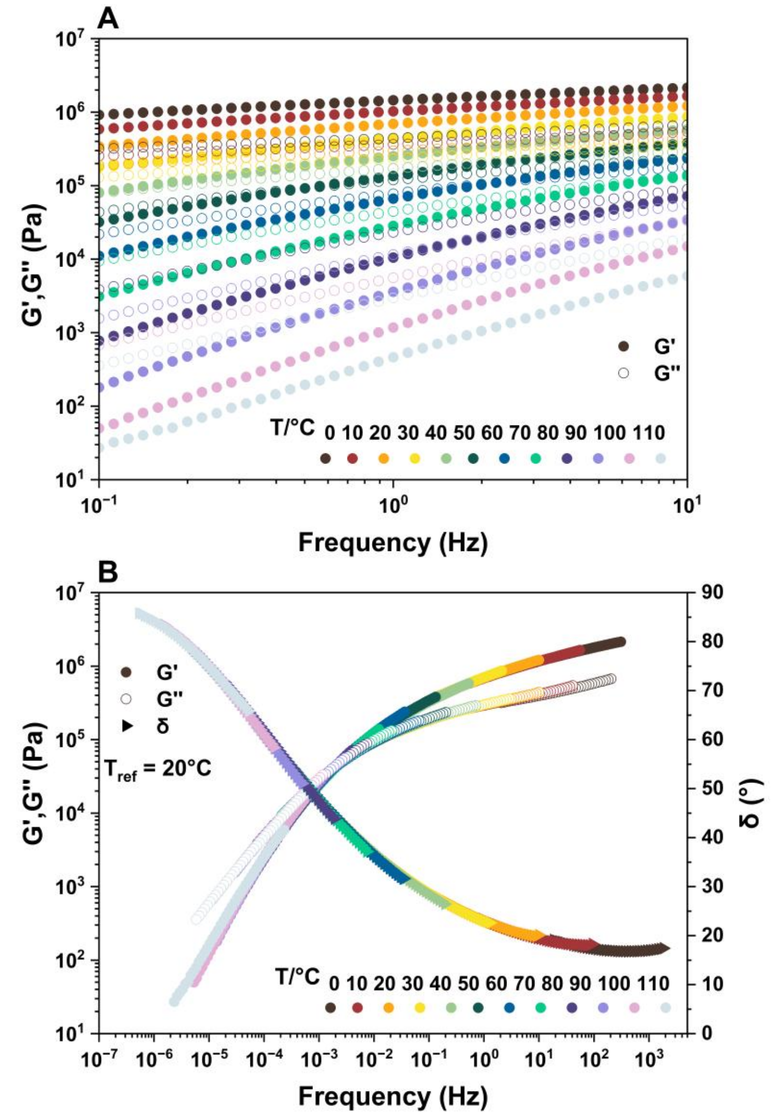


**Figure S46**. (A) Raw rheological data for **CEPU1**, (B) Master curves with a reference temperature ($T_{ref}$) of 20 °C, the rheological master curves were obtained by shifting the frequency sweep curves of different temperatures horizontally (αT) without shifting in the vertical.

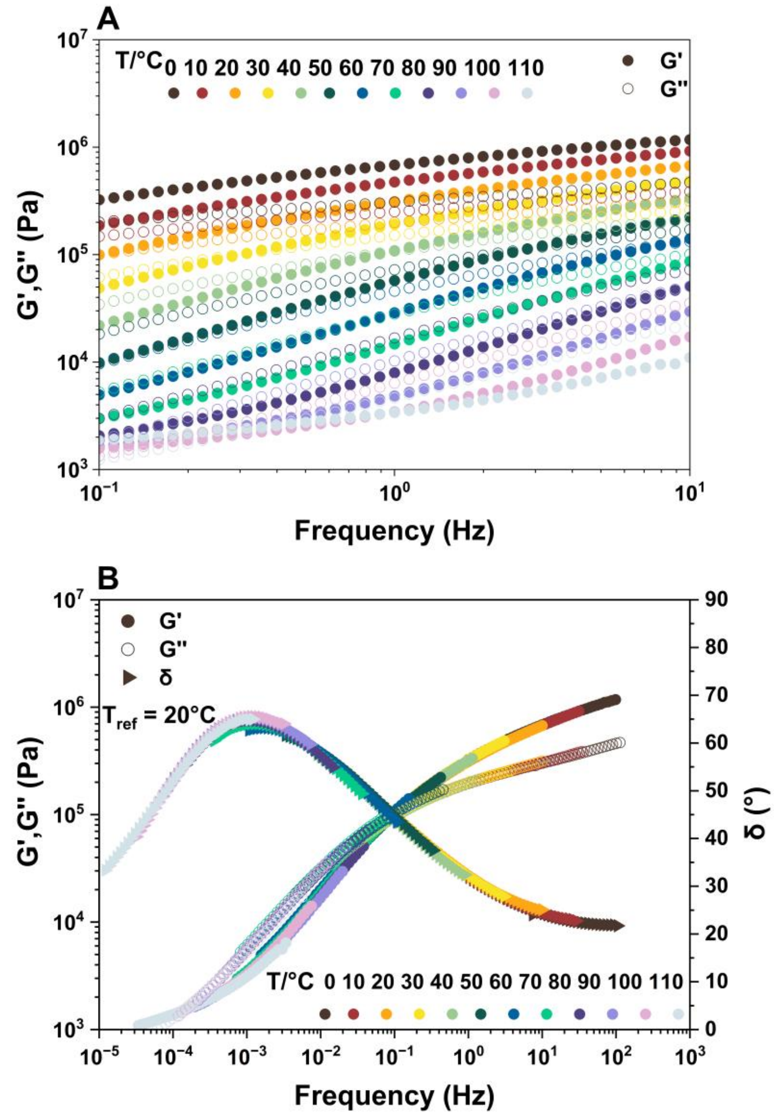


**Figure S47**. (A) Raw rheological data for **CEPU2**, (B) Master curves with a reference temperature ($T_{ref}$) of 20 °C, the rheological master curves were obtained by shifting the frequency sweep curves of different temperatures horizontally (αT) without shifting in the vertical.

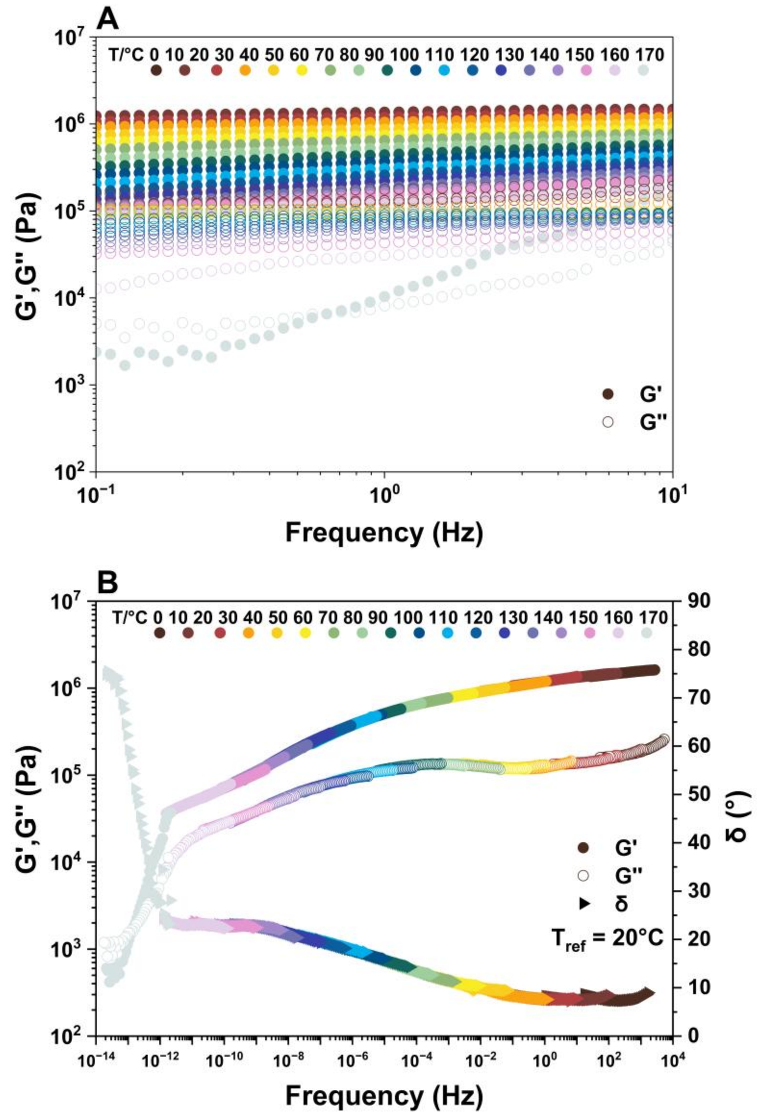


**Figure S48**. (A) Raw rheological data for **CEPU3**, (B) Master curves with a reference temperature ($T_{ref}$) of 20 °C, the rheological master curves were obtained by shifting the frequency sweep curves of different temperatures horizontally (αT) without shifting in the vertical.

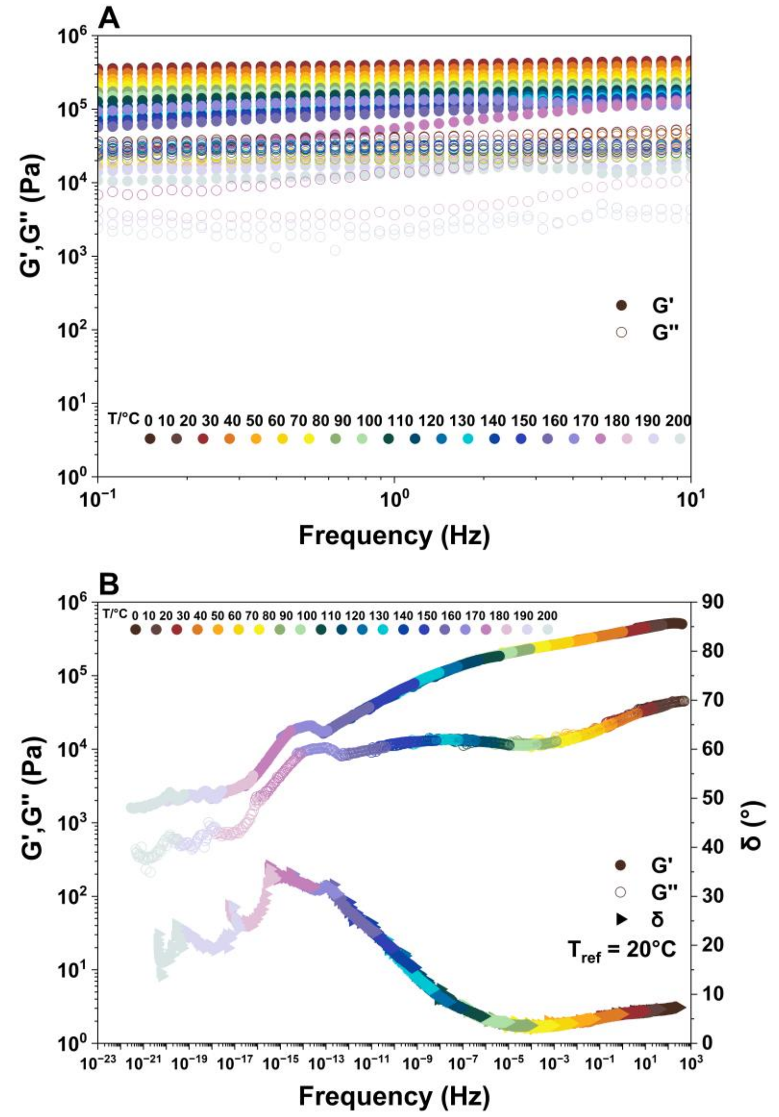


**Figure S49**. (A) Raw rheological data for **CEPU4**, (B) Master curves with a reference temperature ($T_{ref}$) of 20 °C, the rheological master curves were obtained by shifting the frequency sweep curves of different temperatures horizontally (αT) without shifting in the vertical.

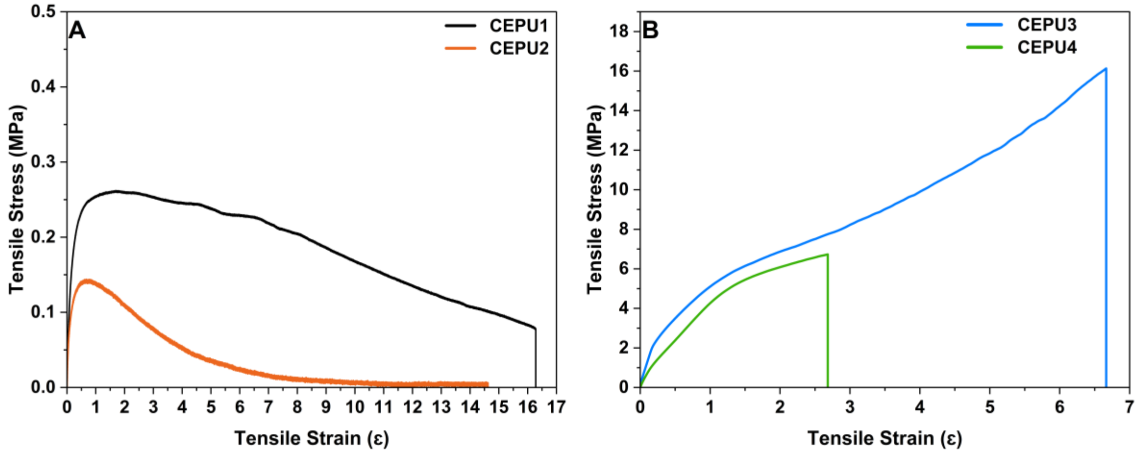


**Figure S50**. Tensile stress-strain curves of CEPUs, (A) **CEPU1** and **CEPU2**, (B) **CEPU3** and **CEPU4**.

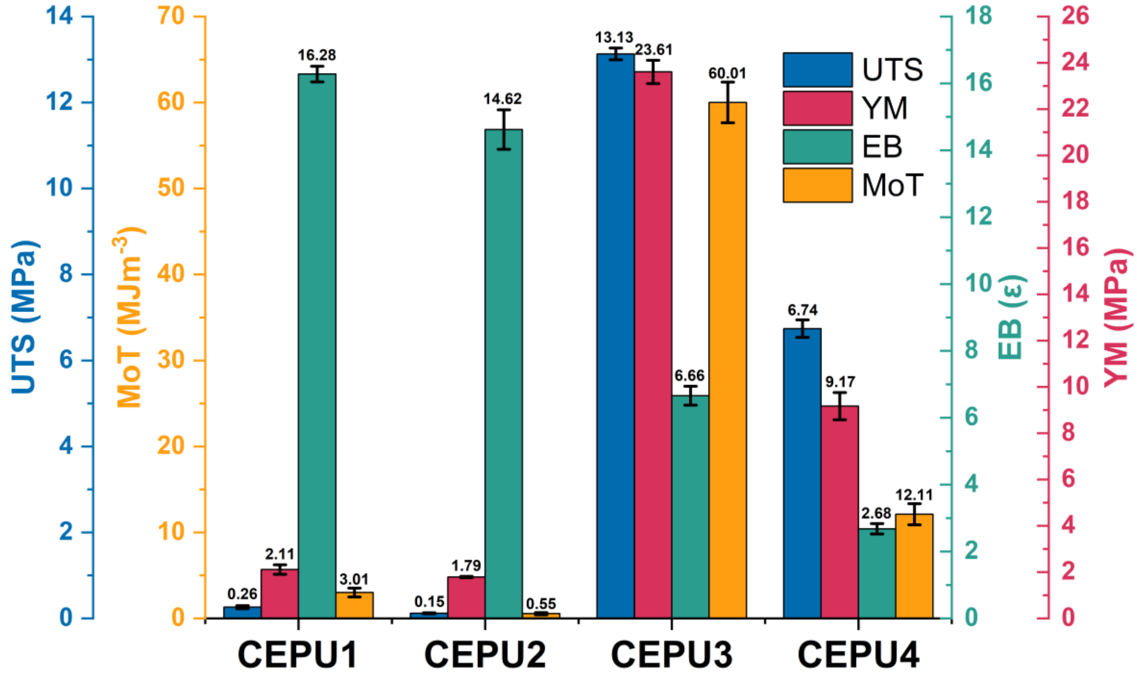


**Figure S51**. Mechanical properties, Ultimate Tensile Strength (UTS), Young's modulus (YM), Modulus of toughness (MoT), and elongation at break (EB) for **CEPU1-CEPU4**. The error shown is the standard deviation (values shown are the averages of 3 repeat measurements for each sample).

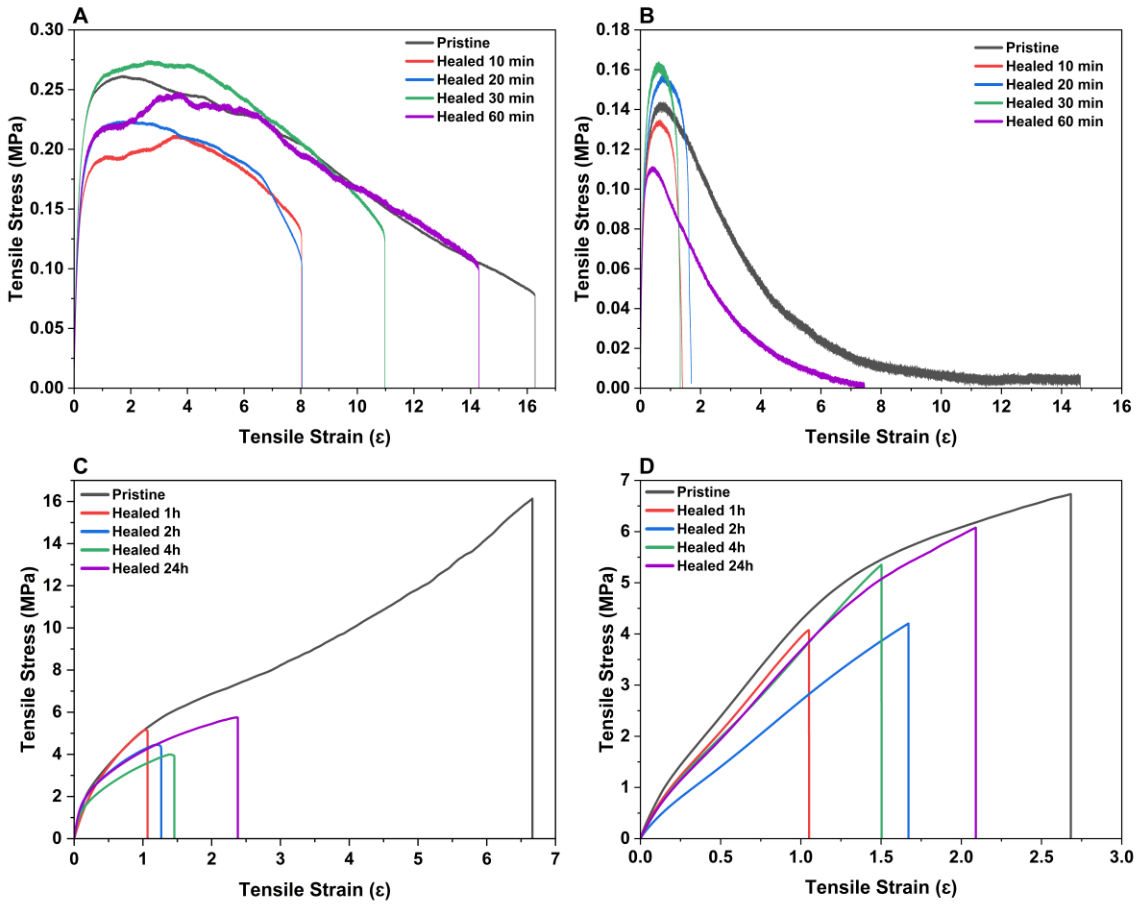


**Figure S52**. Tensile stress-strain curves of the pristine and healed **CEPU1** (A), **CEPU2** (B), **CEPU3** (C), and **CEPU4** (D) at different healing times.

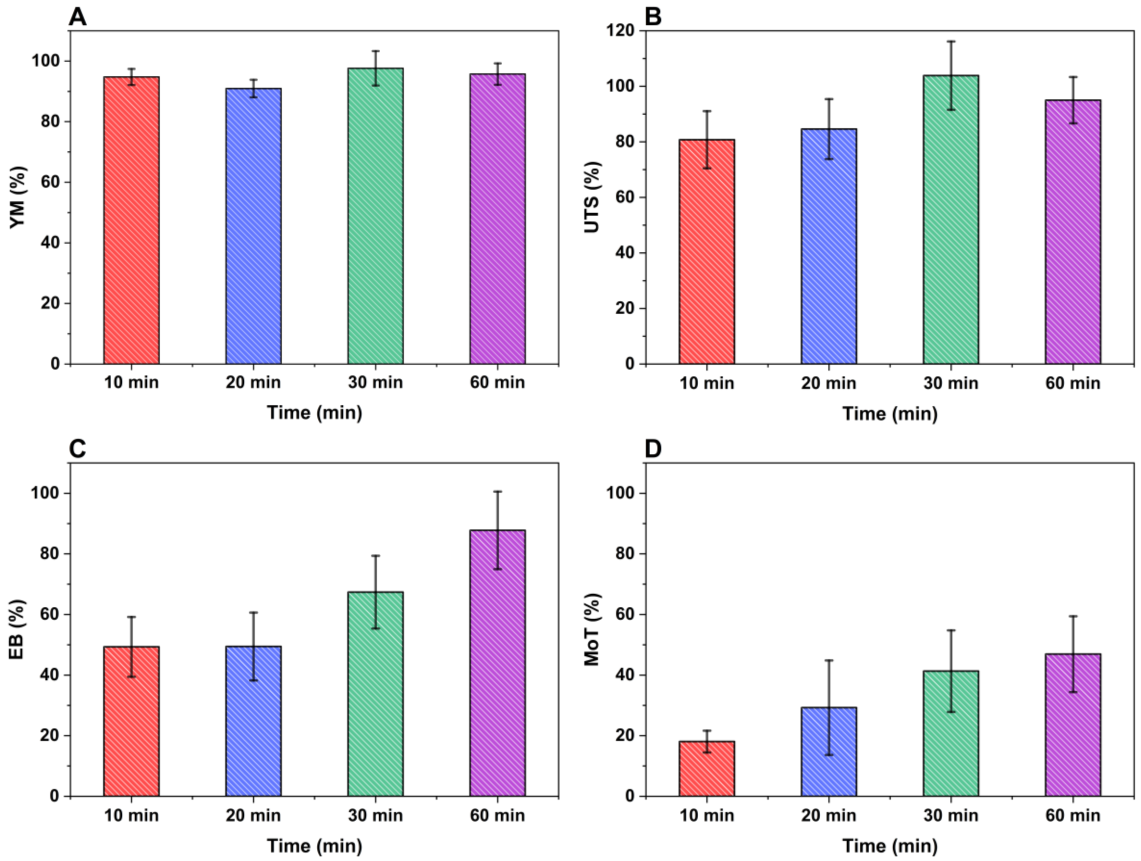


**Figure S53**. Healing efficiency of the YM (A), UTS (B), EB (C), and MoT (D) of the healed **CEPU1** at different healing times at 40 °C. The error shown is the standard deviation (values shown are the averages of 3 repeat measurements for each sample).

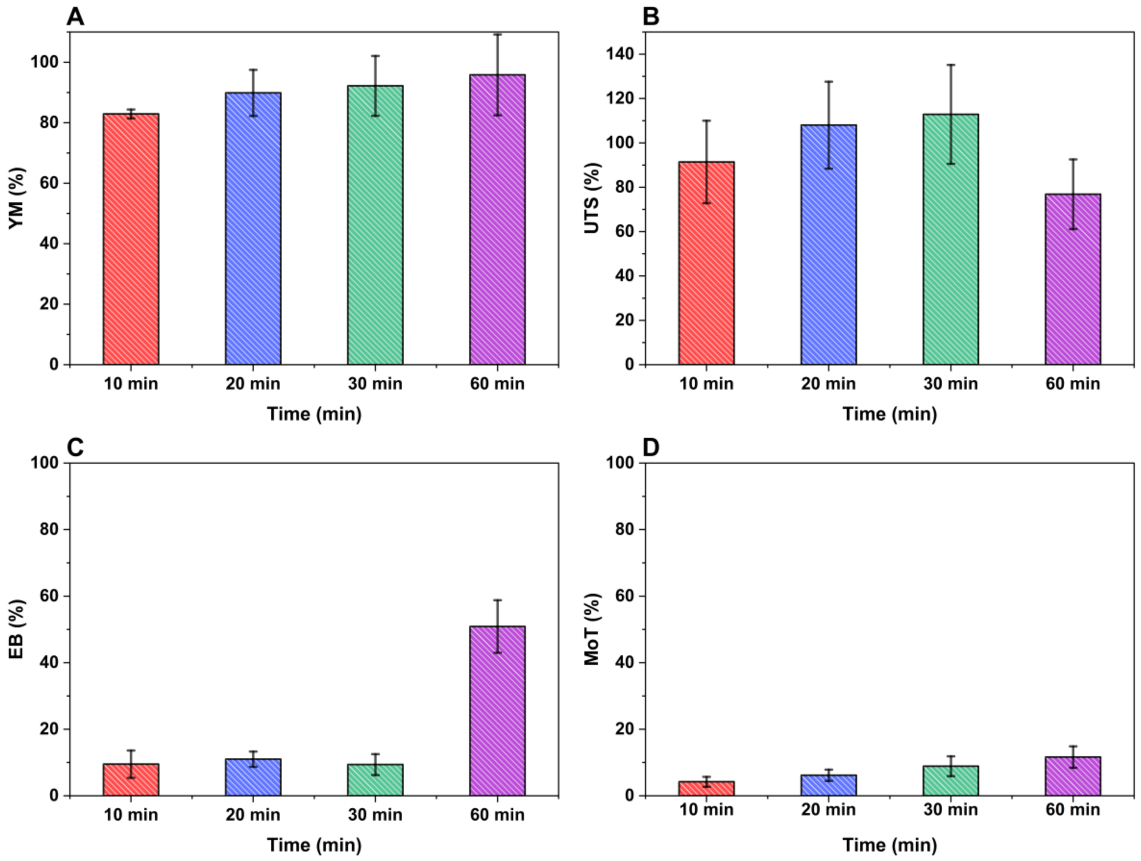


**Figure S54**. Healing efficiency of the YM (A), UTS (B), EB (C), and MoT (D) of the healed **CEPU2** at different healing times at 40 °C. The error shown is the standard deviation (values shown are the averages of 3 repeat measurements for each sample).

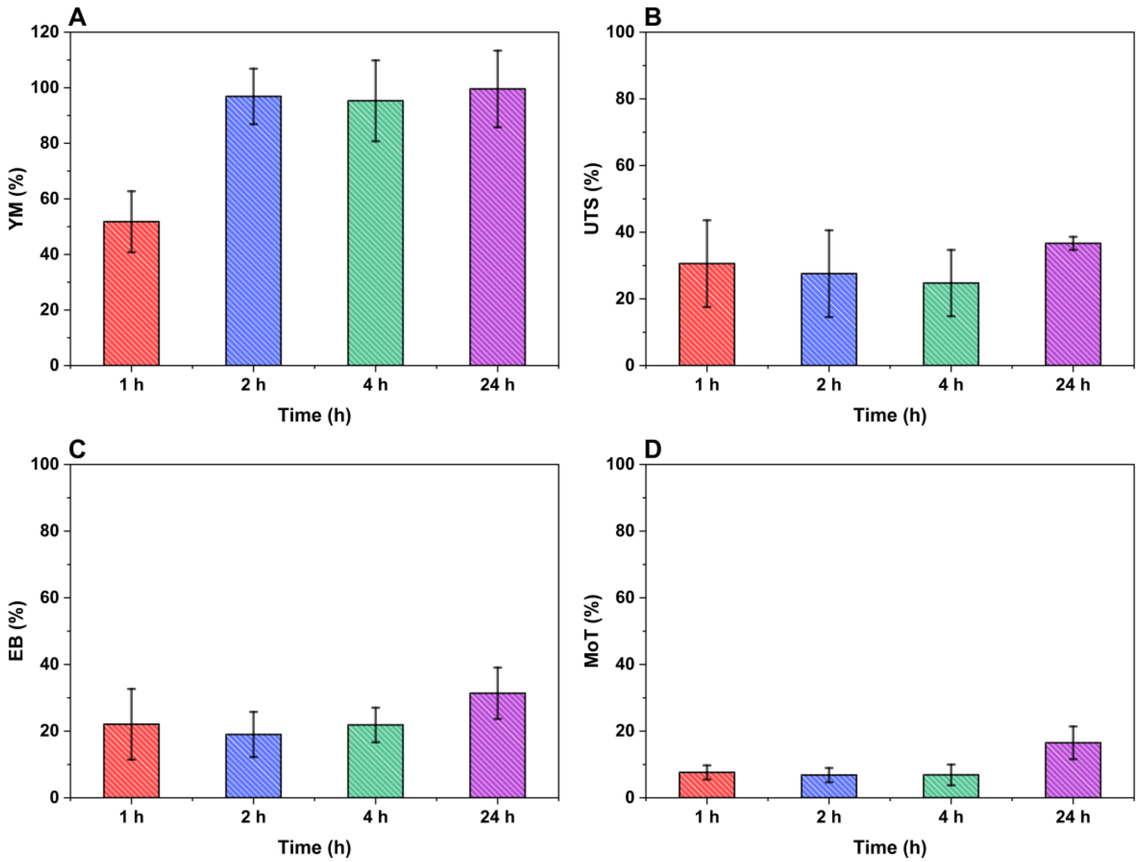


**Figure S55**. Healing efficiency of the YM (A), UTS (B), EB (C), and MoT (D) of the healed **CEPU3** at different healing times at 140 °C. The error shown is the standard deviation (values shown are the averages of 3 repeat measurements for each sample).

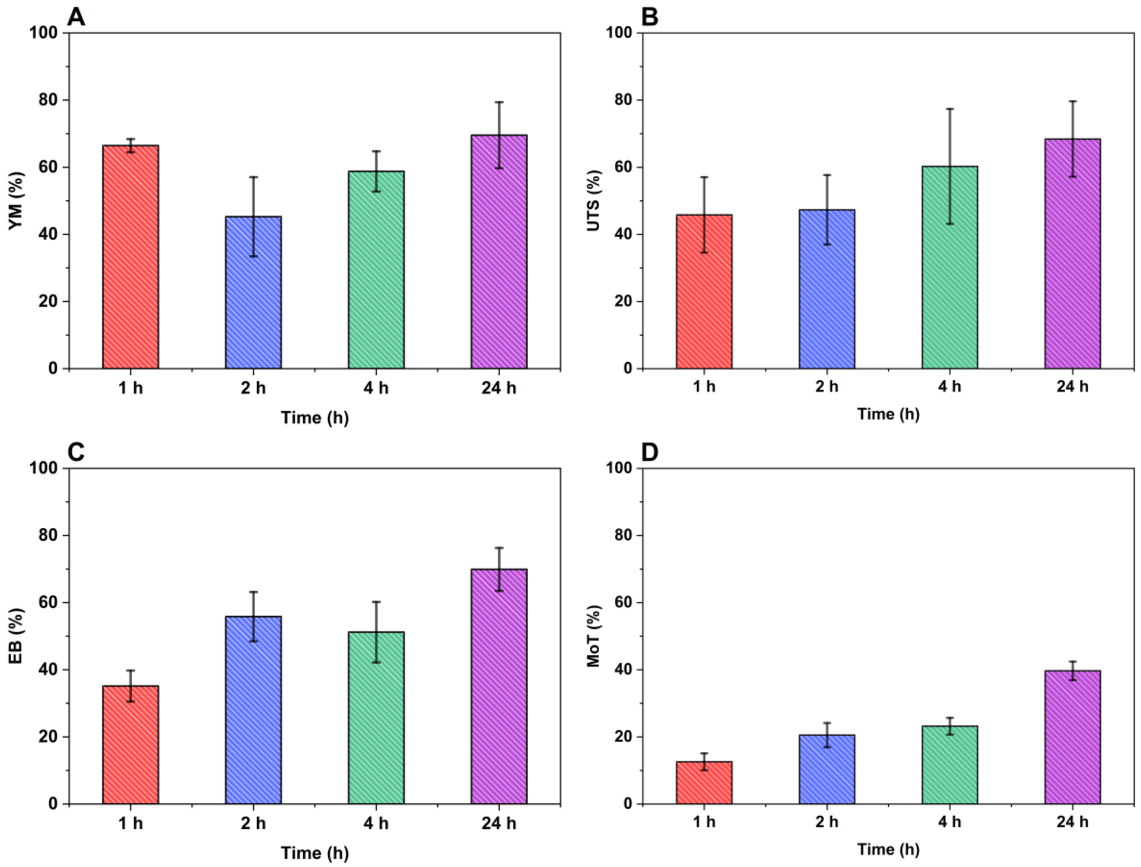


**Figure S56**. Healing efficiency of the YM (A), UTS (B), EB (C), and MoT (D) of the healed **CEPU4** at different healing times at 140 °C. The error shown is the standard deviation (values shown are the averages of 3 repeat measurements for each sample).

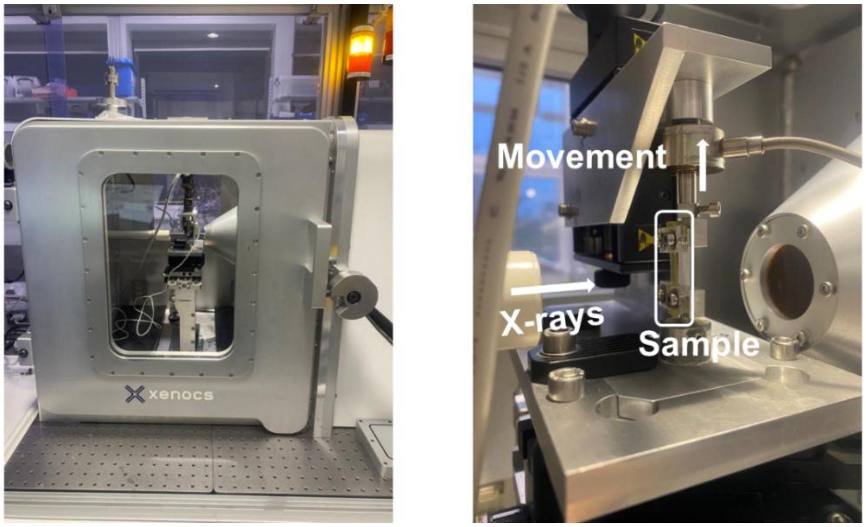


**Figure S57**. In-air setup of labSAXS with mounted micro mechanical tensile tester installed.

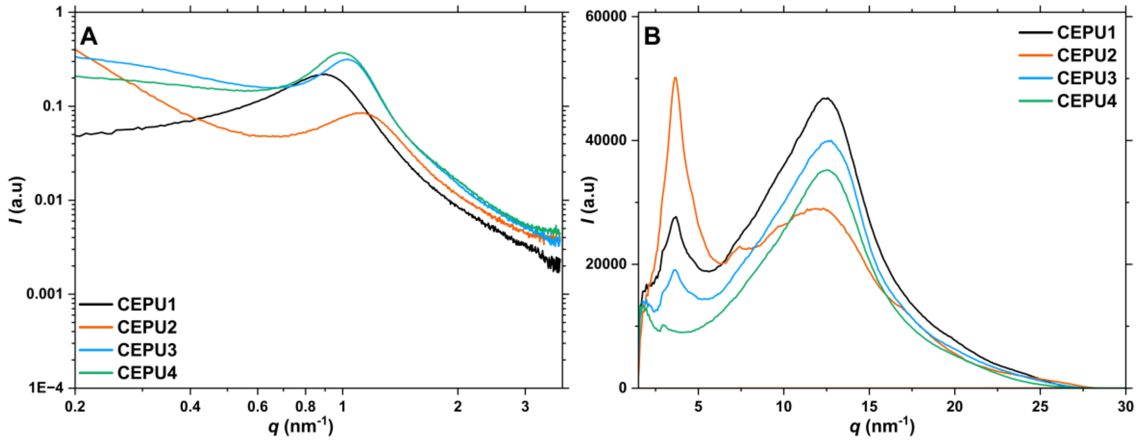


**Figure S58**. (A) SAXS and (B) WAXS scattering patterns of **CEPU1- CEPU4** at 25 ◦C.

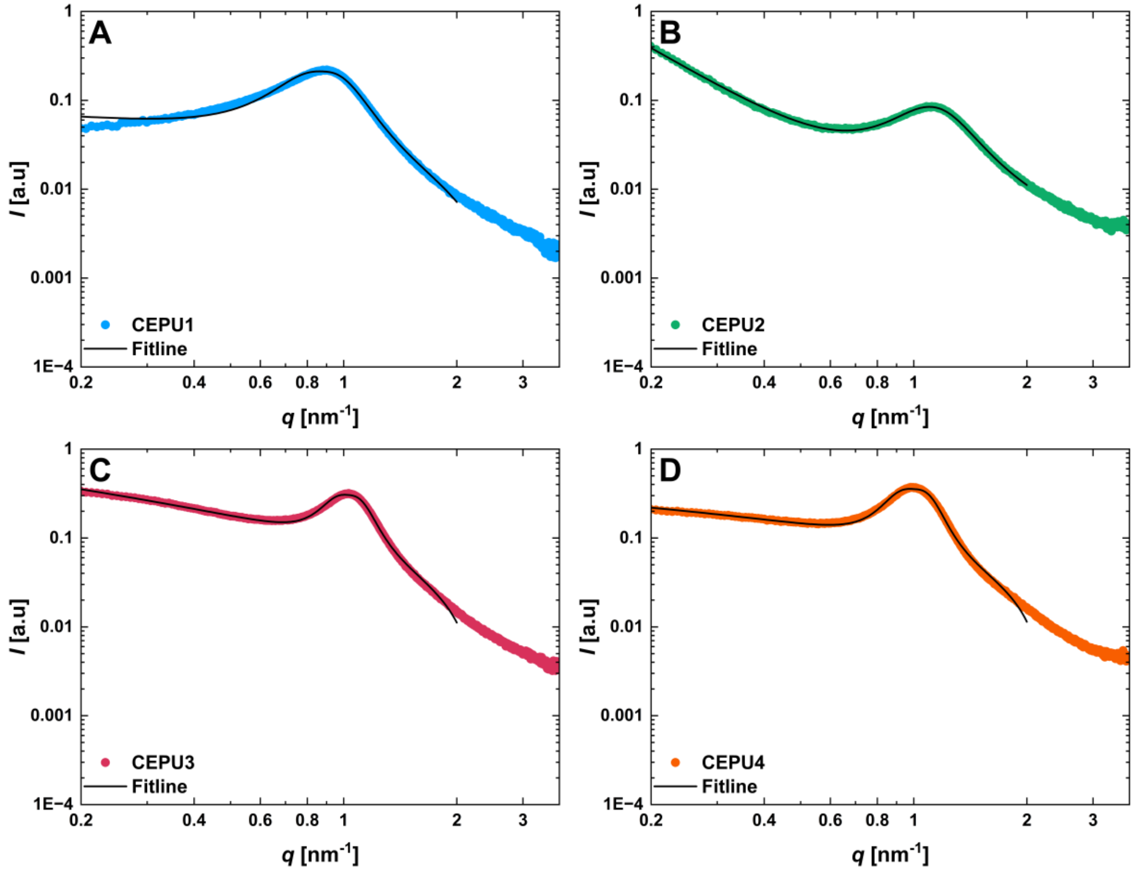


**Figure S59**. 1D SAXS profiles of (A) **CEPU1**, (B) **CEPU2**, (C) **CEPU3**, and (D) **CEPU4** and corresponding fitlines at 25 °C.

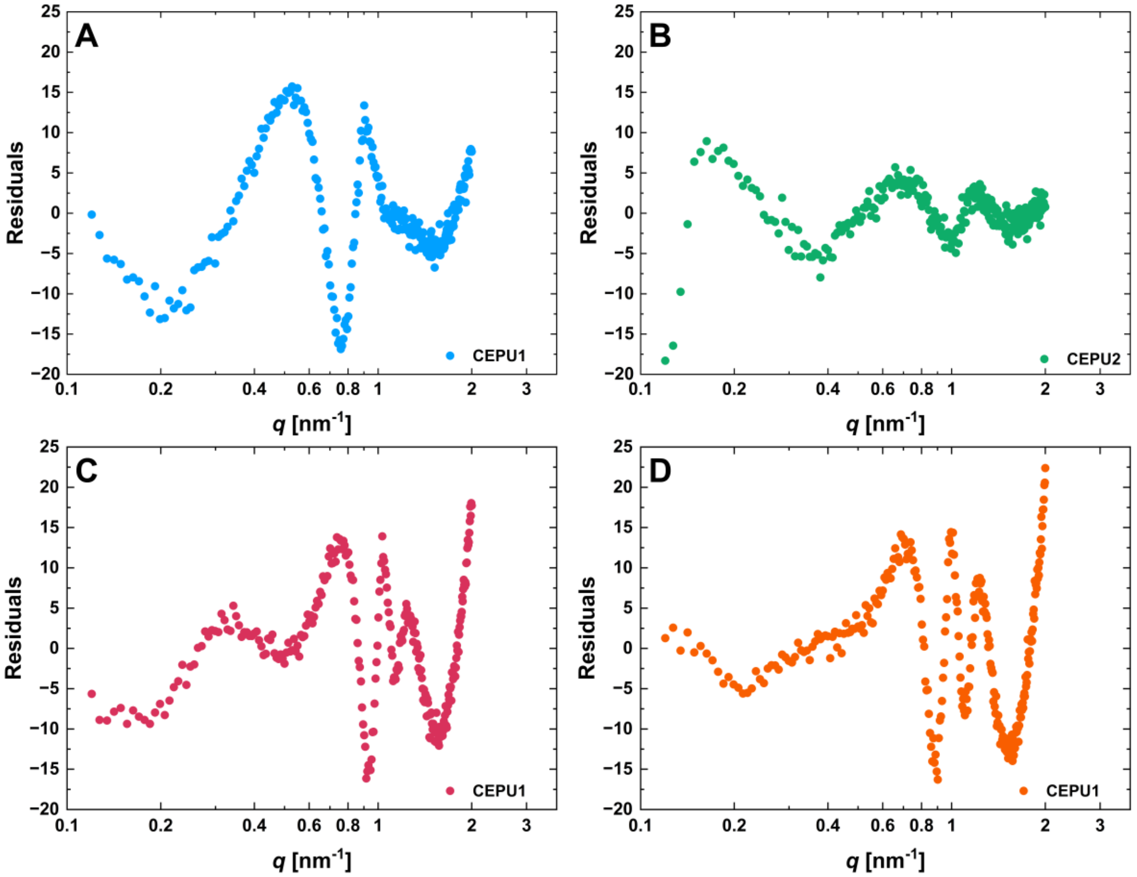


**Figure S60**. SAXS fitting residuals of (A) **CEPU1**, (B) **CEPU2**, (C) **CEPU3**, and (D) **CEPU4** at 25 °C.

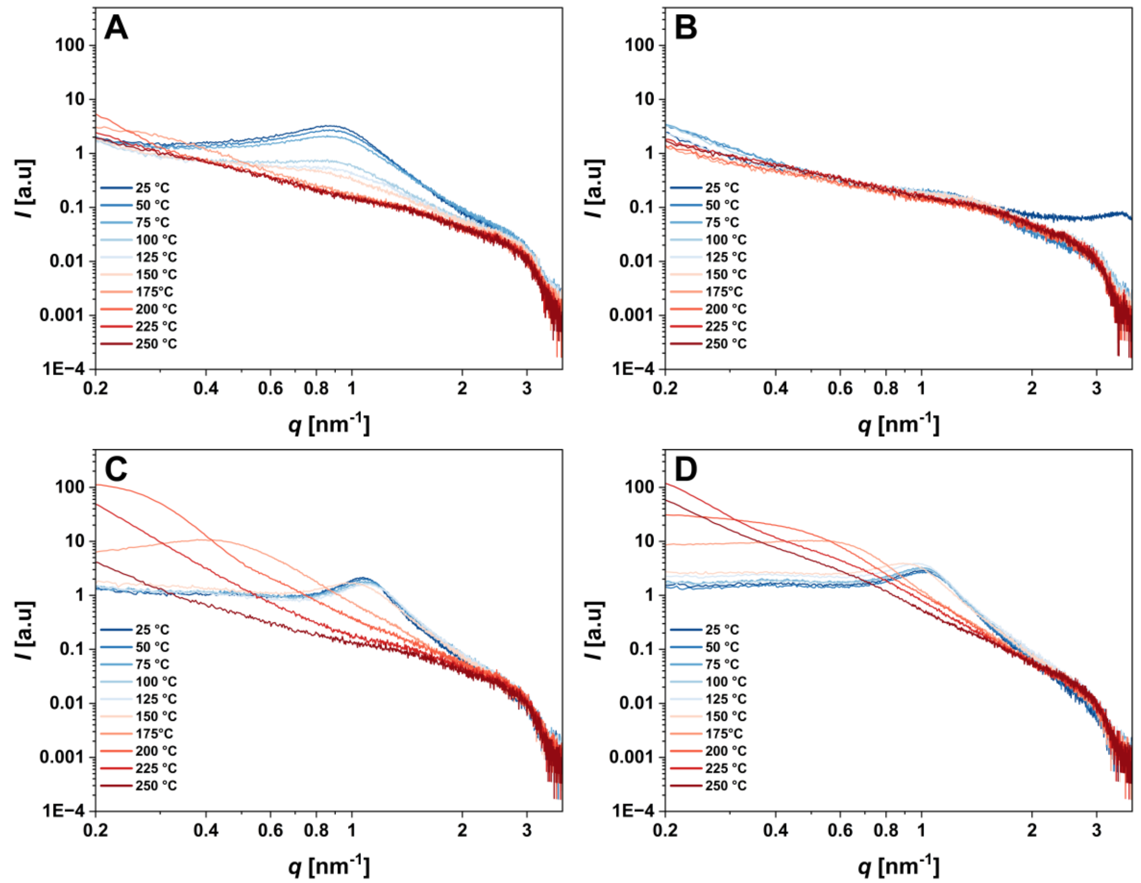


**Figure S61**. VT-SAXS profiles of (A) **CEPU1**, (B) **CEPU2**, (C) **CEPU3** and (D) **CEPU4** as a function of temperature, recorded at 25 °C intervals from 25 °C to 250 °C at a heating rate of 25 °C min$^{-1}$.

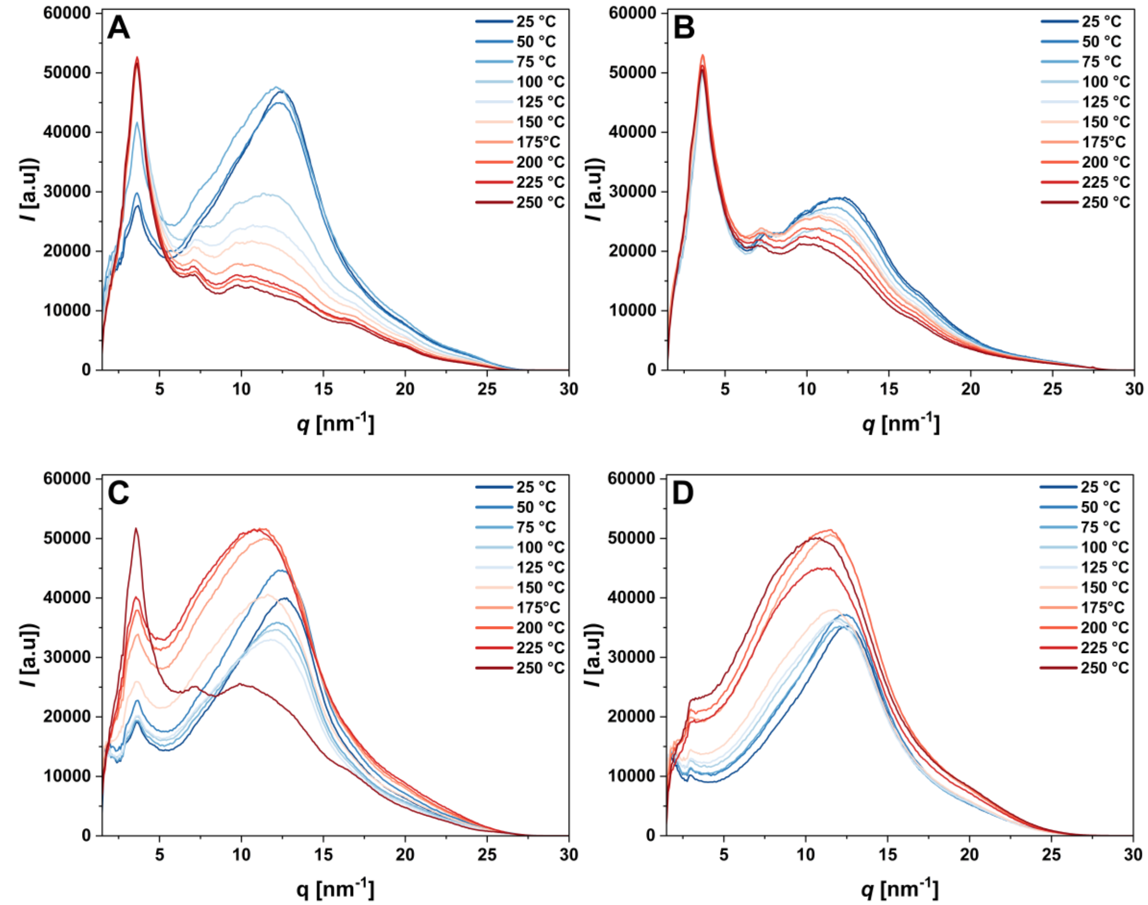


**Figure S62**. VT-WAXS profiles of (A) **CEPU1**, (B) **CEPU2**, (C) **CEPU3** and (D) **CEPU4** as a function of temperature, recorded at 25 °C intervals from 25 °C to 250 °C at a heating rate of 25 °C min$^{-1}$.

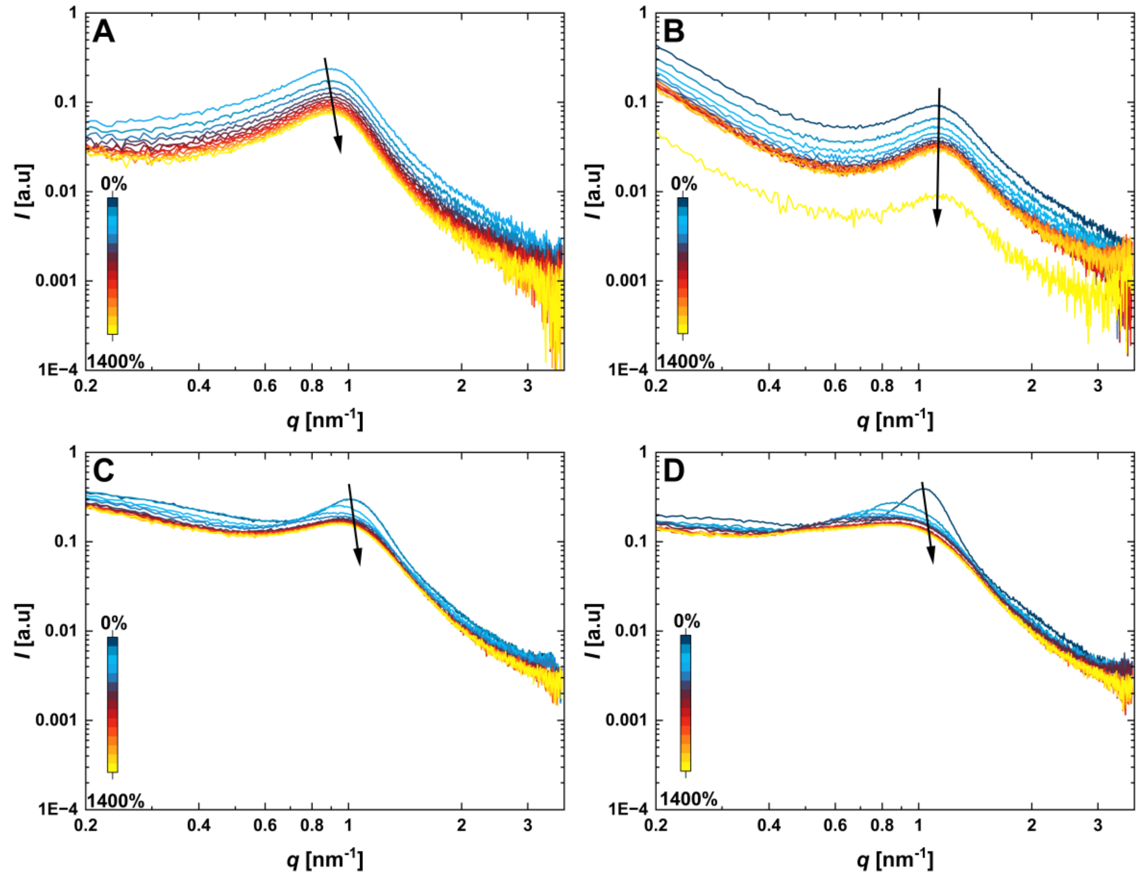


**Figure S63**. The evolution of 1D SAXS pattern as a function of strain from 0% to a maximum of 1400%, (A) **CEPU1**, (B) **CEPU2**, (C) **CEPU3**, and (D) **CEPU4**. The stretching direction is vertical.

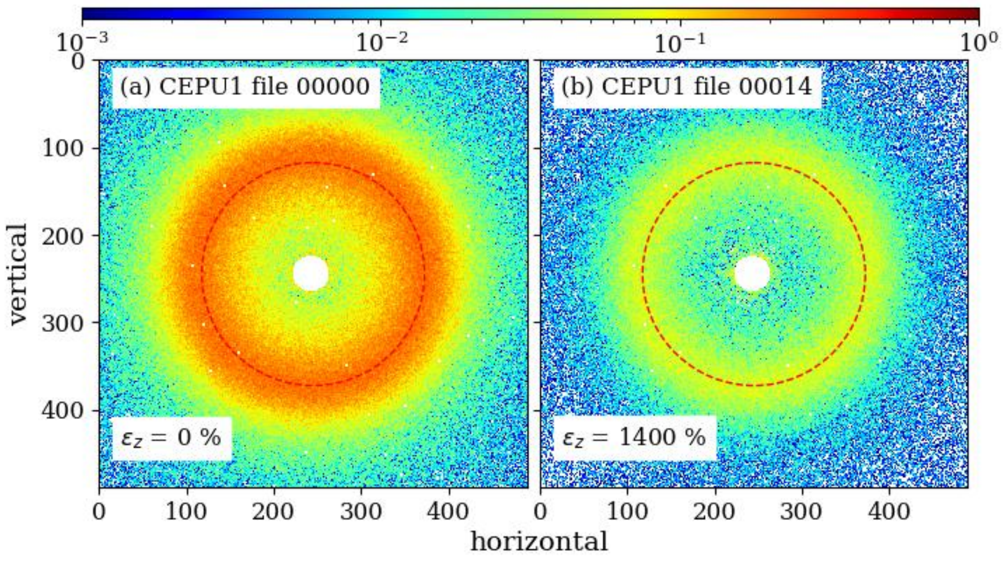


**Figure S64**. 2D SAXS pattern for the **CEPU1** elastomer: (A) at 0% strain and (B) at 1400% strain, respectively.

The red line is a smoothened line of the original data taking a 10-point average.

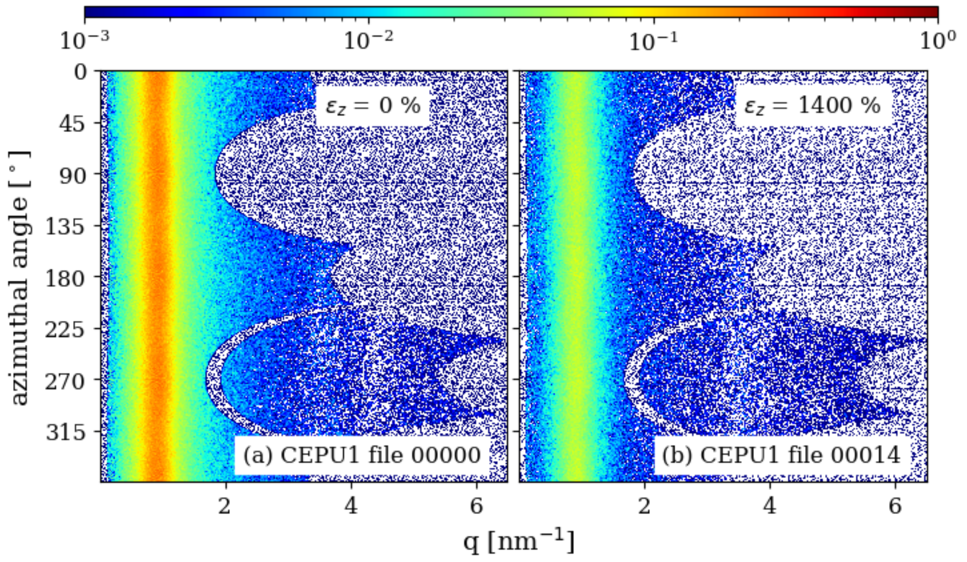


**Figure S65**. Azimuthal profile for the **CEPU1** elastomer: (A) at 0% strain and (B) at 1400% strain, respectively.

The ring appears in a straight band over the full azimuthal angle from 0° to 360° around $q$ = 0.88 nm$^{-1}$.

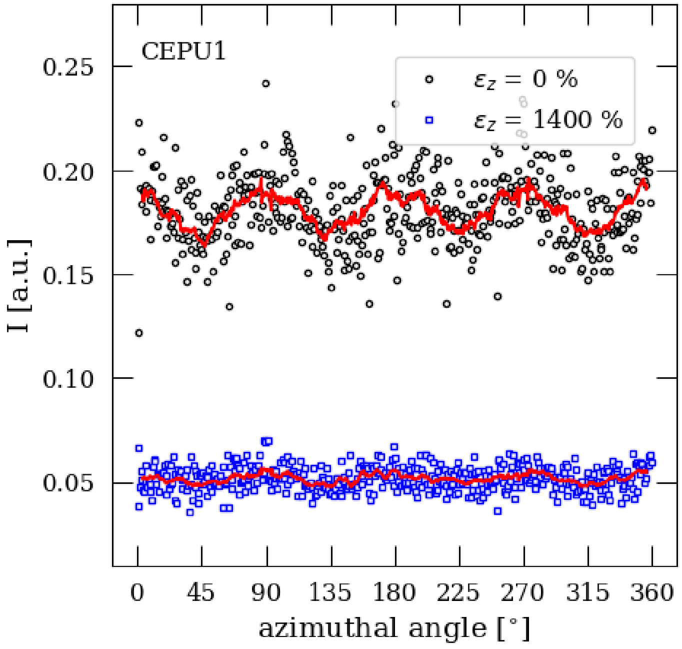


**Figure S66**. Intensity vs azimuthal angle of **CEPU1** elastomer at 0% strain (black open points) and 1400% strain (blue open symbols). The red line is smoothing of the data.

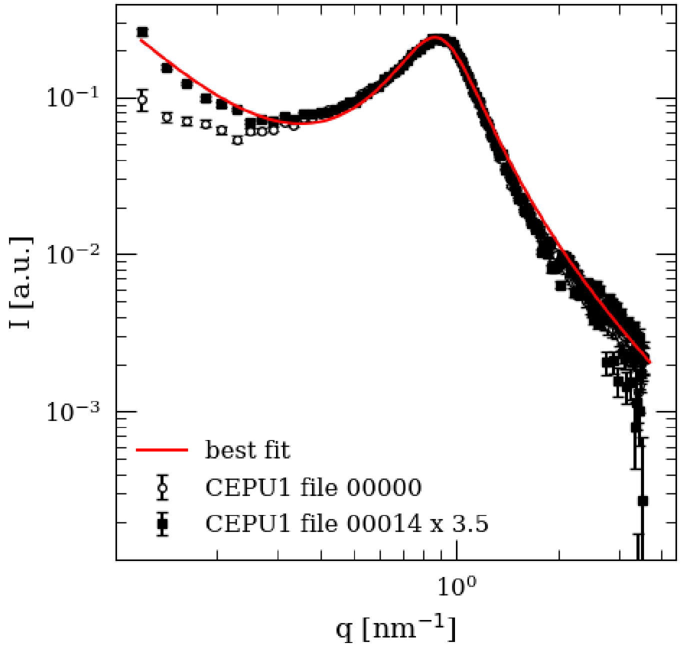


**Figure S67**. Intensity vs q of the **CEPU1** elastomer 0% strain (black open symbols) and 1300% (black squares) strain.

An amorphous correlation ring manifests at a peak maximum around $q_{max}$ = 0.86 nm$^{-1}$ which corresponds to a length scale of 7.23 nm.

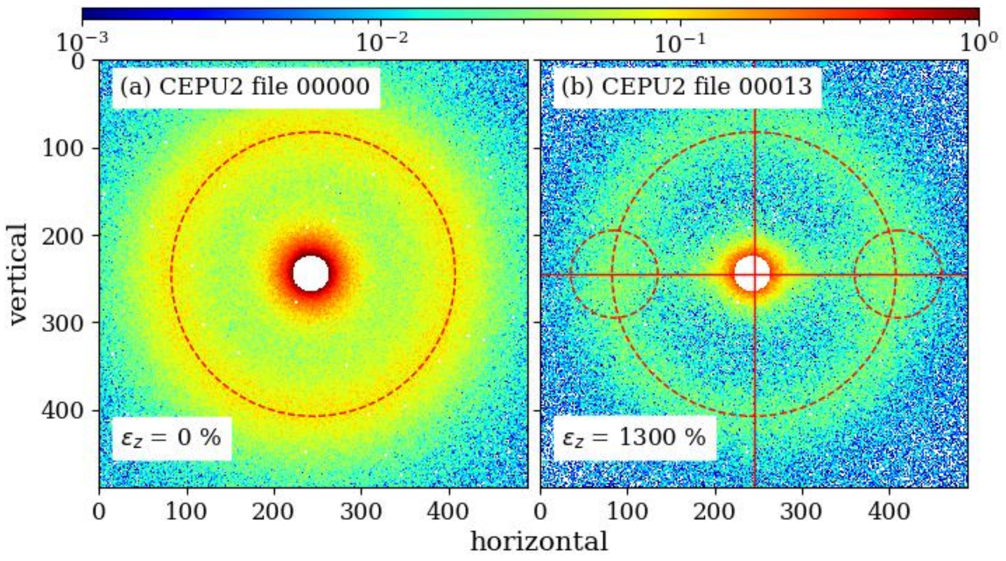


**Figure S68**. 2D SAXS pattern for the **CEPU2** elastomer: (A) at 0% strain and (B) at 1300% strain, respectively.

The scattering is fully isotropic for the covered q-range (indicated by the red dashed circular line). While as before the scattering intensity decreases with increasing strain due to thinning of the sample the 2D scattering pattern develops an anisotropy in the equatorial plane, see (B) indicated by the two red circles.

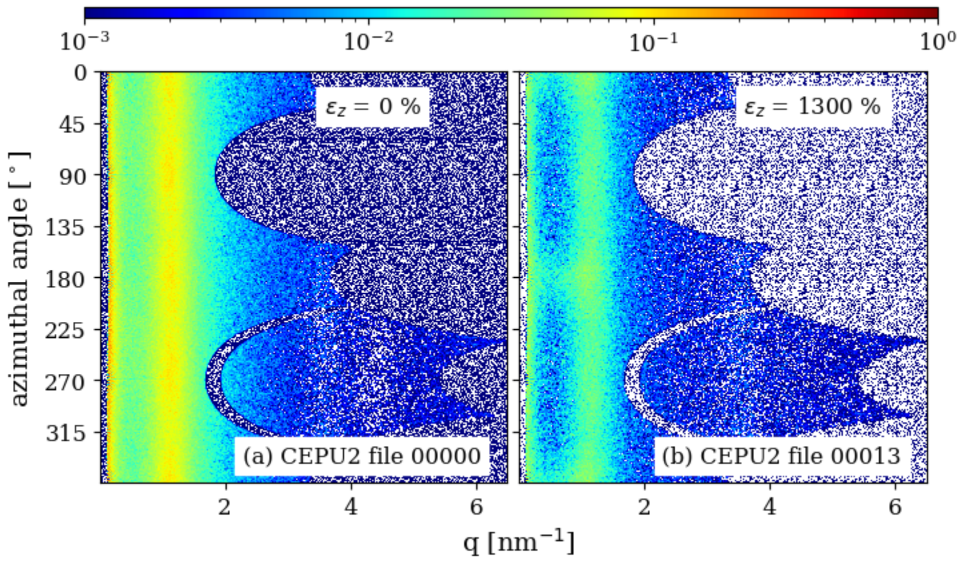


**Figure S69**. Azimuthal profile for the **CEPU2** elastomer: (A) at 0% strain and (B) at 1300% strain, respectively.

**CEPU2** has some sort of structure and preferred orientation even at 0% strain, see (**A**). Additionally, there is an increased intensity on the amorphous correlation ring at 0°, 90° and 180° while stretching. The amorphous correlation ring remains but the intensity increases only at 0° and 180°, see (B).

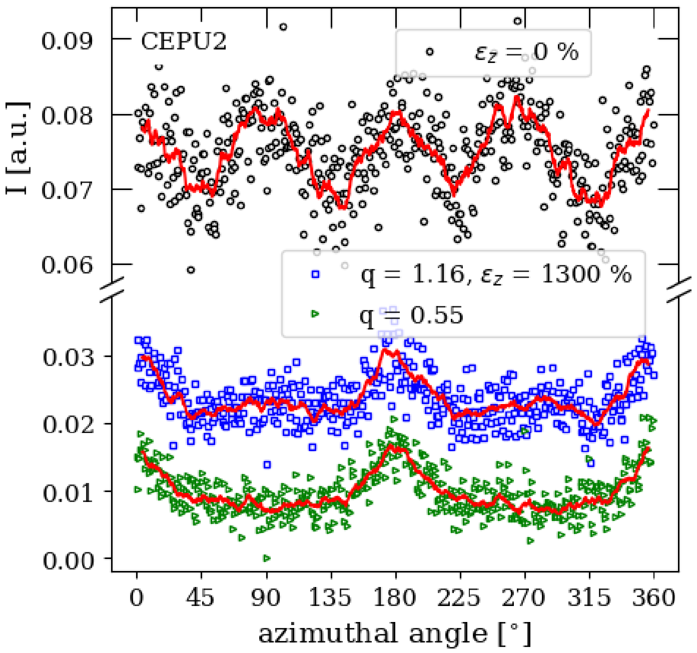


**Figure S70**. Intensity vs azimuthal angle of **CEPU2** elastomer at 0% strain (black open points) and 1300% strain (blue open symbols).
The red line is smoothing of the data. The 1D azimuthal plot averaging over two different q-values ($q_1$ = 0.55 $nm^{-1}$ and $q_2$ = 1.16$nm^{-1}$).

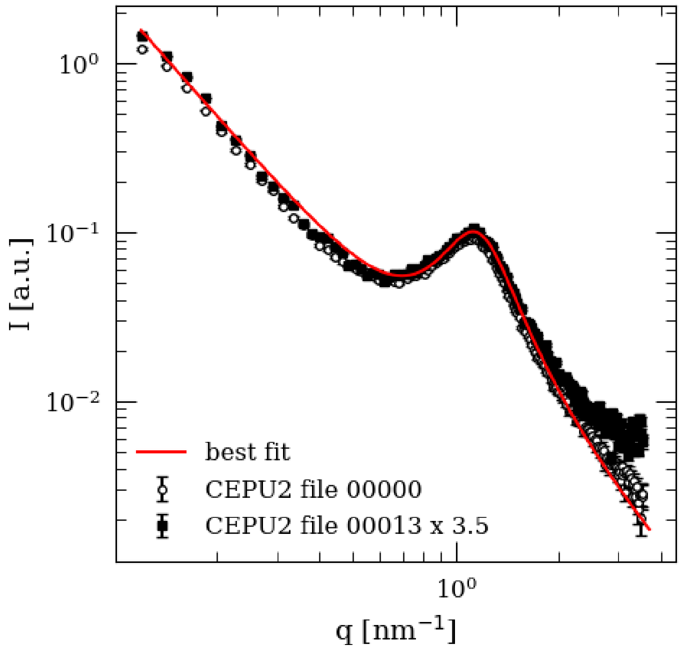


**Figure S71**. Intensity vs q of the **CEPU2** elastomer 0% strain (black open symbols) and 1300% (black squares) strain.
The stretched **CEPU2** had to be shifted by a scaling factor of 3.5 similar to **CEPU1**, accounting for the change of sample thickness when stretched.

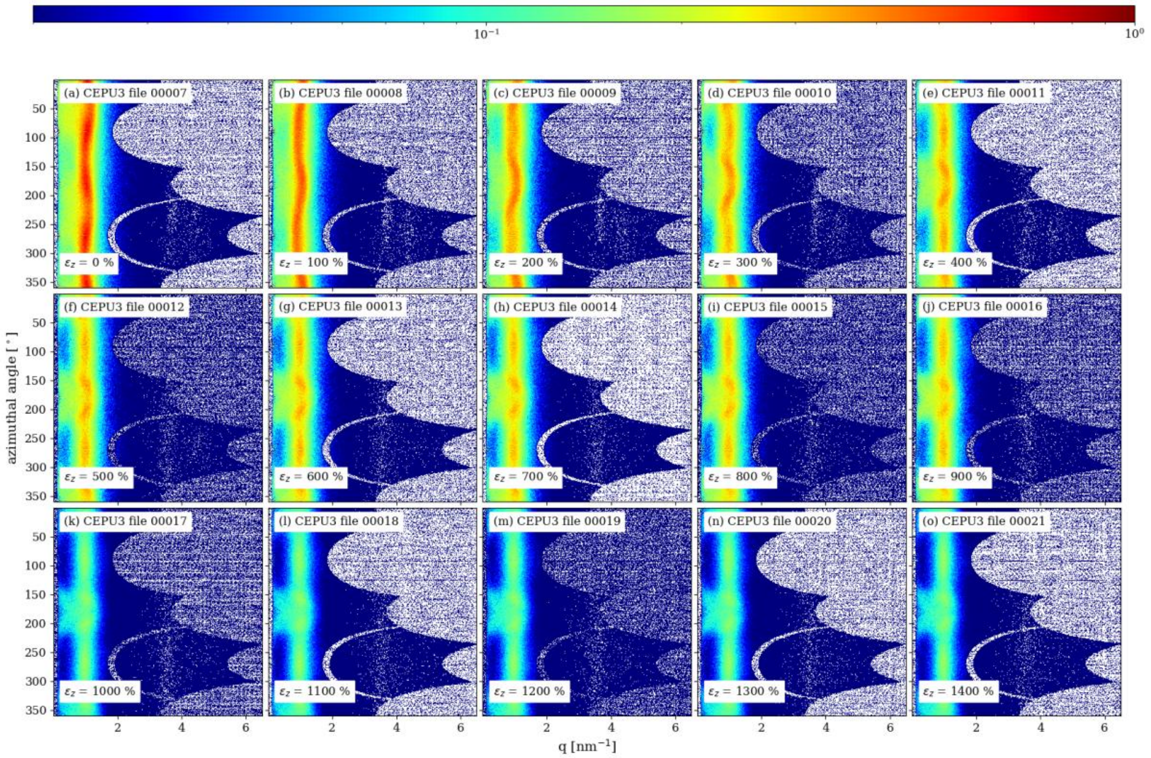


**Figure S72**. Azimuthal angle profiles of the **CEPU3** elastomer for all q-values for increasing stretching 0% to 1400%.

The elliptic scattering was evident in the azimuthal angle profile as a deviation from straight vertical band as shown in image (a).

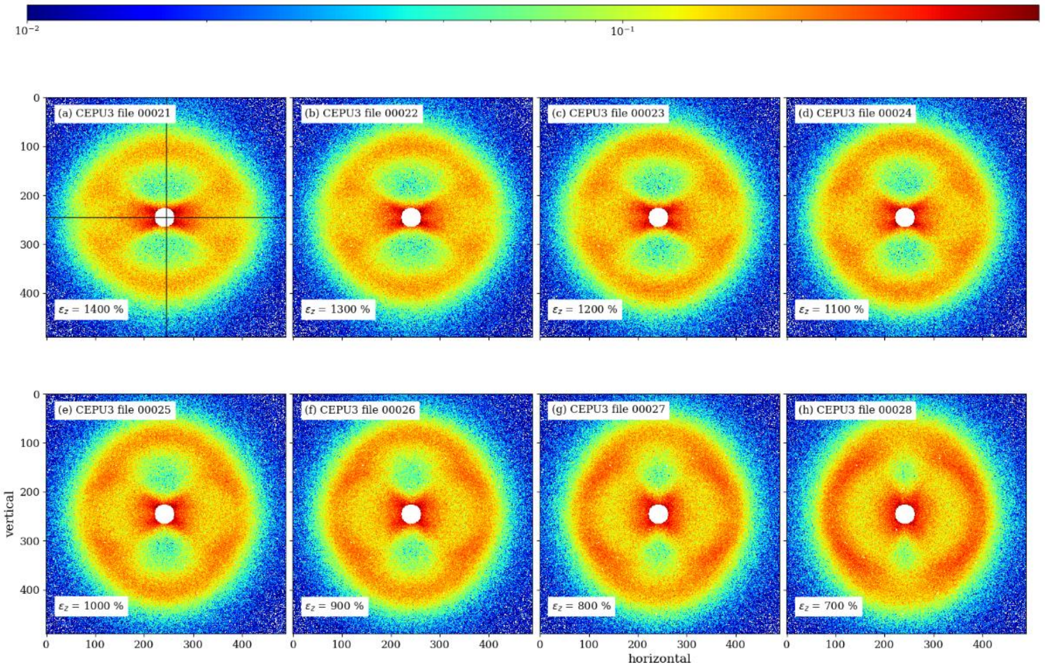


**Figure S73**. The evolution of the 2D-SAXS pattern of **CEPU3** during relaxation from 1400% strain to 700%.

After retracting the strain applied, the sample slowly relaxes such that the four-point pattern broadens again towards lower strains, see the comparison image (a) with image (h).

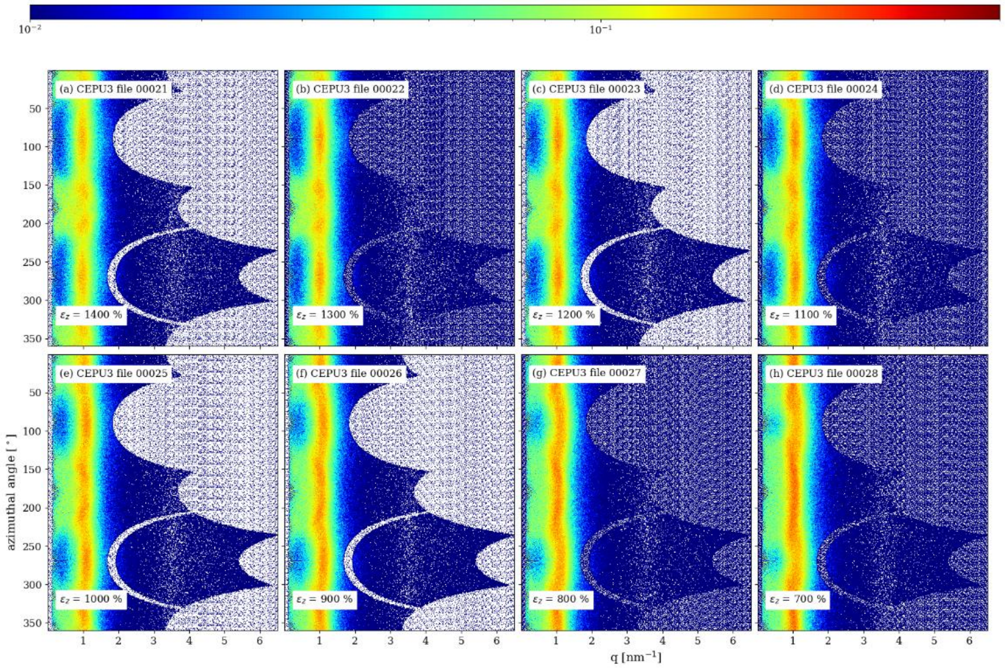


**Figure S74**. Azimuthal angle profiles of **CEPU3** for the relaxation from 1400% strain to 700%.

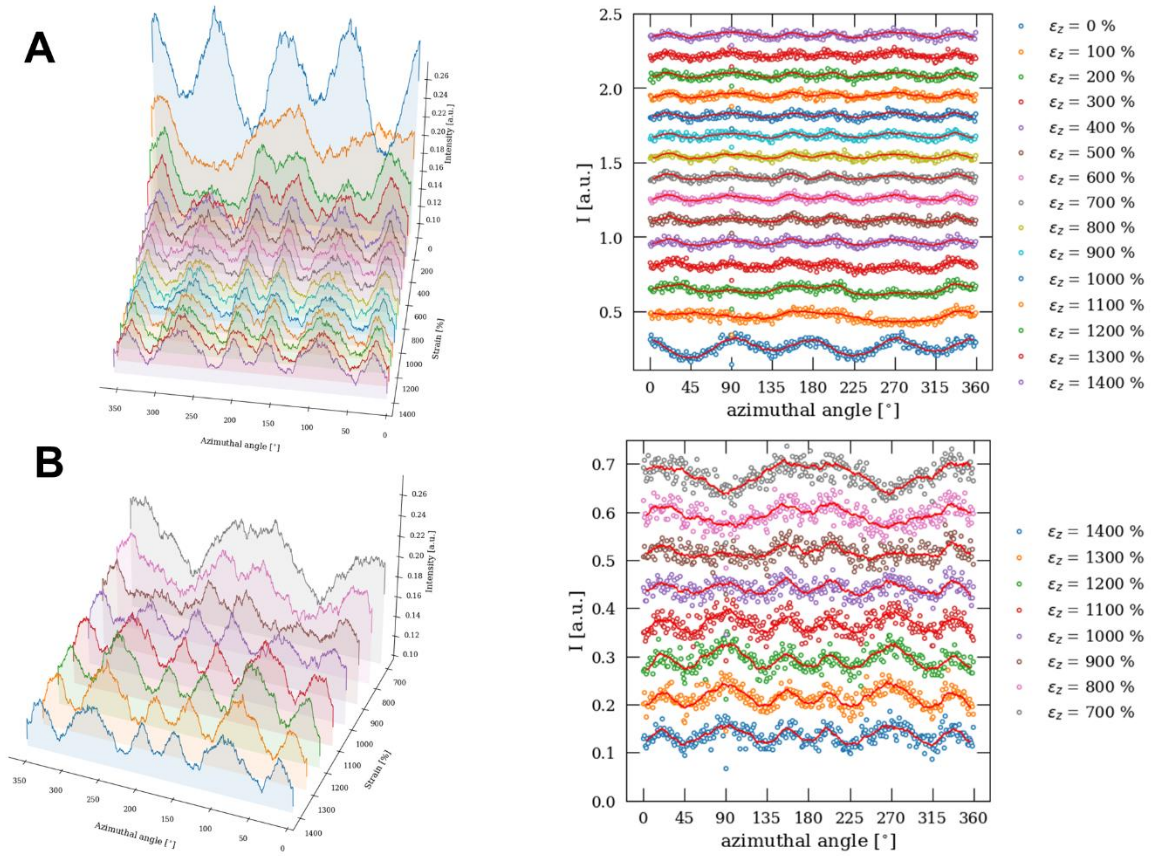


**Figure S75**. Azimuthal angle profile evolution of **CEPU3** at q = 1.02nm$^{-1}$ for each profile the mean value of $q$ was 1.02 nm$^{-1}$ for a range of $\Delta q$ = 0.13 nm$^{-1}$ (A) stretching and (B) relaxation. In the 1D plot the azimuthal profiles are systematically shifted for sake of visibility by an arbitrary constant intensity.

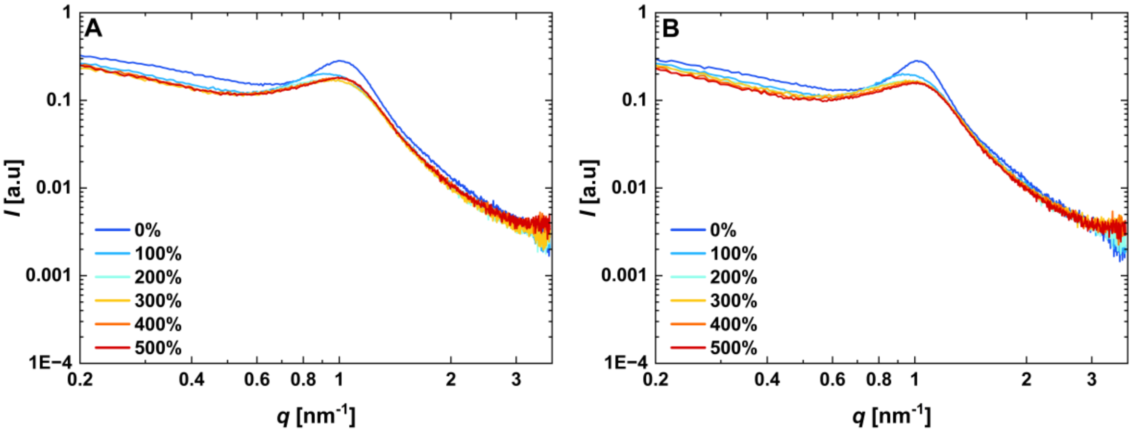


**Figure S76**. Selected 1D-SAXS pattern of **CEPU3** as a function of strain from 0% to 500% taken in the center of the flap (A) and the edge (B).

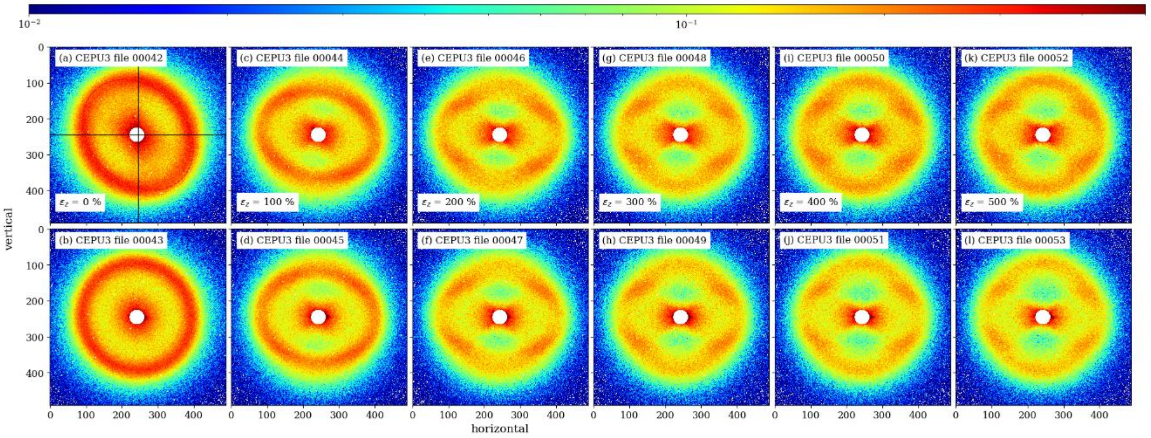


**Figure S77**. Selected 2D-SAXS pattern of **CEPU3** as a function of strain from 0% to 500% taken in the center of the flap (a-k) and the edge (b-l).

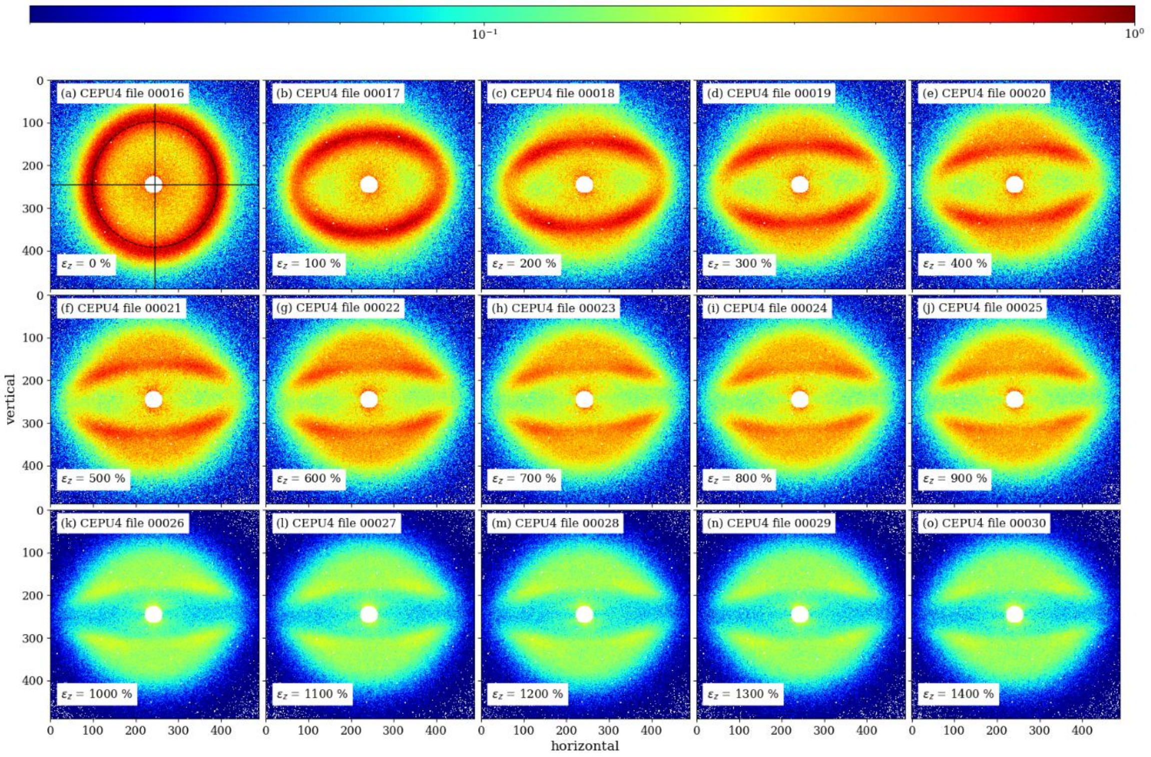


**Figure S78**. The evolution of 2D-SAXS pattern of **CEPU4** as a function of strain from 0% to a maximum of 1400%.

The stretching direction is vertical. The azimuthal peak intensity displayed a structural anisotropy. In the meridian direction, i.e. along the deformation axis two arcs with increased scattering intensity appear, with further deformation the arcs split into a four-point pattern, see image (c), which can be attributed to tilted hard segments along the stretching direction. All SAXS profiles during the deformation have in common that the equator or meridian, respectively, is slightly tilted by *ca.* 2 ° because of the variation of the fine texture in the necking portions, i.e. measuring away from the central axis.

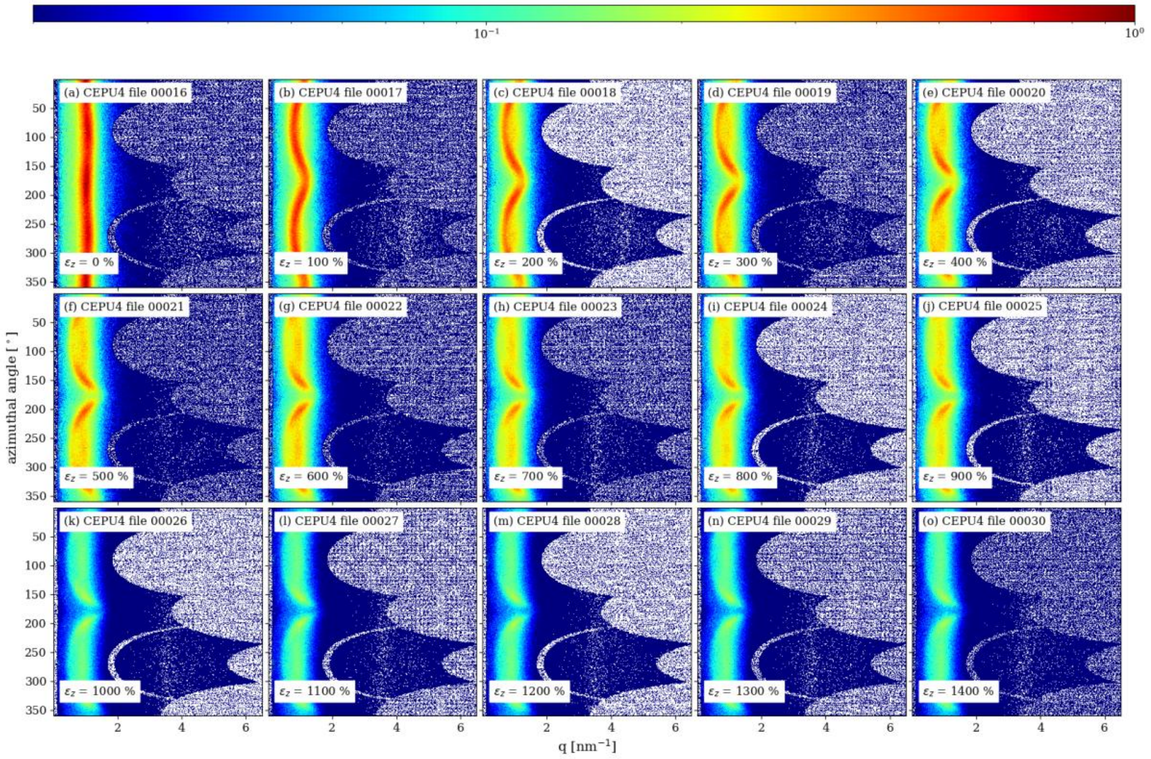


**Figure S79**. Azimuthal angle profiles of the **CEPU4** elastomer for all q-values for increasing stretching 0% to 1400%.

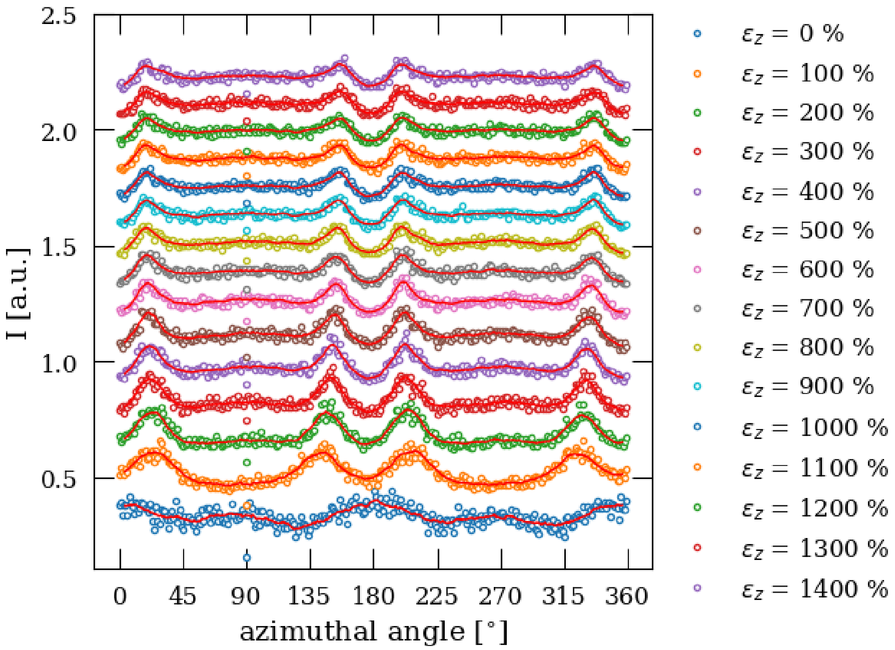

**Figure S80**. Azimuthal angle profiles of the **CEPU4** elastomer for all q-values for increasing stretching 0% to 1400%.

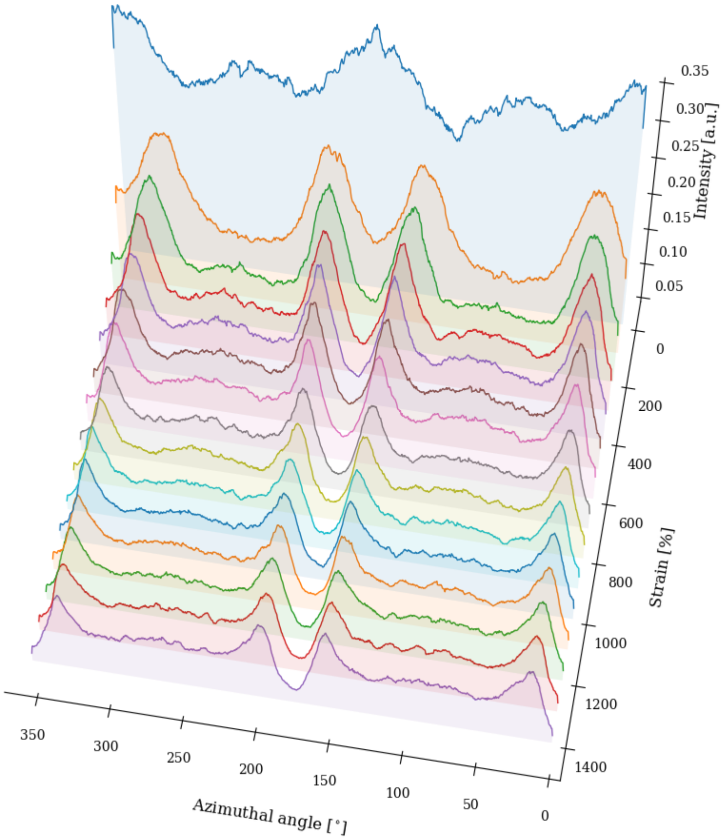


**Figure S81**. Azimuthal angle profiles of the **CEPU4** elastomer for all q-values for increasing stretching 0% to 1400%.

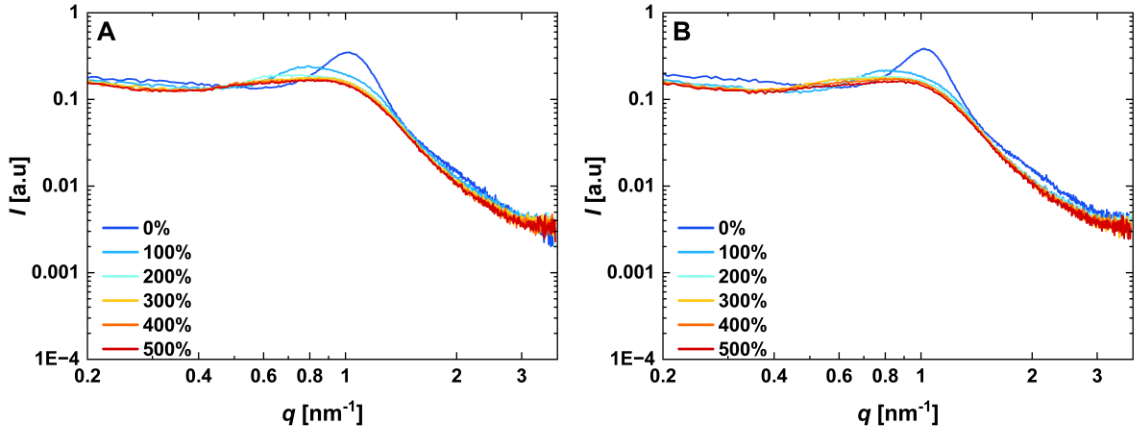


**Figure S82**. Selected 1D-SAXS pattern of **CEPU4** as a function of strain from 0% to 500% taken in the center of the flap (A) and the edge (B).

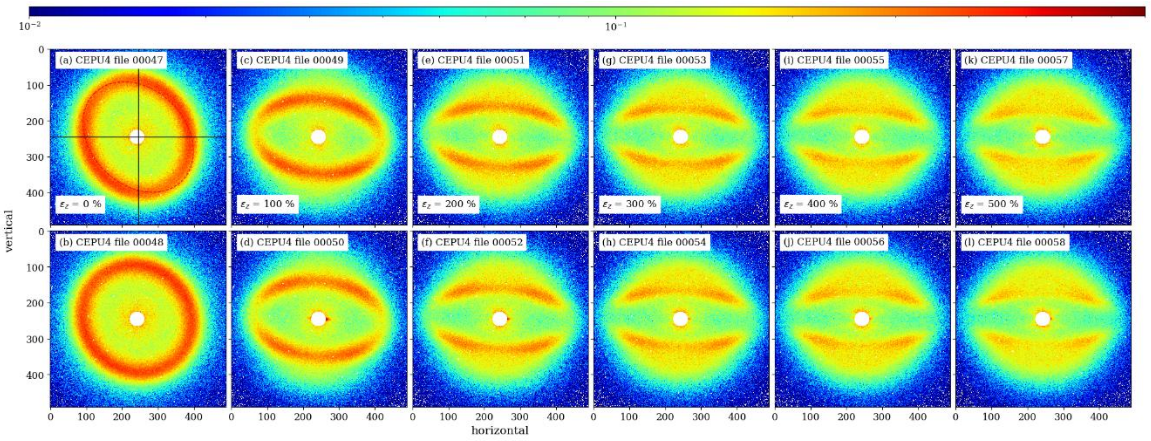


**Figure S83**. Selected 2D-SAXS pattern of **CEPU4** as a function of strain from 0% to 500% taken in the center of the flap (a-k) and the edge (b-l).
The tilt varies from specimen to specimen and depending on how far away from the central axis the measurement was taken where the tilt is changed.

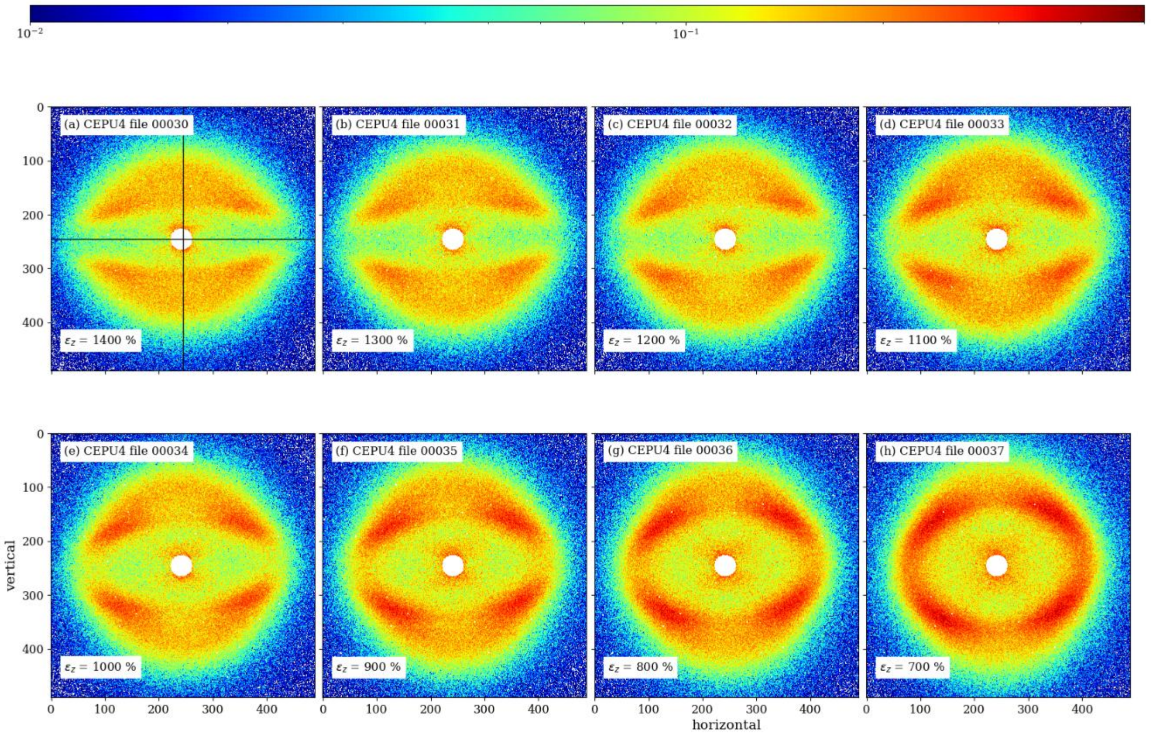


**Figure S84**. The evolution of the 2D-SAXS pattern of **CEPU4** during relaxation from 1400% strain to 700%.

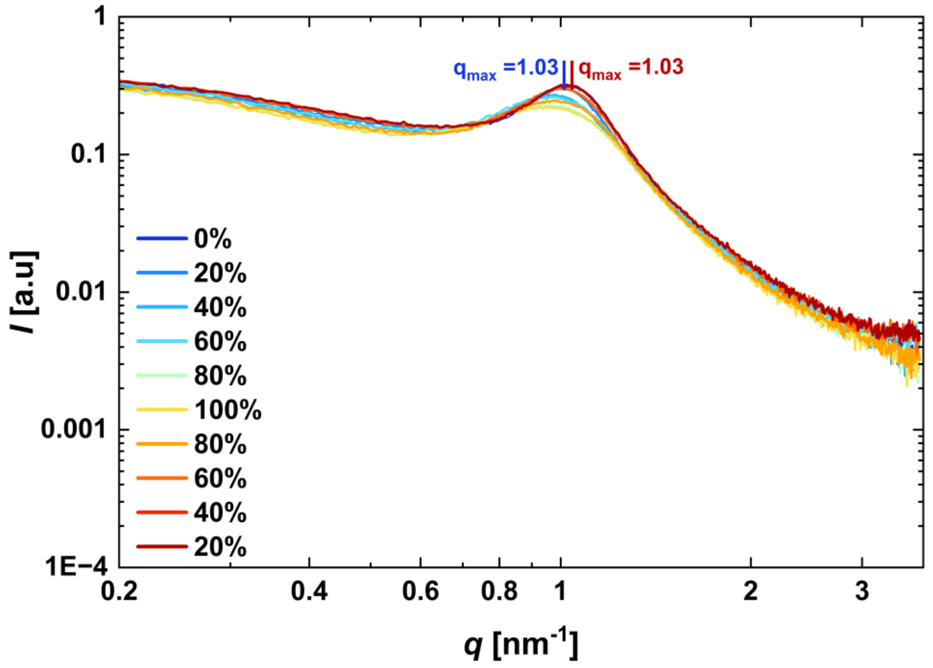


**Figure S85**. 1D-SAXS pattern for **CEPU3** as a function of strain during cycle elongation from 0% to 100% to 20% at 20% intervals.

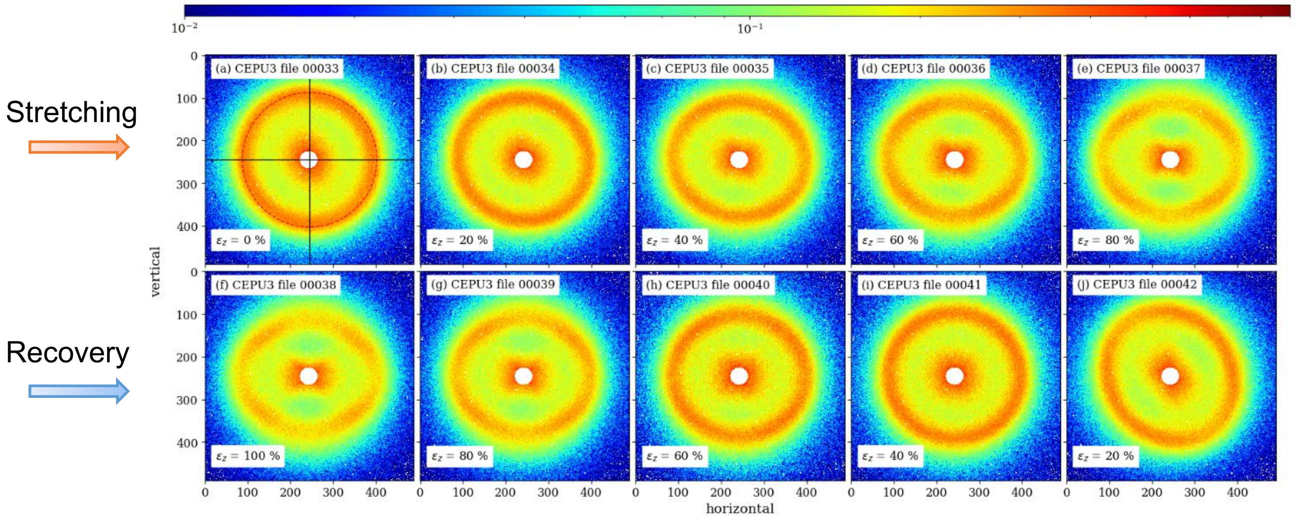


**Figure S86**. 2D-SAXS pattern for **CEPU3** as a function of strain during cycle elongation from 0% to 100% to 20% at 20% intervals.

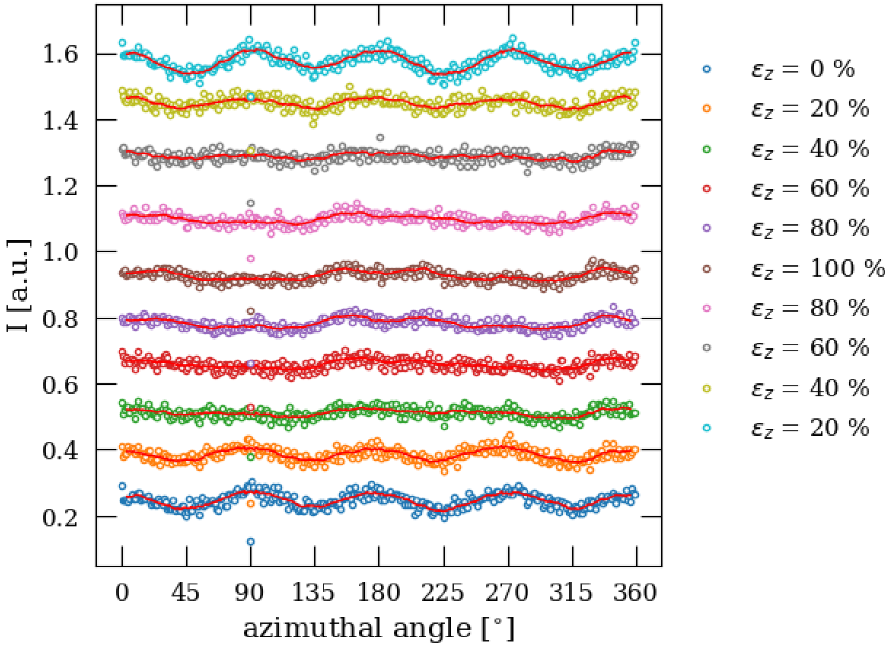


**Figure S87**. Azimuthal profile for the **CEPU3** elastomer as a function of strain during cycle elongation from 0% to 100% to 20% at 20% intervals.

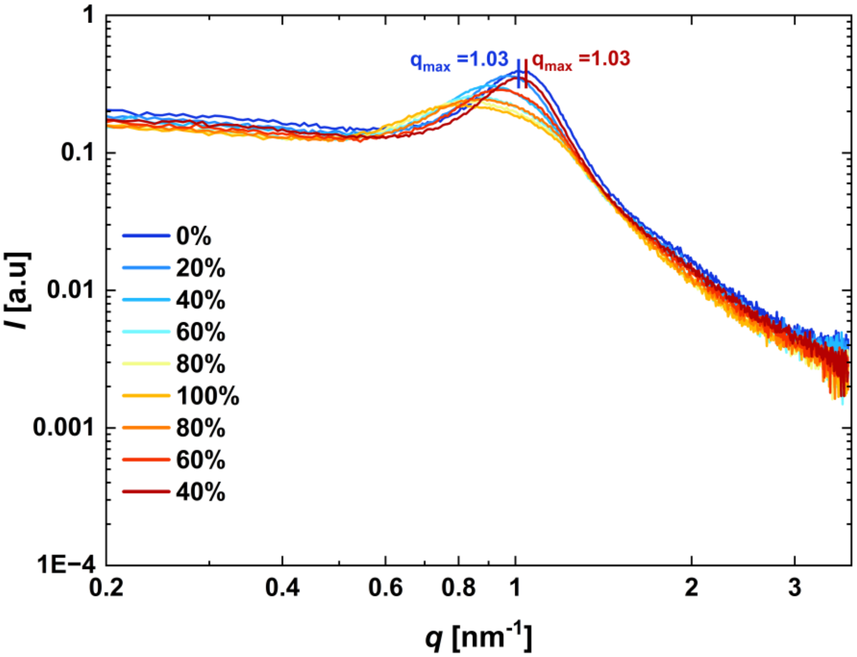


**Figure S88**. 1D-SAXS pattern for **CEPU4** as a function of strain during cycle elongation from 0% to 100% to 40% at 20% intervals.

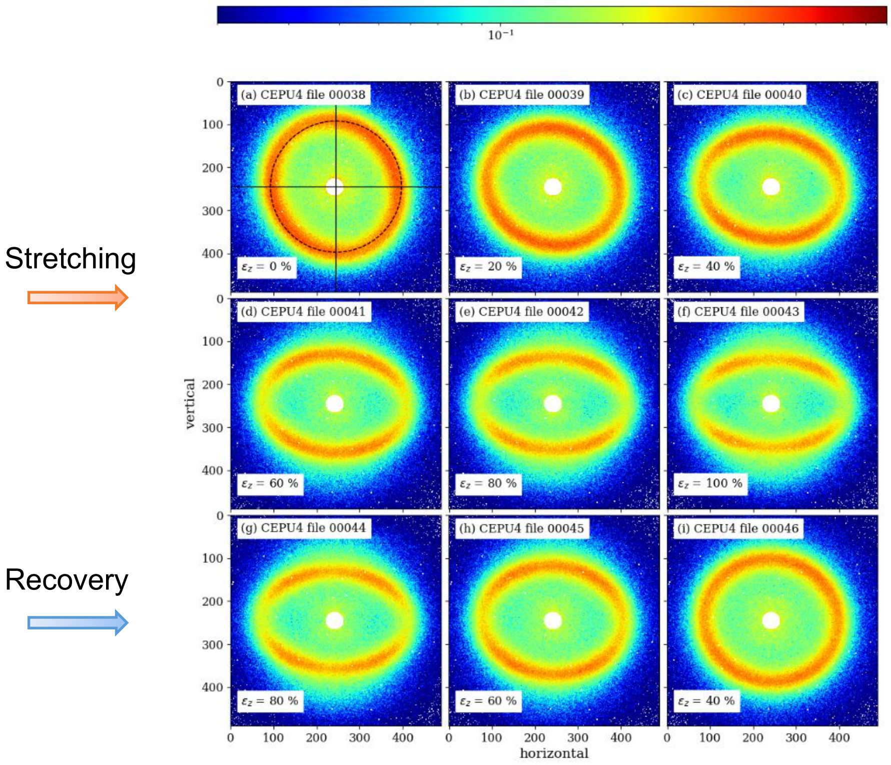


**Figure S89**. 2D-SAXS pattern for **CEPU4** as a function of strain during cycle elongation from 0% to 100% to 40% at 20% intervals.

The stress-strain relationship is produced using standard equations.[1] To investigate hysteresis and Mullins-like softening, cyclic compression tests were conducted, Figure S91(A), under constant crosshead speed control at 0.02 mm/s, corresponding to an approximate strain rate of 0.01 $s^{-1}$ using MTS screw driven load frame. The gauge section was marked before testing to enable post-test comparison and to assess whether the gauge length could recover to its original length after deformation. At the same time, rate-dependent compression tests were conducted to evaluate the rate-dependent energy absorption capability. Moreover, to assess the recoverability of these CEPUs, load-relax-reload protocols were applied, as shown in Figure S91(B). Specimens were first loaded to 20% or 40% true strain and then immediately unloaded. After unloading, they were allowed to recover for 1 min (20% true strain) or 6 min (40% true strain). The current dimensions of the specimens were then measured, and the samples were reloaded under the same loading profile. Tensile specimens had a gauge length of 33 ± 1 mm and a gauge width of 13 ± 0.5 mm. To ensure repeatability, each test was repeated at least three times. Elastic recovery[2] has been calculated as shown in Figure S91.

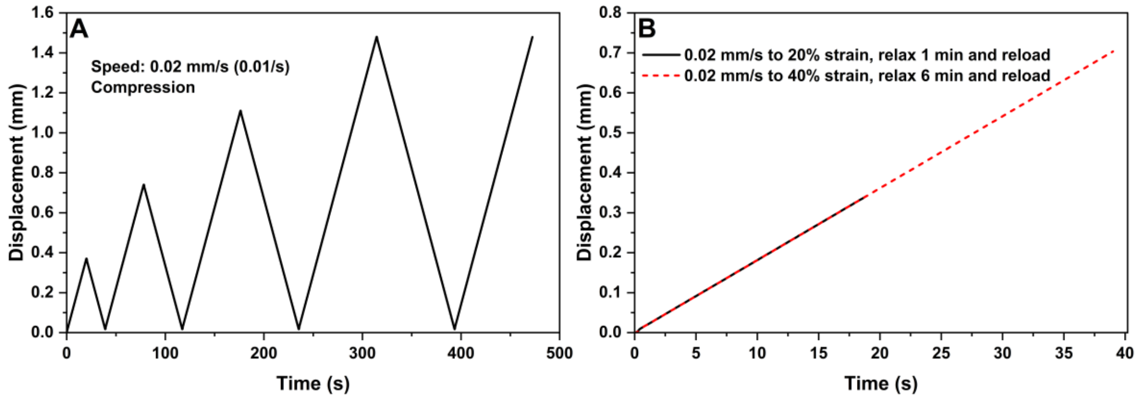


**Figure S90.** (A) cyclic compression (B) load-relax-reload compression.

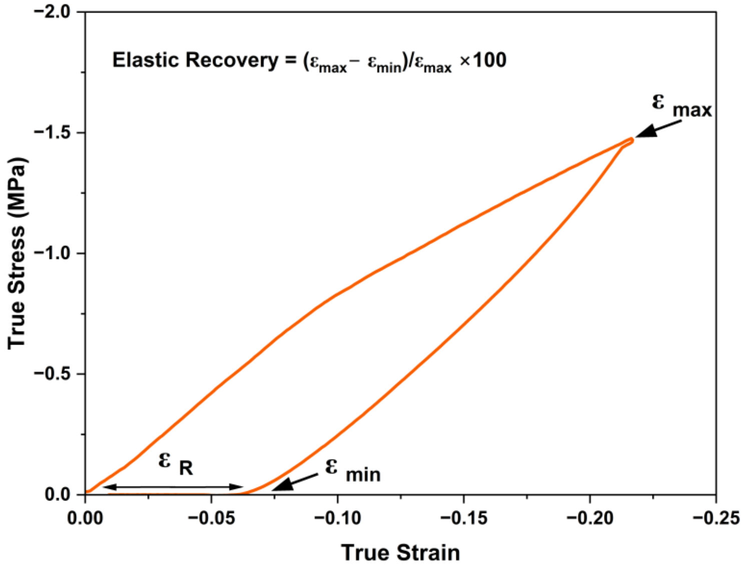


**Figure S91**. Parameters measured during cyclic tensile test. Calculation of elastic recovery (ER), $\varepsilon_{max}$ (maximum strain), $\varepsilon_{min}$ (minimum strain), residual strain ($\varepsilon_R$).[2]

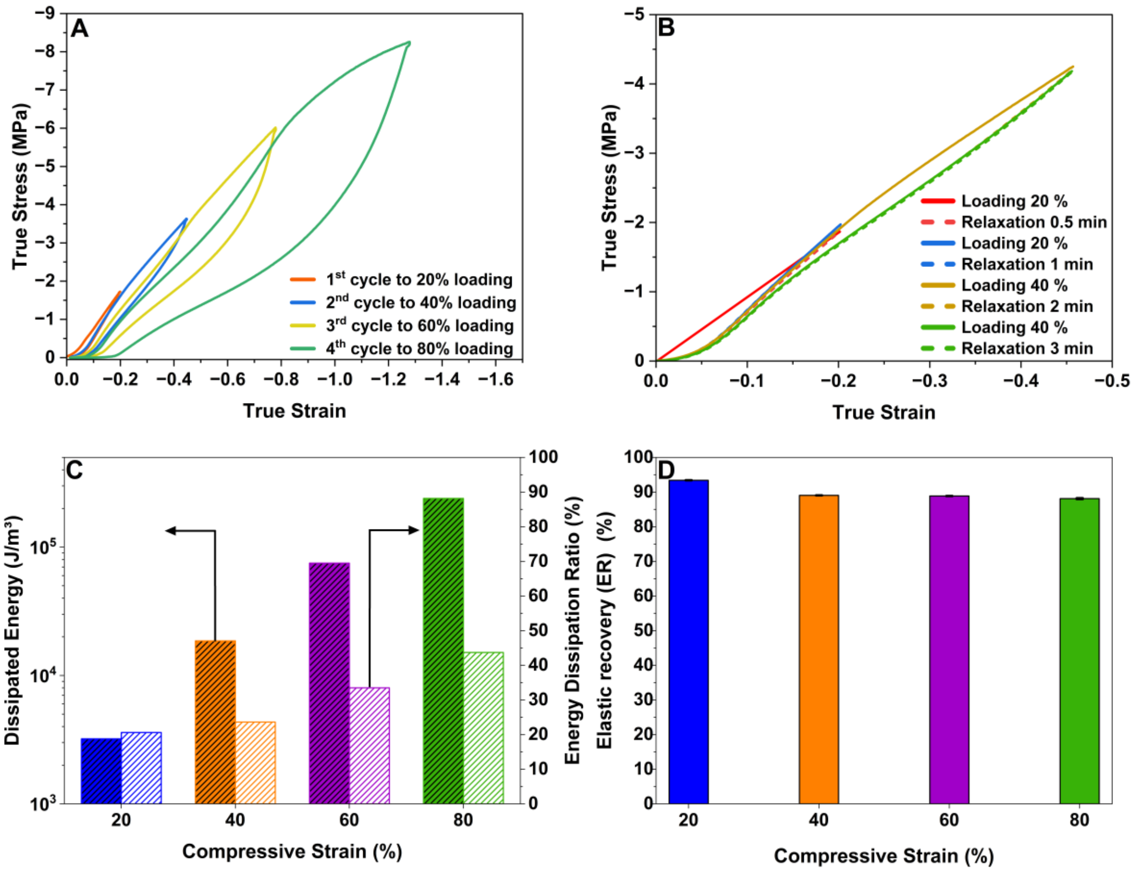


**Figure S92**. (A) Single cyclic compression tensile tests of **CEPU4** at 25 °C under various deformations of 20%, 40%, 60% and 80% in successive stretching, (B) Compression hysteresis energy recovery tests for **CEPU4** at two various deformation 20% and 40% at 25 °C, after relaxing, the cycle curve was overlapped with the original cycle (C) Compressive strain-dependent energy dissipation ratios and dissipated energies of **CEPU4**, and (D) Comparison of elastic recovery of **CEPU4**.

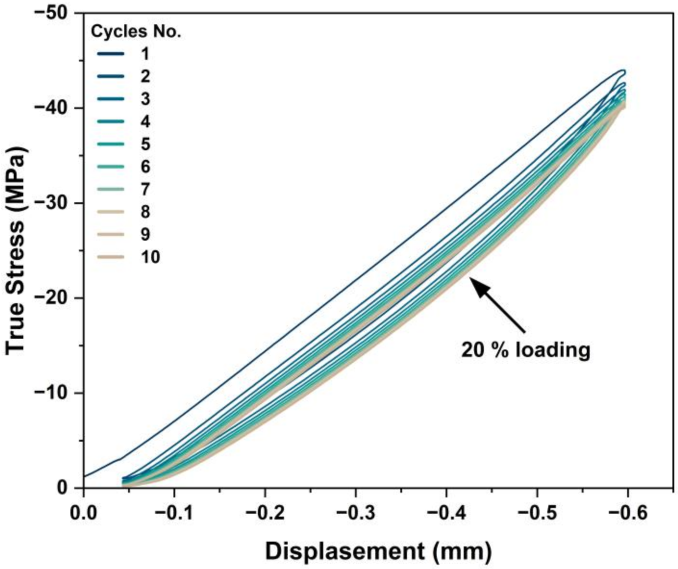


**Figure S93**. Consecutive compression cyclic tensile tests of **CEPU4** at 25 °C without relaxation, specimens were compressed ten times at 20% deformation.

For the Digital Image Correlation (DIC) measurements, a white background was produced on one face of the specimen using an airbrush and white paint, and the speckle pattern was made using black paint and a net, as seen in Figure S95 . To record the experiment a Point Grey camera with a 60 mm Nikon lense was utilized to obtain images for the DIC measurements. The frame rate that was used was 2 Hz.

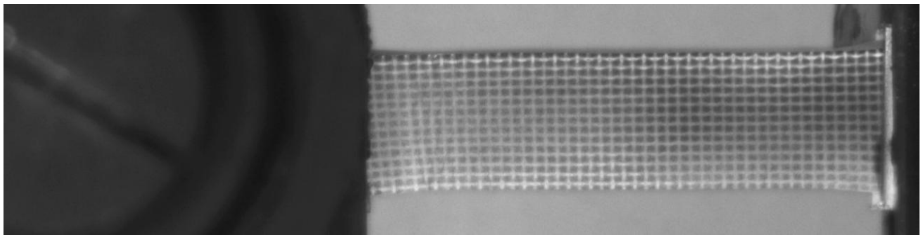

**Figure S94**. Speckle pattern used for DIC in tension tests.

Optical images were analysed using MatchID® (www.matchid.eu, version 2025.2) to obtain displacement and strain fields. The DIC-derived axial displacement agrees well with the crosshead displacement, confirming reliable tracking and indicating no obvious grip slip during testing. The strain fields show homogeneous deformation with no pronounced strain localisation, as illustrated in Figure S96. In addition, by assuming the out-of-plane transverse strain to be equal to the in-plane transverse strain, the DIC results indicate that

the specimen deforms nearly isochorically during stretching (Figure S97), supporting the use of analytical relations to extract the true stress–strain response.

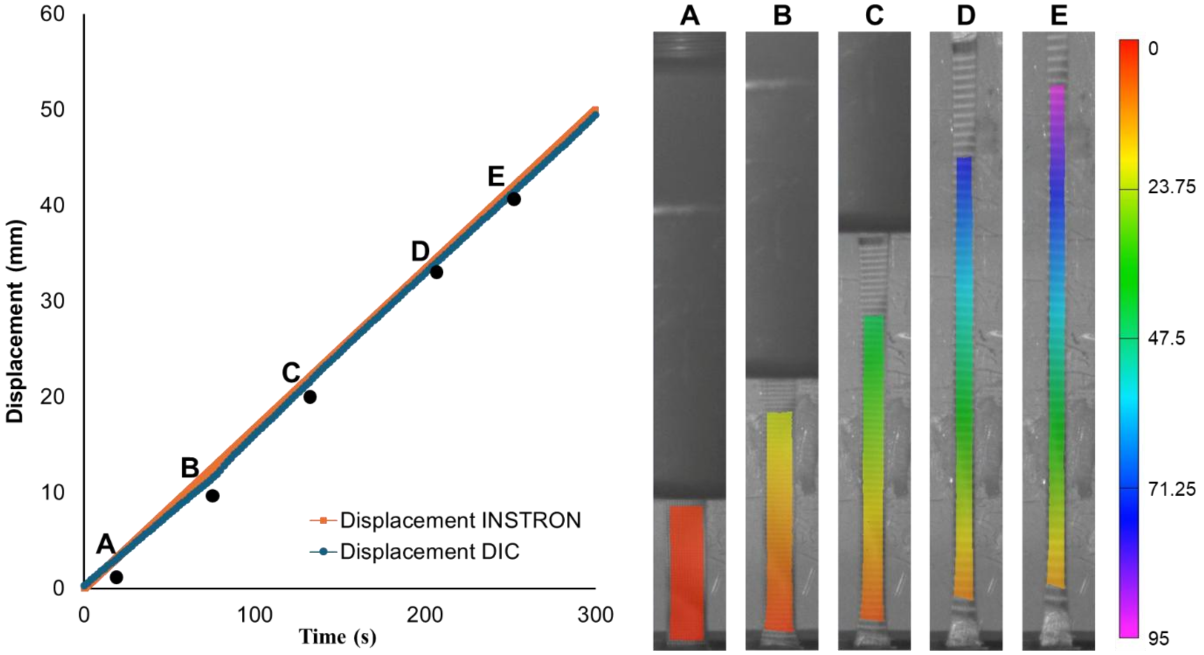


**Figure S95**. Comparison of DIC and crosshead displacements.

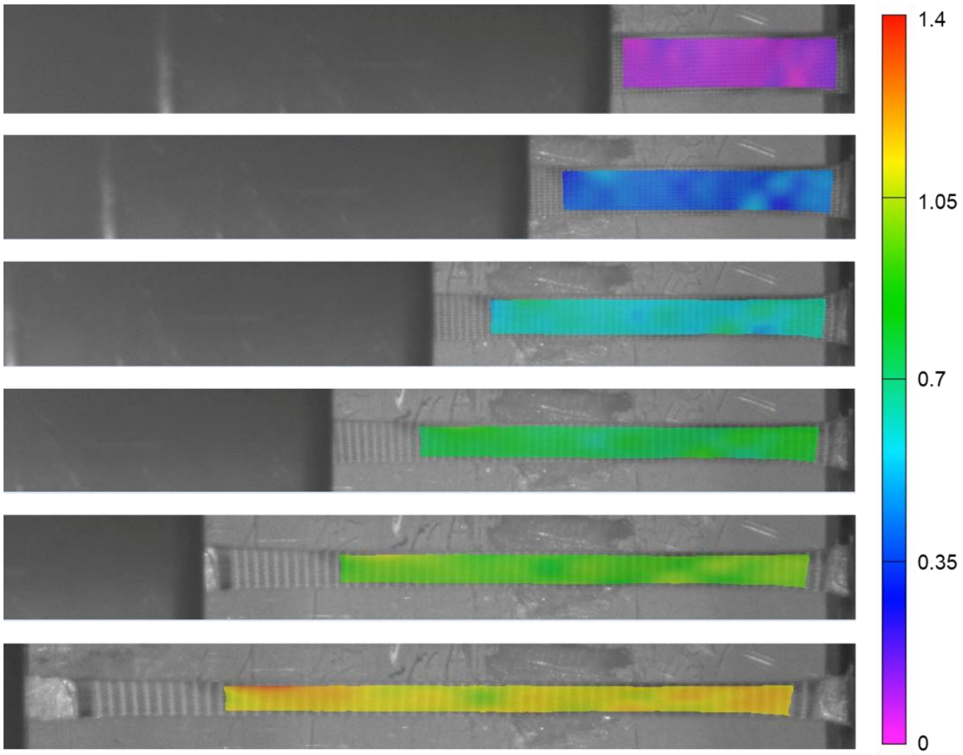


**Figure S96**. Strain field evolution on the surface of the CEPU specimen.

## Validation of homogeneous strains assumption

This validation of this assumption was done by checking the distribution of $e_{xx}$ strains along the height of the specimen's cross section, as seen in Figure S98. The same was done for multiple different cross sections.

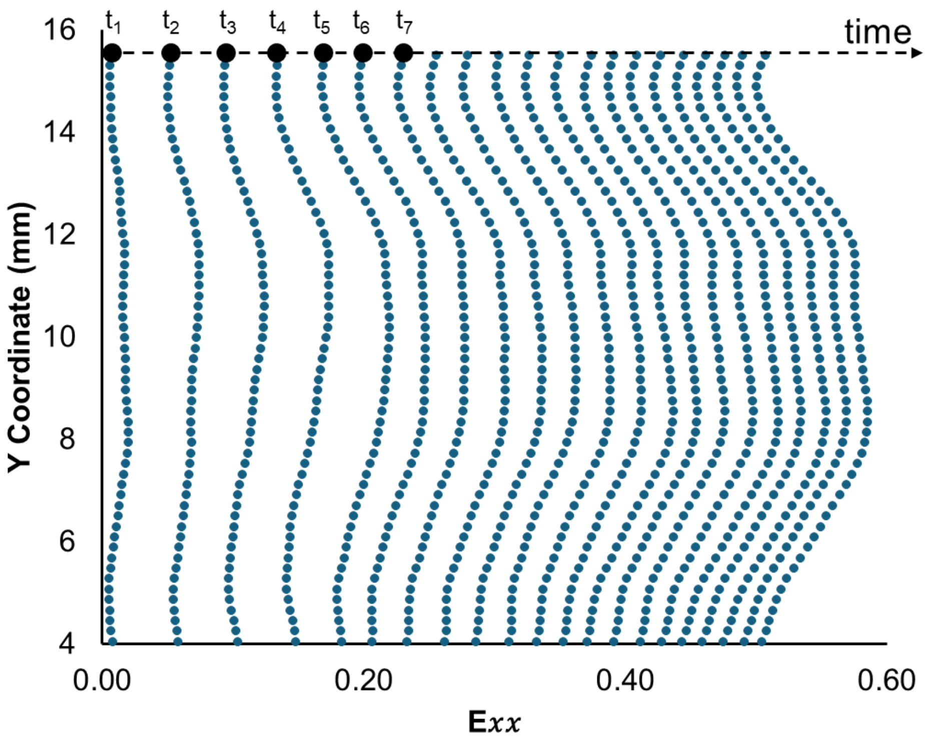


**Figure S97**. Axial deformation over a cross-section of the specimen during the tensile test.

**Validation of incompressibility assumption**

For the incompressibility, the change in volume in function of time was calculated using Equation S1:

$$\frac{V}{V_0} = (e_{xx} + 1) \cdot (e_{yy} + 1)^2 \qquad \text{(Equation S1)}$$

Where $e_{xx}$ and $e_{yy}$ are engineering strains for the loading and in-plane transverse respectively, and again the assumption that $e_{zz} = e_{yy}$ was made. The results are shown in Figure S99 where the assumption regarding incompressibility is validated since the volume remains almost the same in function of time, even for very large strains.

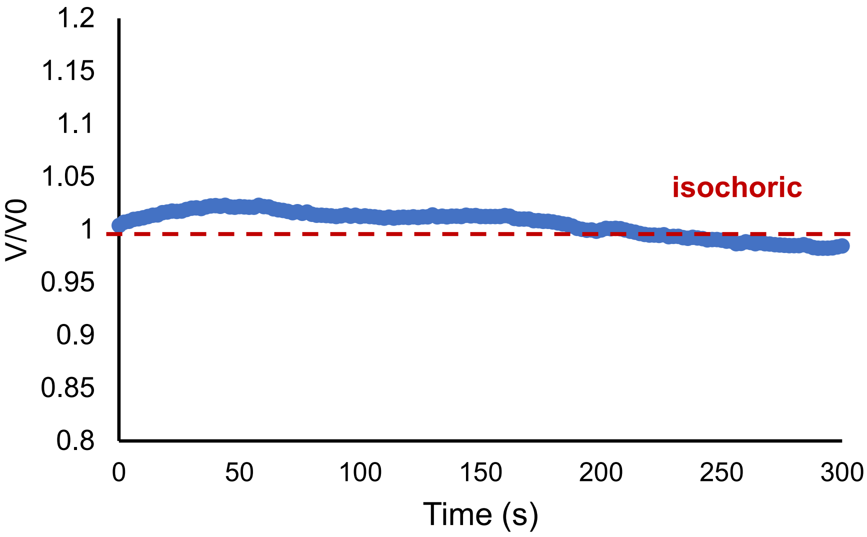


**Figure S98**. Incompressibility (Volume Conservation) V'/V over a cross-section of the specimen during the tensile test.

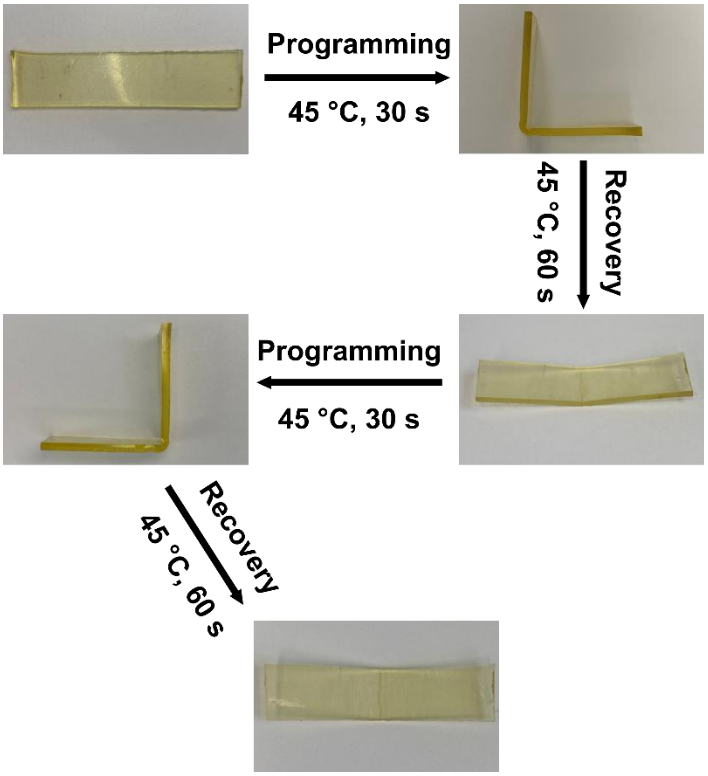


**Figure S99**. Photographs depicting shape memory performance of **CEPU3**.

**References**


(1) George T. (Rusty) Gray, III, Classic Split-Hopkinson Pressure Bar Testing, *Mechanical Testing and Evaluation*, Vol 8, *ASM Handbook*, Edited By Howard Kuhn, Dana Medlin, *ASM International*, **2000**, 462–476. DOI: 10.31399/asm.hb.v08.a0003296.

(2) Gao, C.; Poon, K. C.; Concilio, M.; Zinn, T.; Gregory, G. L.; Williams, C. K. High-Performance Recyclable Polyester Elastomers Through Transient Strain-Stiffening. *Adv. Mater.* **2025**, 37, 2416674. DOI:10.1002/adma.202416674.
